# Causal Interplay Between Platelet Indices and Rheumatoid Arthritis: Genetic Evidence From Bidirectional Mendelian Randomization

**DOI:** 10.1155/ijog/4549330

**Published:** 2026-07-30

**Authors:** Xu-Yan Shen, Jia-Ying Sun, Xin Wang, Shu-Shan Zhao

**Affiliations:** ^1^ Department of Rheumatology and Immunology, Shaoxing People′s Hospital, Shaoxing, Zhejiang Province, China

**Keywords:** mean platelet volume, Mendelian randomization, plateletcrit, platelets, rheumatoid arthritis

## Abstract

**Objectives:**

This study is aimed at comprehensively understanding genetically causal associations between some platelet indices (PIs) and rheumatoid arthritis (RA).

**Methods:**

Genetic summary statistics for the 4 types of PIs and RA were derived from Neale Lab, FinnGen, and MRC‐IEU consortium. Two‐sample Mendelian randomization (TSMR) analysis was utilized to infer the bidirectional causality with the implementation of the inverse‐variance weighted (IVW), weighted median (WM), weighted mode, and MR‐Egger methods. Sensitivity analysis with leave‐one‐out method was conducted to assess the robustness of the observed causal estimates.

**Results:**

TSMR results indicated that the genetically‐determined plateletcrit (PCT) had a causal association with the higher risk of RA (odds ratio [OR] = 1.13, 95% confidence interval [CI]: 1.01, 1.29, *p* = 0.012) and seropositive RA (OR = 1.03, 95% CI: 1.01, 1.21, *p* = 0.003). Both WM method and sensitivity analysis supported that the observed causal estimates were reliable. In the reverse MR analysis, genetic susceptibility leading to RA (Beta [se] = −0.012 [0.006], *p* = 0.037) was causally related to the decrease in mean platelet volume (MPV).

**Conclusions:**

Our study reveals a positive causal association between PCT and the risk of RA, and that genetic susceptibility to RA is causally associated with a reduced MPV. This provides a reference for further studies on the exploration of mechanisms of platelets in the pathogenesis of RA.

## 1. Introduction

Rheumatoid arthritis (RA) is a chronic systemic autoimmune disease, primarily characterized by symmetric inflammatory polyarthritis that leads to the erosion of articular cartilage and bone [1, 2]. An estimated 1% of the global population is affected by RA [3]. The disease causes debilitating pain, joint stiffness, and functional impairment, and can lead to systemic complications involving multiple organs [4]. It also imposes a substantial economic burden through healthcare costs, work disability, and lost productivity [5]. To date, the precise etiology and pathogenesis of RA remain incompletely understood. However, evidence indicates that genetic susceptibility, environmental factors, and infections contribute to its onset and progression [6–8].

Platelets are anucleate cells derived from megakaryocytes that circulate in the blood [9]. As essential components of the circulatory system, they play crucial roles in maintaining vascular integrity, preventing excessive bleeding, and supporting other physiological processes [10]. Over the past 2 decades, growing evidence has recognized the involvement of platelets in immune regulation and inflammation. Platelets can interact with and influence both innate and adaptive immune cells, thereby acting as drivers of immune dysregulation [11]. In fact, associations between platelet‐related disorders and RA have been widely reported [12, 13]. Previous studies have shown elevated platelet counts (PLTs) in the synovial fluid of RA patients, correlating with rheumatoid factor (RF) concentration [14]. Additionally, Coban et al. [15] reported significantly increased mean platelet volume (MPV) in RA patients and suggested links between inflammation, platelet activation, and arterial thrombosis. Further evidence indicates that platelets represent a potential therapeutic target in RA, participating in systemic inflammatory responses and arterial thrombosis [16]. However, most prior studies on the relationship between platelet indices (PIs) and RA have been observational and cannot establish causality. Whether a genetic causal association exists between PIs and RA remains unclear.

Mendelian randomization (MR) is a genetic epidemiological method that uses genetic variants as instrumental variables (IVs) to infer causal relationships between exposures and outcomes. Compared with observational studies, MR better controls for confounding and reverse causation, providing stronger genetic evidence for disease prevention and treatment strategies [17]. In this study, we applied a bidirectional two‐sample MR (TSMR) analysis to investigate the causal relationships between PIs and RA. This approach allows us to assess both the effect of genetically influenced PIs on RA risk and the potential effect of RA liability on PIs. These findings may offer valuable insights for developing therapeutic targets and preventive strategies for RA.

## 2. Methods

### 2.1. Study Design

In MR analysis, single‐nucleotide polymorphisms (SNPs) serve as IVs to represent genetic variation [17]. These IVs enable the assessment of causal links between exposures and outcomes. To obtain valid estimates, all selected IVs must satisfy three key assumptions: (1) relevance assumption, strong association with the exposure [18]; (2) independence assumption, no association with confounders of the exposure‐outcome relationship [19]; and (3) exclusion restriction assumption, influence on the outcome only through the exposure, not via alternative pathways [20].

**Figure 1:**
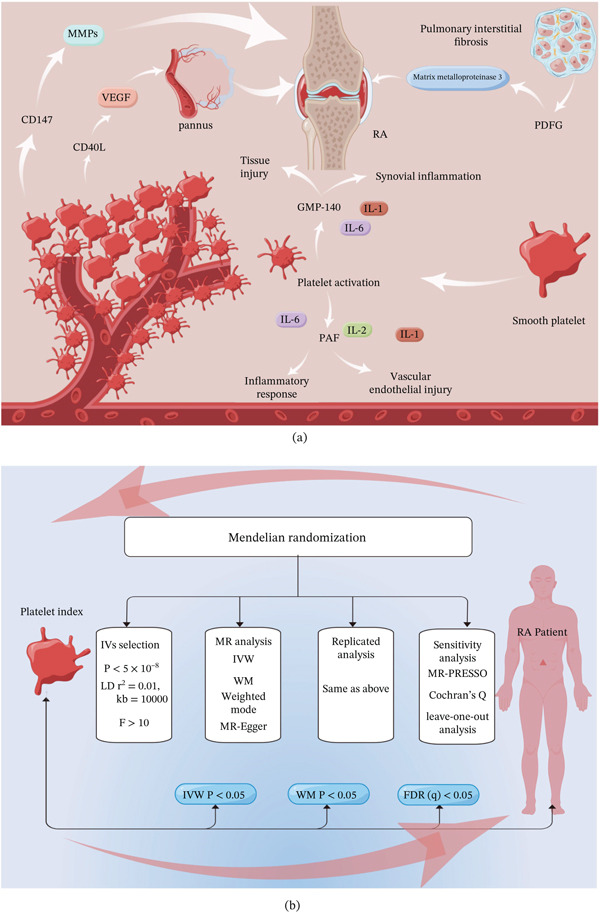
The possible mechanism and study design of MR analysis between PIs and RA. (a) The possible mechanism of platelet and RA. (b) The flow chart of causal associations MR analysis. Abbreviations: IVW, inverse variance weighting; MPV, mean platelet volume; MR, Mendelian randomization; MR‐PRESSO, MR pleiotropy residual sum and outlier; PCT, plateletcrit; PDW, platelet distribution width; PIs, platelet indices; PLT, platelet count; RA, rheumatoid arthritis; WM, weighted median.

Given the recognized links between platelets and RA, we applied a bidirectional TSMR design to assess the causal associations of four PIs with the risk of RA, including PLT, plateletcrit (PCT), MPV, and platelet distribution width (PDW). We further validated the initial findings using an independent RA cohort from the Finnish population to ensure reliability. A flowchart summarizing the hypothesized mechanisms and the MR analysis procedure was presented in Figure 1.

### 2.2. Data Sources

Genetic summary statistics for the four kinds of PIs and RA were obtained from publicly available genome‐wide association studies (GWAS), detailed in Table 1. Genetic variants associated with the PIs came from the largest published GWAS meta‐analysis by the Neale lab, based on European‐ancestry participants with sample sizes as follows: PLT (*N* = 350,474), PCT (*N* = 350,471), MPV (*N* = 350,470), and PDW (*N* = 350,470). Genetic data for RA phenotypes were sourced from two consortia: the MRC‐IEU consortium provided summary statistics for RA and seropositive RA (*N* = 58,284 and *N* = 177,430, respectively), whereas the FinnGen consortium provided data for seronegative RA and other RA (*N* = 174,841 and *N* = 217,314, respectively) [21]. All data were derived from European‐ancestry populations. Sample overlap between PIs and RA datasets was minimal (overlap < 10%), reducing the risk of bias from sample overlap [22].

**Table 1:** The detailed information regarding the genetic datasets used in the present study.

Exposure/outcome	Population ancestry	Sample size	Consortium	GWAS ID
PLT	European	350474	Neale lab	ukb‐d‐30080_irnt
PCT	European	350471	Neale lab	ukb‐d‐30090_irnt
MPV	European	350470	Neale lab	ukb‐d‐30100_irnt
PDW	European	350470	Neale lab	ukb‐d‐30110_irnt
RA	European	58284	MRC‐IEU	ieu‐a‐832
Other RA	European	217314	FinnGen	finn‐b‐RHEUMA_NOS
Seropositive RA	European	177430	FinnGen	finn‐b‐RHEUMA_SEROPOS
Seronegative RA	European	174841	FinnGen	finn‐b‐RHEUMA_SERONEG

Abbreviations: MPV, mean platelet (thrombocyte) volume; PCT, plateletcrit; PDW, platelet distribution width; PLT, platelet count; RA, rheumatoid arthritis.

### 2.3. IV Selections

We selected SNPs associated with each PI at a genome‐wide significance threshold (*p* < 5 × 10^−8^). To ensure independence among instruments, we then clumped these SNPs to remove linkage disequilibrium (LD) using a cutoff of *r* ^2^ < 0.01 within a 10,000 kb window. Exposure and outcome data were harmonized, and ambiguous or palindromic SNPs were excluded. radial MR was used to identify and remove potential outliers. For IVs unavailable in the outcome dataset, we identified proxy SNPs using LD (*R* ^2^) > 0.8. The strength of each instrument was assessed using the *F*‐statistic, calculated as

F=R2×n−k−1/k×1−R2,R2=2×1−MAF×MAF×beta2,

where *R* ^2^ is the proportion of variance explained [23]. An *F* − statistic > 10 was considered evidence against weak instrument bias (see Table S1). Statistical power was estimated using the online tool “mRnd” (https://shiny.cnsgenomics.com/mRnd/).

### 2.4. Ethics Statement

All data used in this study were publicly available GWAS abstracted data; therefore, no additional informed consent or ethical approval was required.

### 2.5. Statistical Analysis

#### 2.5.1. MR Analysis

We first performed forward MR analyses to examine the causal effects of four kinds of PIs on RA risk. The primary analysis used the inverse variance weighted (IVW) method under a random‐effects model. We supplemented this with three additional methods: weighted median (WM), weighted mode, and MR‐Egger regression [24]. The IVW method provides consistent estimates in the absence of horizontal pleiotropy. To address potential pleiotropy, the WM method was applied, which obtains a robust estimate even if up to 50% of the instruments are invalid. We considered a result statistically significant when both IVW and WM yielded *p* < 0.05. To control for multiple testing across the several exposures–outcome pairs examined, we applied false discovery rate (FDR) correction. A causal association was deemed robust if the FDR‐adjusted *p* value for the IVW estimate was < 0.05. It is important to note that the FDR correction was applied specifically to the primary causal tests (i.e., the IVW‐derived *p* values across all tested exposure‐outcome pairs in the bidirectional MR analysis). Sensitivity and pleiotropy analyses (e.g., MR‐Egger intercept test, MR pleiotropy residual sum and outlier (MR‐PRESSO) global test, Cochran′s *Q* test) are diagnostic in nature and were therefore not subjected to multiple testing correction. Consequently, although our main causal inferences are robust to false positives, the interpretation of *p* values from these secondary diagnostic tests should be made with an awareness of potential residual Type I error.

To assess potential reverse causation, we performed bidirectional MR analyses with RA as the exposure and each PI as the outcome. As RA is a binary trait and PIs are continuous, we converted odds ratios (ORs) to beta coefficients using the formula Beta = log (OR). This beta coefficient represents the change in the PI per unit increase in the log‐odds of RA.

#### 2.5.2. Sensitivity Analysis

We conducted sensitivity analyses to evaluate the robustness of the MR estimates. Horizontal pleiotropy was assessed using the MR‐PRESSO method; a global test *p* > 0.05 indicated no evidence of pleiotropy [19]. Heterogeneity across instrumental variants was quantified with Cochran′s *Q* test [25]. We also performed leave‐one‐out analysis by iteratively removing each SNP to determine whether any single variant disproportionately influenced the overall causal estimate [26]. All analyses were performed in R (Version 4.2.2) using the “TwoSampleMR” package.

## 3. Results

In the forward MR analysis, we identified 1551 SNPs associated with four kinds of PIs as IVs, including 422 for PLT, 384 for PCT, 419 for MPV, and 326 for PDW. These SNPs explained approximately 19%, 15%, 32%, and 19% of the phenotypic variance for each PI, respectively. For the reverse MR analysis, 70 SNPs associated with RA were included, collectively accounting for about 18% of its genetic variance. The *F*‐statistic for every SNP exceeded 10, indicating sufficient instrument strength and minimal weak instrument bias. Sample overlap between PIs and RA datasets was low (< 5%), reducing the likelihood of bias from sample overlap (Table S1). Statistical power for the tested causal associations was generally adequate, ranging from 0.79 to 0.99 across the analyzed PI‐RA pairs (Figure S1).

### 3.1. Causal Associations of PIs With Risk of RA

**Figure 2:**
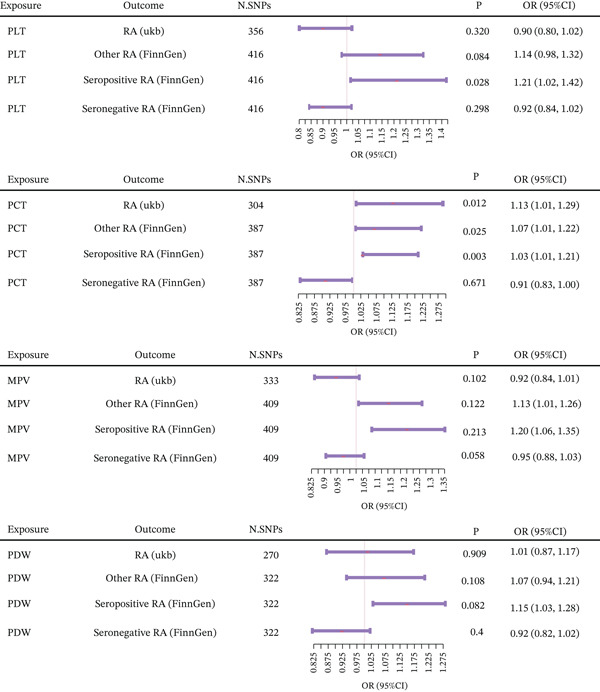
Forest plot of causal estimates between four PIs and risk of RA. The width of the confidence intervals reflects the precision of the estimates; intervals crossing the null line (OR = 1 or Beta = 0) indicate a lack of statistical evidence for a causal effect. Abbreviations: MPV, mean platelet volume; MR, Mendelian randomization; OR, odds ratio; PCT, plateletcrit; PDW, platelet distribution width; PLT, platelet count; RA, rheumatoid arthritis; SNP, single nucleotide polymorphism; ukb, UK biobank.

**Figure 3:**
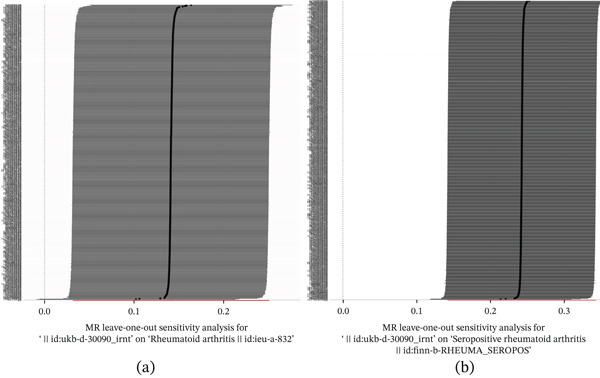
Sensitivity analyses with leave‐one‐out method for the causal effects of PCT on RA risk. (a) Sensitivity analysis for the association of PCT on RA. (b) Sensitivity analysis for the association of PCT on seropositive RA. Abbreviations: PCT, plateletcrit; RA, rheumatoid arthritis.

The results of MR analysis showed that genetically predicted increase in PCT was causally associated with an elevated risk of developing RA in utilizing both IVW and WM methods (OR_IVW_ = 1.13, 95% CI: 1.01, 1.29, *p* = 0.012; OR_WM_ = 1.21, 95% CI: 1.02, 1.43, *p* = 0.008) (Figure 2 and Table S2). This finding was further confirmed by the results of FDR correction (Figure S2). Although Cochran′s *Q* test indicated significant heterogeneity (*p* < 0.05), the primary IVW analysis was performed under a random‐effects model, which provides a conservative estimate that accounts for such heterogeneity. The WM method, which yields robust estimates even when up to 50% of the instruments are invalid, produced a result consistent with the IVW estimate. Moreover, the MR‐Egger intercept test showed no evidence of directional pleiotropy (*p* > 0.05; Table S2). Sensitivity analyses, including radial MR and leave‐one‐out analysis, confirmed that no single SNP disproportionately influenced the causal estimates (Figure 3). In addition, we did not observe any causal relationships between the other three PIs and RA risk (Figure 2 and Tables S3, S4 and S5).

### 3.2. Causal Associations of RA With PIs

**Figure 4:**
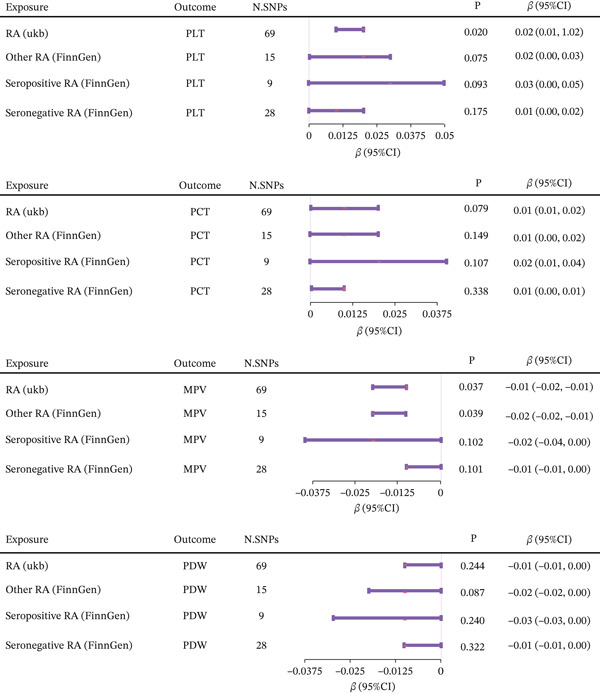
Forest plot of causal estimates between RA on four PIs. The width of the confidence intervals reflects the precision of the estimates; intervals crossing the null line (OR = 1 or Beta = 0) indicate a lack of statistical evidence for a causal effect. Abbreviations: MPV, mean platelet volume; MR, Mendelian randomization; OR, odds ratio; PCT, plateletcrit; PDW, platelet distribution width; PLT, platelet count; RA, rheumatoid arthritis; SNP, single nucleotide polymorphism; ukb, UK biobank.

**Figure 5:**
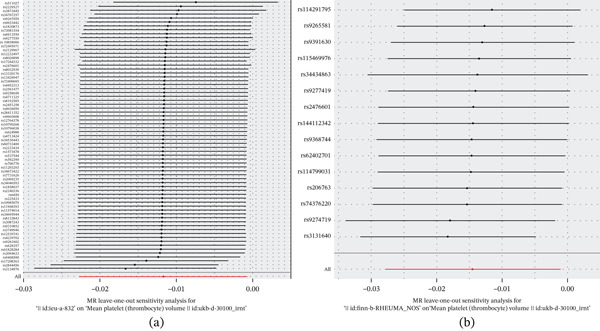
Sensitivity analyses with leave‐one‐out method for the causal effects of RA on MPV. (a) Sensitivity analysis for the association of RA on MPV. (b) Sensitivity analysis for the association of other RA on MPV. Abbreviations: MPV, mean platelet volume; RA, rheumatoid arthritis.

For the purpose of assessing the bidirectional causality between RA and PIs, we set RA as an exposure factor to test reverse causal effects of RA on PIs. A reverse causal effect of genetically determined RA on a decrease in MPV was found in the implementation of IVW and WM approaches (Beta_IVW_ (se) = −0.02 (0.01), *p* = 0.037; Beta_WM_ (se) = −0.01 (0.01), *p* = 3.17 × 10^−4^) (Figure 4 and Table S9), and the causal estimates were still significant after FDR correction (Figure S2). Despite significant heterogeneity detected by Cochran′s *Q* test (*p* < 0.05), the random‐effects IVW model provided a conservative estimate. The WM method yielded a highly consistent effect size. The MR‐Egger intercept test was not significant (*p* > 0.05; Table S9), indicating no detectable directional pleiotropy. MR‐PRESSO global test and leave‐one‐out analysis further confirmed that the observed association was not driven by any single outlier variant (Figure 5). Moreover, we did not find any causal associations between RA and other PIs (Tables S6, S7 and S8).

### 3.3. Causality Validation in Independent Consortia

To further validate the observed causality, the additional genetic summary data of RA were sourced from the Finnish population, which consists of three RA‐relevant phenotypes, including other RA, seropositive RA, and seronegative RA. We repeated the same steps of the aforementioned IVs selection and analytical process to validate the consistency and reliability of causal estimates.

In the forward direction, a solid positive causal association was suggested between genetically predicted PCT and the higher risk of seropositive RA (OR_IVW_ = 1.03, 95% CI: 1.01, 1.21, *p* = 0.003; OR_WM_ = 1.06, 95% CI: 1.01, 1.21, *p* = 0.002), which is consistent with the previously observed findings (Figure 2 and Table S2). Interestingly, we also noticed a genetically causal association between PLT and seropositive RA (OR_IVW_ = 1.21, 95% CI: 1.02, 1.42, *p* = 0.028; OR_WM_ = 1.01, 95% CI: 1.01, 1.28, *p* = 0.032) (Figure 2 and Table S3); this association was not significant after the FDR correction (Figure S2).

Conversely, the results of reverse MR analysis supported a genetic causal association between other RA and MPV after FDR correction (Beta_IVW_ (se) = −0.02 (0.01), *p* = 0.039; Beta_WM_ (se) = −0.02 (0.02), *p* = 9.39 × 10^−7^) (Figure 4 and Table S9). Further sensitivity and radial MR analyses showed no evidence of horizontal pleiotropy or influential outliers, suggesting that the causal estimates were stable (Figure 5 and Table S9).

## 4. Discussion

Our bidirectional TSMR analysis provides novel genetic evidence supporting a causal interplay between specific PIs and RA. We identified two key findings: first, a genetically determined increase in PCT is causally associated with a higher risk of RA; second, genetic susceptibility to RA is causally linked to a decrease in MPV. These results suggest that platelets are not merely passive reactants but may play an active, genetically influenced role in RA pathogenesis.

The observed positive causal link between PCT and RA risk suggests that a genetically determined increase in the total platelet mass may be a contributing factor to RA pathogenesis. One plausible mechanism involves enhanced platelet activation. Higher platelet mass (reflected by PCT) could lead to an increased burden of activated platelets within the synovial microvasculature [27]. Activated platelets release a plethora of pro‐inflammatory mediators such as CD40L, P‐selectin, serotonin, and a wide array of chemokines [28]. These factors can recruit and activate leukocytes, stimulate synovial fibroblast proliferation, and promote a pro‐inflammatory endothelial phenotype, thereby fueling the inflammatory cascade and joint destruction characteristic of RA [29]. Conversely, the reverse causal relationship, where genetic liability to RA leads to reduced MPV, offers a new perspective on how chronic inflammation may remodel platelet biology. Chronic exposure to inflammatory cytokines (e.g., IL‐6, TNF‐*α*) in RA could directly influence bone marrow megakaryopoiesis, favoring the production of smaller platelets [30, 31]. Although platelet consumption during active inflammation might theoretically increase the proportion of larger, younger platelets, our finding suggests a dominant suppressive effect of the RA milieu on platelet size [32]. This warrants further investigation into the specific effects of inflammation on platelet production and turnover.

Although further investigation is required to clarify the specific effects of inflammation on platelet production and turnover, our findings establish a robust genetic causal framework that overcomes key limitations of observational studies, such as confounding and reverse causation. The causal effect sizes we observed are modest, as expected for polygenic traits like RA; however, their primary significance lies in confirming a bidirectional causal relationship between PIs and RA. This elevates the platelet‐RA association from correlation to a genetically supported causal link, pinpointing PCT and MPV as critical nodes where genetic determinants intersect with RA biology and highlighting promising targets for deeper mechanistic exploration.

Our results help contextualize and clarify conflicting reports from observational studies on PIs in RA. Although many studies have reported associations between various PIs (PLT, MPV, PDW) and RA disease activity, the findings have been inconsistent [33–37]. Some meta‐analyses even found no significant difference in MPV or PDW between RA patients and controls [38]. Our genetic null findings for PLT and PDW in the forward direction suggest that these indices may not be causal risk factors for RA onset but are more likely secondary indicators of inflammation or disease activity, explaining their variability in observational settings. Importantly, our reverse MR finding that RA causally lowers MPV provides a genetic explanation for the observed reductions in MPV in active RA reported in some studies, framing it as a potential consequence rather than a cause of the disease state.

Our study has several strengths. To our knowledge, this is the first study to provide bidirectional genetic causal evidence linking PIs and RA, moving beyond prior observational reports that could not establish causality. Our MR design effectively minimized confounding and reverse causation, and the application of multiple complementary sensitivity analyses (WM, MR‐Egger, MR‐PRESSO, leave‐one‐out analysis) ensured the robustness of the causal estimates. Furthermore, the independent replication of the key findings in the FinnGen cohort substantially strengthens the reliability and generalizability of our results.

Several limitations must be acknowledged. First, there is some unavoidable heterogeneity in our study, which may be due to the differences in genotyping platforms and phenotyping methods used for PIs and RA. Despite observing significant heterogeneity in some analyses (as indicated by Cochran′s *Q* test), the consistent direction and significance of causal estimates across primary and robust supporting methods, coupled with the absence of influential outliers in leave‐one‐out analyses, suggest that the core causal inferences are reliable. The observed heterogeneity may stem from several sources: (1) the inherent biological diversity within the broad diagnostic category of RA, encompassing different serological subtypes (e.g., seropositive *vs.* seronegative), disease activity states, age of onset, and sex distribution, which may interact variably with platelet biology; (2) potential population stratification within the large European‐ancestry GWAS cohorts used; and (3) differences in laboratory methodologies or genotyping platforms across the original studies contributing to the GWAS meta‐analyses. The use of a random‐effects IVW model, which provides a conservative estimate under heterogeneity, mitigates some concerns regarding its impact on our conclusions. Furthermore, the convergence of estimates from the random‐effects IVW and pleiotropy‐robust WM methods, alongside nonsignificant MR‐Egger intercept tests, indicates that the identified heterogeneity does not invalidate the underlying causal inferences.

Second, as the GWAS summary data for both PIs and RA were sourced exclusively from individuals of European ancestry, the generalizability of our findings to other racial or ethnic groups is limited. Genetic architectures, allele frequencies, and LD patterns can differ across populations, which may affect the transferability of IVs and causal estimates. Therefore, our results require validation in diverse populations to confirm their broader applicability and to explore potential population‐specific genetic interactions.

Third, MR analyses can only provide corresponding genetic evidence for a causal relationship between PIs and RA, but the levels of PIs may be influenced by multifactorial effects. Further study with diverse populations and consideration of additional factors are essential for the validation of our results.

## 5. Conclusions

In conclusion, our study demonstrated the presence of causal relationships between genetic susceptibility to RA and MPV, as well as genetically determined PCT and the risk of RA. Our findings provide a comprehensive understanding of the intricate interplay between PIs and RA, shedding light on the underlying mechanisms of this complex disease.

**Nomenclature**
AS: ankylosing spondylitis
CI: confidence interval
FDR: false discovery rate
GWAS: genome‐wide association studies
IVs: instrumental variables
IVW: inverse variance weighted
LD: linkage disequilibrium
MPV: mean platelet volume
MR: Mendelian randomization
OR: odds ratio
PCT: plateletcrit
PDW: platelet distribution width
PIs: platelet indices
PLT: platelet count
RA: rheumatoid arthritis
RF: rheumatoid factor
SNPs: single nucleotide polymorphisms
TSMR: two‐sample Mendelian randomization
WM: weighted median

## Author Contributions

Xu‐Yan Shen and Shu‐Shan Zhao conceived the study, searched the literature, and performed data extraction. Xu‐Yan Shen conducted the primary statistical analysis, and Jia‐Ying Sun provided statistical expertise. Xu‐Yan Shen and Jia‐Ying Sun took the lead in writing the manuscript. Xin Wang contributed by critically revising the manuscript. Shu‐Shan Zhao provided clinical expertise. All authors provided critical feedback and helped shape the research, analysis, and manuscript. Xu‐Yan Shen, Jia‐Ying Sun, and Xin Wang contributed equally to this work and should be considered cofirst authors.

## Funding

This study was funded by grants from Zhejiang Provincial Medical and Health Science and Technology Project (2023RC291) and Shaoxing Municipal Medical and Health Science and Technology Plan Project (2024SKY043).

## Ethics Statement

Ethical approval was not required because this study used anonymous, open data.

## Consent

The authors have nothing to report.

## Conflicts of Interest

The authors declare no conflicts of interest.

## Supporting Information

Additional supporting information can be found online in the Supporting Information section.

## Supporting information


**Supporting Information 1** Figure S1: Statistical power of causal associations between four PIs and RA. Abbreviations: MPV, mean platelet volume; PCT, plateletcrit; PDW, platelet distribution width; PLT, platelet count.

**Supporting Information 2** Figure S2: The results of FDR correction for multiple tests. Abbreviations: FDR, false discovery rate; MPV, mean platelet volume; PCT, plateletcrit; PDW, platelet distribution width; PLT, platelet count.

**Supporting Information 3** Table S1: Single nucleotide polymorphisms (SNPs) associated with exposure and outcome. Table S2: Causal association between PCT and RA. Table S3: Causal association between PLT and RA. Table S4: Causal association between PDW and RA. Table S5: Causal association between MPV and RA. Table S6: Causal association between RA and PCT. Table S7: Causal association between RA and PLT. Table S8: Causal association between RA and PDW. Table S9: Causal association between RA and MPV.

## Data Availability

The data that support the findings of this study are available from the corresponding author upon reasonable request.
