# Supplementary figures and images for: Causal Interplay Between Platelet Indices and Rheumatoid Arthritis: Genetic Evidence From Bidirectional Mendelian Randomization
Source: Int J Genomics. 2026 Jul 30;2026:4549330. doi: 10.1155/ijog/4549330 (PMC13420736; doi:10.1155/ijog/4549330)

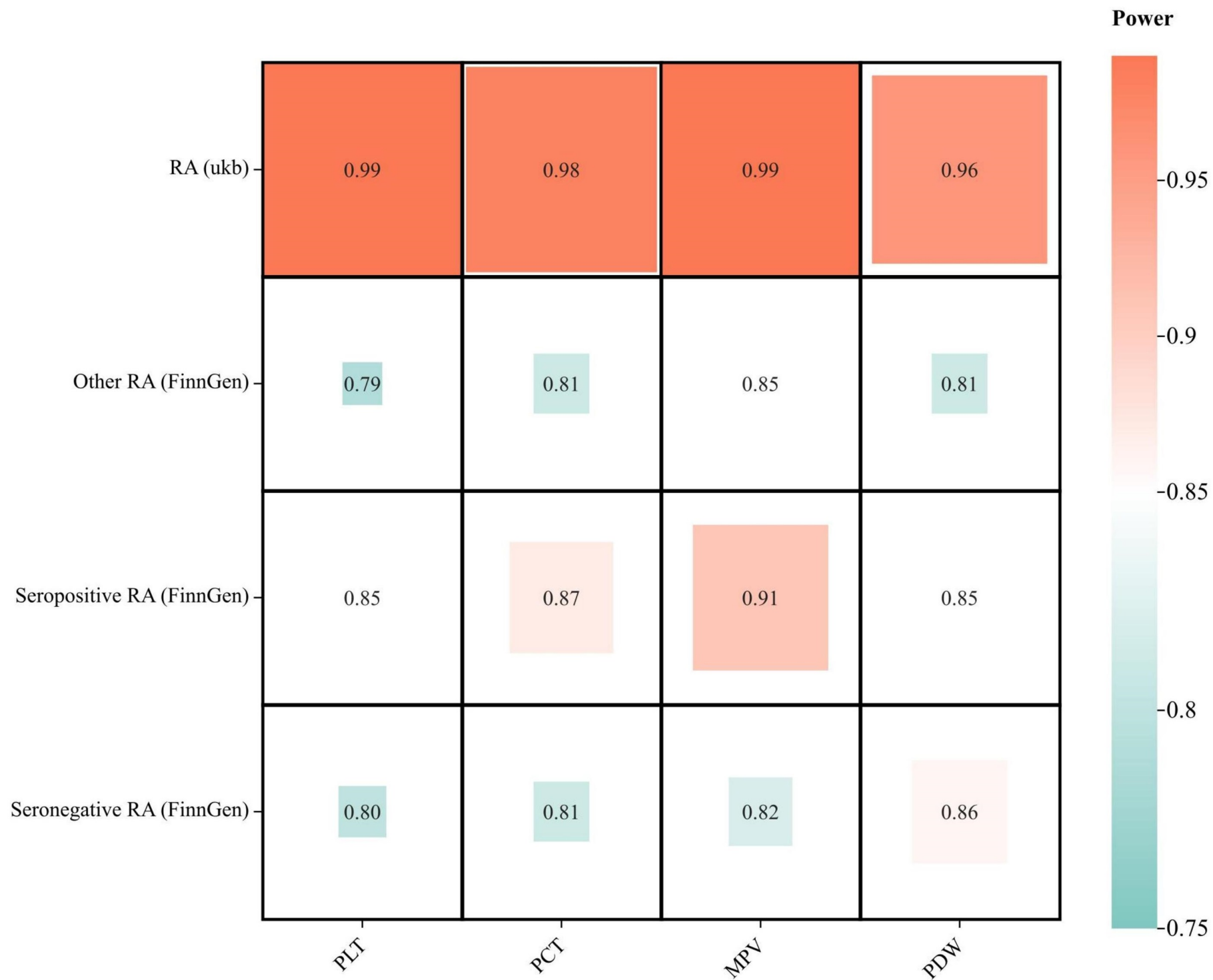

Supplement: Supplementary file 1 — Supporting Information 1 Figure S1: Statistical power of causal associations between four PIs and RA. Abbreviations: MPV, mean platelet volume; PCT, plateletcrit; PDW, platelet distribution width; PLT, platelet count. [file IJOG-2026-4549330-s003.pdf]

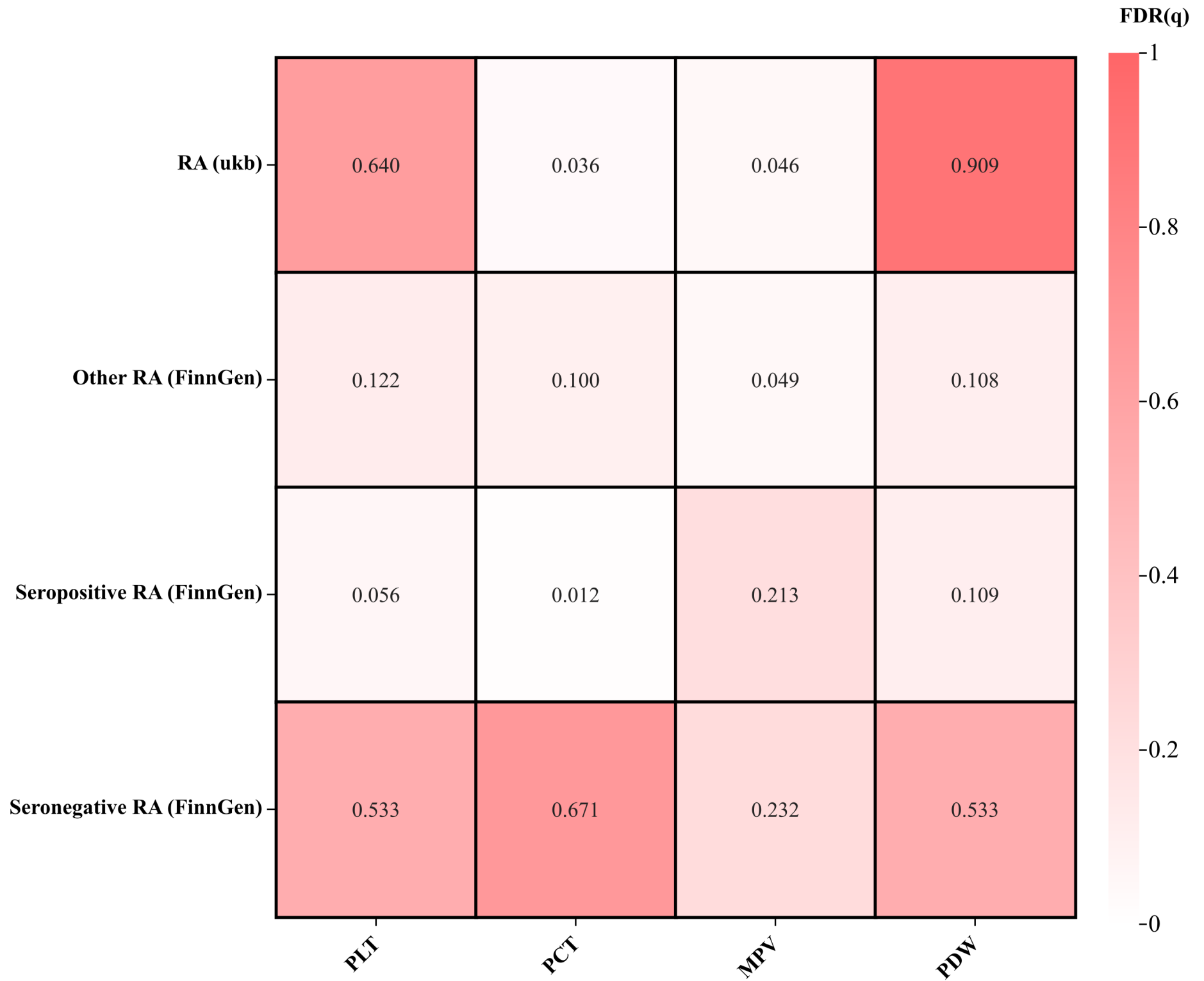

Supplement: Supplementary file 2 — Supporting Information 2 Figure S2: The results of FDR correction for multiple tests. Abbreviations: FDR, false discovery rate; MPV, mean platelet volume; PCT, plateletcrit; PDW, platelet distribution width; PLT, platelet count. [file IJOG-2026-4549330-s002.pdf]
