# Supplementary material for: Causal Interplay Between Platelet Indices and Rheumatoid Arthritis: Genetic Evidence From Bidirectional Mendelian Randomization
Source: Int J Genomics. 2026 Jul 30;2026:4549330. doi: 10.1155/ijog/4549330 (PMC13420736; doi:10.1155/ijog/4549330)
Supplement: Supplementary file 3 — Supporting Information 3 Table S1: Single nucleotide polymorphisms (SNPs) associated with exposure and outcome. Table S2: Causal association between PCT and RA. Table S3: Causal association between PLT and RA. Table S4: Causal association between PDW and RA. Table S5: Causal association between MPV and RA. Table S6: Causal association between RA and PCT. Table S7: Causal association between RA and PLT. Table S8: Causal association between RA and PDW. Table S9: Causal association between RA and MPV. [file IJOG-2026-4549330-s001.docx]

***Supplementary Materials***

**Supplementary Table S1 Single nucleotide polymorphisms (SNPs) associated with exposure and outcome**

| **Phenotypes** | **SNP** | **Chr** | **Position** | **Effect allele** | **Other allele** | **EAF** | **Effect** | **SE** | ***P* value** | **n** | **Variance explained (%)** | **F-statistic** |
| --- | --- | --- | --- | --- | --- | --- | --- | --- | --- | --- | --- | --- |
| Mean platelet (thrombocyte) volume | rs12041331 | 1 | 156869714 | A | G | 0.084 | -0.085 | 0.004 | 1.05E-85 | 350470 | 0.129 | 451.560 |
| Mean platelet (thrombocyte) volume | rs3924236 | 1 | 25895406 | T | C | 0.461 | -0.037 | 0.002 | 1.90E-53 | 350470 | 0.098 | 342.248 |
| Mean platelet (thrombocyte) volume | rs11121012 | 1 | 7794289 | G | A | 0.646 | -0.033 | 0.003 | 2.02E-39 | 350470 | 0.035 | 120.999 |
| Mean platelet (thrombocyte) volume | rs17020634 | 1 | 207273957 | G | A | 0.067 | 0.054 | 0.005 | 3.21E-29 | 350470 | 0.033 | 116.639 |
| Mean platelet (thrombocyte) volume | rs1168124 | 1 | 63139730 | T | C | 0.649 | 0.022 | 0.003 | 2.07E-18 | 350470 | 0.015 | 53.777 |
| Mean platelet (thrombocyte) volume | rs41299597 | 1 | 156882757 | G | C | 0.021 | 0.071 | 0.008 | 6.53E-18 | 350470 | 0.022 | 78.765 |
| Mean platelet (thrombocyte) volume | rs3767744 | 1 | 198651191 | G | C | 0.027 | 0.06 | 0.007 | 5.16E-16 | 350470 | 0.021 | 73.469 |
| Mean platelet (thrombocyte) volume | rs234108 | 1 | 184942671 | A | G | 0.478 | 0.015 | 0.002 | 7.19E-10 | 350470 | 0.016 | 56.250 |
| Mean platelet (thrombocyte) volume | rs1936933 | 1 | 114917230 | G | A | 0.105 | -0.023 | 0.004 | 7.66E-09 | 350470 | 0.009 | 33.062 |
| Mean platelet (thrombocyte) volume | rs2982505 | 1 | 40864877 | C | T | 0.85 | -0.019 | 0.003 | 7.91E-09 | 350470 | 0.011 | 40.111 |
| Mean platelet (thrombocyte) volume | rs10915885 | 1 | 226025288 | A | G | 0.2 | 0.017 | 0.003 | 1.24E-08 | 350470 | 0.009 | 32.111 |
| Mean platelet (thrombocyte) volume | rs1768584 | 1 | 205240145 | G | A | 0.386 | -0.118 | 0.002 | 1.00E-200 | 350470 | 0.983 | 3480.980 |
| Mean platelet (thrombocyte) volume | rs391360 | 1 | 29199676 | G | A | 0.067 | 0.07 | 0.005 | 3.09E-49 | 350470 | 0.056 | 195.999 |
| Mean platelet (thrombocyte) volume | rs7524494 | 1 | 113197800 | G | A | 0.526 | 0.029 | 0.002 | 2.17E-33 | 350470 | 0.060 | 210.249 |
| Mean platelet (thrombocyte) volume | rs116027976 | 1 | 172017226 | G | A | 0.02 | 0.077 | 0.009 | 1.05E-18 | 350470 | 0.021 | 73.197 |
| Mean platelet (thrombocyte) volume | rs496954 | 1 | 90229395 | A | T | 0.429 | 0.021 | 0.002 | 5.71E-18 | 350470 | 0.031 | 110.249 |
| Mean platelet (thrombocyte) volume | rs111631653 | 1 | 172417092 | A | T | 0.018 | 0.072 | 0.009 | 5.50E-15 | 350470 | 0.018 | 64.000 |
| Mean platelet (thrombocyte) volume | rs6695877 | 1 | 202845407 | G | A | 0.646 | -0.015 | 0.003 | 2.12E-09 | 350470 | 0.007 | 25.000 |
| Mean platelet (thrombocyte) volume | rs34746190 | 1 | 161225281 | A | G | 0.182 | -0.018 | 0.003 | 1.03E-08 | 350470 | 0.010 | 36.000 |
| Mean platelet (thrombocyte) volume | rs2038479 | 1 | 171939290 | A | C | 0.804 | 0.133 | 0.003 | 1.00E-200 | 350470 | 0.558 | 1965.433 |
| Mean platelet (thrombocyte) volume | rs4925750 | 1 | 248038325 | C | T | 0.311 | -0.075 | 0.003 | 9.86E-186 | 350470 | 0.178 | 624.996 |
| Mean platelet (thrombocyte) volume | rs11121529 | 1 | 10271688 | G | C | 0.114 | 0.082 | 0.004 | 3.76E-105 | 350470 | 0.120 | 420.248 |
| Mean platelet (thrombocyte) volume | rs7529794 | 1 | 40403370 | T | G | 0.265 | -0.026 | 0.003 | 6.70E-22 | 350470 | 0.021 | 75.111 |
| Mean platelet (thrombocyte) volume | rs16844819 | 1 | 224360933 | G | T | 0.441 | 0.018 | 0.002 | 4.62E-13 | 350470 | 0.023 | 81.000 |
| Mean platelet (thrombocyte) volume | rs2385054 | 1 | 236206864 | C | T | 0.433 | -0.016 | 0.002 | 6.25E-11 | 350470 | 0.018 | 64.000 |
| Mean platelet (thrombocyte) volume | rs72716340 | 1 | 90354229 | C | G | 0.143 | 0.021 | 0.003 | 1.04E-09 | 350470 | 0.014 | 49.000 |
| Mean platelet (thrombocyte) volume | rs12753585 | 1 | 248185951 | G | A | 0.035 | 0.043 | 0.007 | 1.31E-09 | 350470 | 0.011 | 37.734 |
| Mean platelet (thrombocyte) volume | rs41315846 | 1 | 247712303 | C | T | 0.477 | -0.061 | 0.002 | 7.55E-140 | 350470 | 0.265 | 930.245 |
| Mean platelet (thrombocyte) volume | rs2789422 | 1 | 159892088 | A | G | 0.417 | 0.033 | 0.002 | 5.90E-43 | 350470 | 0.078 | 272.248 |
| Mean platelet (thrombocyte) volume | rs3219484 | 1 | 45800156 | T | C | 0.075 | -0.047 | 0.005 | 2.78E-25 | 350470 | 0.025 | 88.359 |
| Mean platelet (thrombocyte) volume | rs72714915 | 1 | 161136343 | G | C | 0.082 | -0.045 | 0.004 | 1.36E-24 | 350470 | 0.036 | 126.562 |
| Mean platelet (thrombocyte) volume | rs61830291 | 1 | 221001142 | C | A | 0.097 | -0.034 | 0.004 | 7.60E-17 | 350470 | 0.021 | 72.250 |
| Mean platelet (thrombocyte) volume | rs2809948 | 1 | 52424094 | A | G | 0.296 | 0.021 | 0.003 | 1.14E-15 | 350470 | 0.014 | 49.000 |
| Mean platelet (thrombocyte) volume | rs12128561 | 1 | 17719645 | G | A | 0.657 | 0.015 | 0.003 | 1.15E-09 | 350470 | 0.007 | 25.000 |
| Mean platelet (thrombocyte) volume | rs76452111 | 1 | 171810155 | T | A | 0.043 | 0.099 | 0.006 | 1.97E-62 | 350470 | 0.078 | 272.248 |
| Mean platelet (thrombocyte) volume | rs2802839 | 1 | 204972288 | T | G | 0.63 | -0.039 | 0.003 | 1.20E-54 | 350470 | 0.048 | 168.999 |
| Mean platelet (thrombocyte) volume | rs3767809 | 1 | 118154831 | C | T | 0.221 | -0.043 | 0.003 | 2.28E-50 | 350470 | 0.059 | 205.443 |
| Mean platelet (thrombocyte) volume | rs72691294 | 1 | 111733884 | A | G | 0.063 | 0.06 | 0.005 | 4.89E-33 | 350470 | 0.041 | 143.999 |
| Mean platelet (thrombocyte) volume | rs1569419 | 1 | 2996602 | C | T | 0.767 | 0.032 | 0.003 | 9.99E-29 | 350470 | 0.032 | 113.777 |
| Mean platelet (thrombocyte) volume | rs142518196 | 1 | 248025390 | G | A | 0.028 | 0.069 | 0.007 | 9.31E-21 | 350470 | 0.028 | 97.163 |
| Mean platelet (thrombocyte) volume | rs17037388 | 1 | 11858036 | G | A | 0.164 | 0.021 | 0.003 | 6.56E-11 | 350470 | 0.014 | 49.000 |
| Mean platelet (thrombocyte) volume | rs11807162 | 1 | 243454680 | A | G | 0.115 | -0.021 | 0.004 | 3.88E-08 | 350470 | 0.008 | 27.562 |
| Mean platelet (thrombocyte) volume | rs655029 | 2 | 31477838 | A | G | 0.708 | -0.087 | 0.003 | 1.00E-200 | 350470 | 0.239 | 840.995 |
| Mean platelet (thrombocyte) volume | rs1861435 | 2 | 37075013 | A | T | 0.425 | -0.038 | 0.002 | 9.77E-55 | 350470 | 0.103 | 360.998 |
| Mean platelet (thrombocyte) volume | rs7584993 | 2 | 223845942 | A | C | 0.681 | 0.025 | 0.003 | 7.65E-22 | 350470 | 0.020 | 69.444 |
| Mean platelet (thrombocyte) volume | rs7589790 | 2 | 106433909 | G | C | 0.385 | -0.023 | 0.002 | 3.40E-21 | 350470 | 0.038 | 132.249 |
| Mean platelet (thrombocyte) volume | rs184885436 | 2 | 241263446 | T | C | 0.013 | -0.087 | 0.011 | 1.20E-15 | 350470 | 0.018 | 62.553 |
| Mean platelet (thrombocyte) volume | rs77687125 | 2 | 242652848 | T | C | 0.416 | -0.016 | 0.002 | 1.37E-10 | 350470 | 0.018 | 64.000 |
| Mean platelet (thrombocyte) volume | rs113919063 | 2 | 68874107 | A | G | 0.023 | -0.051 | 0.008 | 2.78E-10 | 350470 | 0.012 | 40.640 |
| Mean platelet (thrombocyte) volume | rs113885167 | 2 | 9485145 | T | A | 0.255 | 0.016 | 0.003 | 6.19E-09 | 350470 | 0.008 | 28.444 |
| Mean platelet (thrombocyte) volume | rs17572109 | 2 | 219093934 | A | G | 0.234 | -0.04 | 0.003 | 1.98E-45 | 350470 | 0.051 | 177.777 |
| Mean platelet (thrombocyte) volume | rs6759839 | 2 | 16620803 | G | A | 0.678 | -0.034 | 0.003 | 5.23E-40 | 350470 | 0.037 | 128.444 |
| Mean platelet (thrombocyte) volume | rs11125553 | 2 | 55186928 | T | C | 0.367 | 0.023 | 0.002 | 1.71E-20 | 350470 | 0.038 | 132.249 |
| Mean platelet (thrombocyte) volume | rs10865130 | 2 | 38046787 | G | C | 0.322 | 0.022 | 0.003 | 1.22E-17 | 350470 | 0.015 | 53.777 |
| Mean platelet (thrombocyte) volume | rs62175230 | 2 | 160677674 | T | C | 0.565 | -0.018 | 0.002 | 1.16E-13 | 350470 | 0.023 | 81.000 |
| Mean platelet (thrombocyte) volume | rs1047891 | 2 | 211540507 | A | C | 0.316 | 0.035 | 0.003 | 5.10E-43 | 350470 | 0.039 | 136.110 |
| Mean platelet (thrombocyte) volume | rs79716587 | 2 | 143886819 | A | G | 0.127 | 0.041 | 0.004 | 7.42E-30 | 350470 | 0.030 | 105.062 |
| Mean platelet (thrombocyte) volume | rs7605327 | 2 | 24035507 | T | C | 0.705 | 0.023 | 0.003 | 1.75E-18 | 350470 | 0.017 | 58.777 |
| Mean platelet (thrombocyte) volume | rs4972699 | 2 | 175455304 | C | G | 0.958 | 0.038 | 0.006 | 2.95E-10 | 350470 | 0.011 | 40.111 |
| Mean platelet (thrombocyte) volume | rs7349311 | 2 | 70165966 | A | G | 0.187 | 0.04 | 0.003 | 4.95E-38 | 350470 | 0.051 | 177.777 |
| Mean platelet (thrombocyte) volume | rs68066031 | 2 | 224880498 | C | T | 0.231 | -0.031 | 0.003 | 7.96E-28 | 350470 | 0.030 | 106.777 |
| Mean platelet (thrombocyte) volume | rs11681966 | 2 | 100759457 | A | C | 0.358 | 0.026 | 0.002 | 1.96E-26 | 350470 | 0.048 | 168.999 |
| Mean platelet (thrombocyte) volume | rs1860168 | 2 | 191724500 | A | G | 0.261 | -0.021 | 0.003 | 3.47E-15 | 350470 | 0.014 | 49.000 |
| Mean platelet (thrombocyte) volume | rs144333582 | 2 | 171837458 | A | G | 0.055 | -0.037 | 0.005 | 2.61E-12 | 350470 | 0.016 | 54.760 |
| Mean platelet (thrombocyte) volume | rs2679718 | 2 | 86130555 | T | C | 0.602 | 0.017 | 0.002 | 6.85E-12 | 350470 | 0.021 | 72.250 |
| Mean platelet (thrombocyte) volume | rs780094 | 2 | 27741237 | C | T | 0.618 | 0.016 | 0.002 | 6.77E-11 | 350470 | 0.018 | 64.000 |
| Mean platelet (thrombocyte) volume | rs1429271 | 2 | 100452674 | C | T | 0.885 | 0.023 | 0.004 | 1.26E-09 | 350470 | 0.009 | 33.062 |
| Mean platelet (thrombocyte) volume | rs72806653 | 2 | 54892663 | A | C | 0.203 | 0.017 | 0.003 | 5.20E-09 | 350470 | 0.009 | 32.111 |
| Mean platelet (thrombocyte) volume | rs78909033 | 2 | 241510903 | A | G | 0.135 | -0.177 | 0.003 | 1.00E-200 | 350470 | 0.983 | 3480.980 |
| Mean platelet (thrombocyte) volume | rs7585866 | 2 | 192696255 | G | A | 0.353 | -0.041 | 0.002 | 5.82E-61 | 350470 | 0.120 | 420.248 |
| Mean platelet (thrombocyte) volume | rs4849845 | 2 | 121013169 | A | G | 0.688 | 0.042 | 0.003 | 1.25E-58 | 350470 | 0.056 | 195.999 |
| Mean platelet (thrombocyte) volume | rs1519103 | 2 | 66677881 | T | G | 0.664 | -0.029 | 0.003 | 5.39E-30 | 350470 | 0.027 | 93.444 |
| Mean platelet (thrombocyte) volume | rs56017561 | 2 | 85658993 | C | T | 0.451 | 0.022 | 0.002 | 2.62E-19 | 350470 | 0.035 | 120.999 |
| Mean platelet (thrombocyte) volume | rs6745534 | 2 | 109314678 | A | G | 0.601 | 0.02 | 0.002 | 2.22E-16 | 350470 | 0.029 | 99.999 |
| Mean platelet (thrombocyte) volume | rs62194667 | 2 | 203688035 | G | C | 0.606 | -0.017 | 0.002 | 4.06E-12 | 350470 | 0.021 | 72.250 |
| Mean platelet (thrombocyte) volume | rs62105462 | 2 | 8707509 | C | T | 0.055 | -0.029 | 0.005 | 4.19E-08 | 350470 | 0.010 | 33.640 |
| Mean platelet (thrombocyte) volume | rs11712256 | 3 | 56733797 | T | C | 0.307 | -0.048 | 0.003 | 3.60E-77 | 350470 | 0.073 | 255.999 |
| Mean platelet (thrombocyte) volume | rs3804749 | 3 | 122833003 | T | C | 0.593 | 0.043 | 0.002 | 6.62E-71 | 350470 | 0.132 | 462.247 |
| Mean platelet (thrombocyte) volume | rs41433248 | 3 | 56962393 | C | T | 0.157 | -0.051 | 0.003 | 1.59E-53 | 350470 | 0.082 | 288.998 |
| Mean platelet (thrombocyte) volume | rs6805034 | 3 | 15856929 | G | C | 0.77 | -0.03 | 0.003 | 1.19E-25 | 350470 | 0.029 | 99.999 |
| Mean platelet (thrombocyte) volume | rs80217383 | 3 | 124041337 | G | A | 0.016 | -0.063 | 0.01 | 1.64E-10 | 350470 | 0.011 | 39.690 |
| Mean platelet (thrombocyte) volume | rs11128264 | 3 | 72392310 | T | C | 0.493 | -0.014 | 0.002 | 1.36E-09 | 350470 | 0.014 | 49.000 |
| Mean platelet (thrombocyte) volume | rs68055131 | 3 | 124347320 | T | C | 0.292 | 0.067 | 0.003 | 6.50E-142 | 350470 | 0.142 | 498.775 |
| Mean platelet (thrombocyte) volume | rs9836028 | 3 | 167383177 | C | G | 0.264 | -0.034 | 0.003 | 9.14E-37 | 350470 | 0.037 | 128.444 |
| Mean platelet (thrombocyte) volume | rs114008021 | 3 | 124186083 | A | T | 0.024 | -0.055 | 0.008 | 8.15E-12 | 350470 | 0.013 | 47.265 |
| Mean platelet (thrombocyte) volume | rs1354034 | 3 | 56849749 | C | T | 0.601 | -0.233 | 0.002 | 1.00E-200 | 350470 | 3.728 | 13572.173 |
| Mean platelet (thrombocyte) volume | rs67824942 | 3 | 55902009 | T | C | 0.097 | 0.026 | 0.004 | 6.67E-11 | 350470 | 0.012 | 42.250 |
| Mean platelet (thrombocyte) volume | rs143727980 | 3 | 124284021 | A | G | 0.021 | 0.056 | 0.009 | 8.27E-11 | 350470 | 0.011 | 38.716 |
| Mean platelet (thrombocyte) volume | rs2230169 | 3 | 47957996 | G | C | 0.073 | -0.028 | 0.005 | 1.70E-09 | 350470 | 0.009 | 31.360 |
| Mean platelet (thrombocyte) volume | rs2313211 | 3 | 183738626 | A | T | 0.554 | -0.023 | 0.002 | 6.93E-21 | 350470 | 0.038 | 132.249 |
| Mean platelet (thrombocyte) volume | rs6800015 | 3 | 178779546 | T | C | 0.738 | 0.023 | 0.003 | 4.00E-17 | 350470 | 0.017 | 58.777 |
| Mean platelet (thrombocyte) volume | rs12637288 | 3 | 196005444 | A | G | 0.479 | 0.019 | 0.002 | 6.04E-15 | 350470 | 0.026 | 90.249 |
| Mean platelet (thrombocyte) volume | rs2676362 | 3 | 107179527 | G | T | 0.727 | -0.017 | 0.003 | 7.45E-10 | 350470 | 0.009 | 32.111 |
| Mean platelet (thrombocyte) volume | rs9859042 | 3 | 98677386 | A | G | 0.052 | 0.031 | 0.005 | 4.95E-09 | 350470 | 0.011 | 38.440 |
| Mean platelet (thrombocyte) volume | rs11714268 | 3 | 124387045 | A | G | 0.025 | -0.155 | 0.008 | 1.78E-90 | 350470 | 0.107 | 375.388 |
| Mean platelet (thrombocyte) volume | rs7641175 | 3 | 18311412 | A | G | 0.783 | -0.036 | 0.003 | 3.59E-35 | 350470 | 0.041 | 143.999 |
| Mean platelet (thrombocyte) volume | rs2271494 | 3 | 113300183 | T | A | 0.417 | 0.023 | 0.002 | 4.26E-22 | 350470 | 0.038 | 132.249 |
| Mean platelet (thrombocyte) volume | rs16853231 | 3 | 168857253 | A | G | 0.074 | 0.041 | 0.005 | 3.20E-19 | 350470 | 0.019 | 67.240 |
| Mean platelet (thrombocyte) volume | rs9833022 | 3 | 136632122 | T | C | 0.016 | -0.058 | 0.009 | 8.70E-10 | 350470 | 0.012 | 41.531 |
| Mean platelet (thrombocyte) volume | rs16853746 | 3 | 169159136 | C | T | 0.045 | 0.033 | 0.006 | 1.14E-08 | 350470 | 0.009 | 30.250 |
| Mean platelet (thrombocyte) volume | rs62301688 | 4 | 48073073 | T | C | 0.273 | -0.018 | 0.003 | 2.67E-11 | 350470 | 0.010 | 36.000 |
| Mean platelet (thrombocyte) volume | rs62291089 | 4 | 6910335 | G | C | 0.195 | 0.034 | 0.003 | 3.77E-27 | 350470 | 0.037 | 128.444 |
| Mean platelet (thrombocyte) volume | rs2850379 | 4 | 102917419 | A | C | 0.435 | -0.021 | 0.002 | 4.11E-18 | 350470 | 0.031 | 110.249 |
| Mean platelet (thrombocyte) volume | rs143407796 | 4 | 79640858 | C | G | 0.258 | -0.019 | 0.003 | 5.10E-12 | 350470 | 0.011 | 40.111 |
| Mean platelet (thrombocyte) volume | rs11098323 | 4 | 79638158 | A | G | 0.623 | 0.016 | 0.002 | 2.45E-10 | 350470 | 0.018 | 64.000 |
| Mean platelet (thrombocyte) volume | rs62291983 | 4 | 7771397 | G | C | 0.302 | 0.016 | 0.003 | 1.61E-09 | 350470 | 0.008 | 28.444 |
| Mean platelet (thrombocyte) volume | rs2390352 | 4 | 123558330 | T | C | 0.952 | -0.031 | 0.006 | 4.92E-08 | 350470 | 0.008 | 26.694 |
| Mean platelet (thrombocyte) volume | rs11734099 | 4 | 6891435 | A | G | 0.176 | -0.104 | 0.003 | 1.00E-200 | 350470 | 0.342 | 1201.771 |
| Mean platelet (thrombocyte) volume | rs3733377 | 4 | 88029482 | G | C | 0.212 | -0.027 | 0.003 | 4.55E-20 | 350470 | 0.023 | 81.000 |
| Mean platelet (thrombocyte) volume | rs12650244 | 4 | 6755033 | T | C | 0.121 | 0.028 | 0.004 | 7.00E-14 | 350470 | 0.014 | 49.000 |
| Mean platelet (thrombocyte) volume | rs28396651 | 4 | 102819025 | G | A | 0.601 | 0.033 | 0.002 | 3.93E-42 | 350470 | 0.078 | 272.248 |
| Mean platelet (thrombocyte) volume | rs12643081 | 4 | 106019837 | C | T | 0.687 | -0.025 | 0.003 | 1.25E-21 | 350470 | 0.020 | 69.444 |
| Mean platelet (thrombocyte) volume | rs45608936 | 4 | 123192240 | C | A | 0.02 | -0.077 | 0.008 | 1.19E-19 | 350470 | 0.026 | 92.640 |
| Mean platelet (thrombocyte) volume | rs3816086 | 4 | 154418561 | C | T | 0.454 | -0.017 | 0.002 | 3.20E-13 | 350470 | 0.021 | 72.250 |
| Mean platelet (thrombocyte) volume | rs12645934 | 4 | 154457328 | A | G | 0.032 | -0.073 | 0.007 | 1.04E-26 | 350470 | 0.031 | 108.754 |
| Mean platelet (thrombocyte) volume | rs362312 | 4 | 3237644 | C | T | 0.426 | 0.023 | 0.002 | 4.53E-21 | 350470 | 0.038 | 132.249 |
| Mean platelet (thrombocyte) volume | rs3796529 | 4 | 57797414 | T | C | 0.185 | 0.028 | 0.003 | 4.70E-20 | 350470 | 0.025 | 87.111 |
| Mean platelet (thrombocyte) volume | rs28681431 | 4 | 38687928 | A | G | 0.215 | 0.024 | 0.003 | 1.68E-16 | 350470 | 0.018 | 64.000 |
| Mean platelet (thrombocyte) volume | rs72665823 | 4 | 89833082 | G | A | 0.16 | -0.025 | 0.003 | 4.10E-14 | 350470 | 0.020 | 69.444 |
| Mean platelet (thrombocyte) volume | rs699861 | 4 | 95420163 | A | G | 0.809 | 0.022 | 0.003 | 8.52E-13 | 350470 | 0.015 | 53.777 |
| Mean platelet (thrombocyte) volume | rs6556405 | 5 | 158635102 | C | T | 0.249 | 0.07 | 0.003 | 9.44E-141 | 350470 | 0.155 | 544.441 |
| Mean platelet (thrombocyte) volume | rs7726369 | 5 | 158227691 | C | G | 0.084 | 0.034 | 0.004 | 5.02E-15 | 350470 | 0.021 | 72.250 |
| Mean platelet (thrombocyte) volume | rs895376 | 5 | 66033805 | A | G | 0.355 | 0.018 | 0.003 | 1.44E-12 | 350470 | 0.010 | 36.000 |
| Mean platelet (thrombocyte) volume | rs34592828 | 5 | 75996909 | A | G | 0.046 | 0.277 | 0.006 | 1.00E-200 | 350470 | 0.604 | 2131.349 |
| Mean platelet (thrombocyte) volume | rs2631360 | 5 | 131707429 | A | G | 0.519 | -0.045 | 0.002 | 6.12E-78 | 350470 | 0.144 | 506.247 |
| Mean platelet (thrombocyte) volume | rs1476096 | 5 | 133837021 | C | A | 0.58 | -0.025 | 0.002 | 2.39E-25 | 350470 | 0.045 | 156.249 |
| Mean platelet (thrombocyte) volume | rs75144132 | 5 | 75974555 | A | C | 0.016 | 0.098 | 0.01 | 7.93E-23 | 350470 | 0.027 | 96.039 |
| Mean platelet (thrombocyte) volume | rs7712401 | 5 | 122374459 | T | C | 0.537 | 0.023 | 0.002 | 4.10E-22 | 350470 | 0.038 | 132.249 |
| Mean platelet (thrombocyte) volume | rs72648830 | 5 | 177608542 | T | C | 0.11 | -0.03 | 0.004 | 4.03E-15 | 350470 | 0.016 | 56.250 |
| Mean platelet (thrombocyte) volume | rs62332588 | 5 | 1289880 | C | T | 0.602 | -0.019 | 0.003 | 6.66E-14 | 350470 | 0.011 | 40.111 |
| Mean platelet (thrombocyte) volume | rs6452819 | 5 | 88636854 | A | G | 0.025 | 0.05 | 0.008 | 6.75E-11 | 350470 | 0.011 | 39.062 |
| Mean platelet (thrombocyte) volume | rs62367623 | 5 | 43614589 | G | A | 0.055 | 0.031 | 0.005 | 4.56E-09 | 350470 | 0.011 | 38.440 |
| Mean platelet (thrombocyte) volume | rs35188965 | 5 | 1104938 | T | C | 0.582 | -0.037 | 0.002 | 7.50E-53 | 350470 | 0.098 | 342.248 |
| Mean platelet (thrombocyte) volume | rs56116444 | 5 | 111061847 | G | T | 0.075 | -0.029 | 0.005 | 1.39E-10 | 350470 | 0.010 | 33.640 |
| Mean platelet (thrombocyte) volume | rs6889730 | 5 | 60121097 | G | A | 0.816 | -0.052 | 0.003 | 9.45E-65 | 350470 | 0.086 | 300.443 |
| Mean platelet (thrombocyte) volume | rs7722711 | 5 | 75906851 | C | T | 0.064 | -0.069 | 0.005 | 2.51E-46 | 350470 | 0.054 | 190.439 |
| Mean platelet (thrombocyte) volume | rs3212574 | 5 | 52366779 | A | G | 0.223 | -0.021 | 0.003 | 1.25E-13 | 350470 | 0.014 | 49.000 |
| Mean platelet (thrombocyte) volume | rs114694170 | 5 | 88180196 | C | T | 0.059 | -0.149 | 0.005 | 2.28E-188 | 350470 | 0.253 | 888.035 |
| Mean platelet (thrombocyte) volume | rs4538653 | 5 | 159595894 | C | G | 0.702 | 0.049 | 0.003 | 2.42E-79 | 350470 | 0.076 | 266.776 |
| Mean platelet (thrombocyte) volume | rs72761071 | 5 | 76056131 | G | C | 0.022 | 0.139 | 0.008 | 5.71E-65 | 350470 | 0.086 | 301.889 |
| Mean platelet (thrombocyte) volume | rs13355951 | 5 | 61516534 | G | C | 0.419 | -0.026 | 0.002 | 1.52E-27 | 350470 | 0.048 | 168.999 |
| Mean platelet (thrombocyte) volume | rs12717895 | 5 | 140965467 | T | A | 0.318 | -0.019 | 0.003 | 3.23E-14 | 350470 | 0.011 | 40.111 |
| Mean platelet (thrombocyte) volume | rs6866671 | 5 | 159436823 | C | A | 0.133 | 0.024 | 0.004 | 1.00E-11 | 350470 | 0.010 | 36.000 |
| Mean platelet (thrombocyte) volume | rs875742 | 5 | 173287763 | A | G | 0.406 | 0.015 | 0.002 | 1.25E-09 | 350470 | 0.016 | 56.250 |
| Mean platelet (thrombocyte) volume | rs2516471 | 6 | 31400292 | A | G | 0.063 | -0.125 | 0.005 | 6.46E-141 | 350470 | 0.178 | 624.996 |
| Mean platelet (thrombocyte) volume | rs214057 | 6 | 25531133 | C | T | 0.428 | 0.048 | 0.002 | 3.66E-87 | 350470 | 0.164 | 575.997 |
| Mean platelet (thrombocyte) volume | rs4711890 | 6 | 47606712 | G | C | 0.267 | -0.04 | 0.003 | 1.84E-50 | 350470 | 0.051 | 177.777 |
| Mean platelet (thrombocyte) volume | rs115550566 | 6 | 31183907 | G | A | 0.024 | -0.066 | 0.008 | 5.34E-17 | 350470 | 0.019 | 68.062 |
| Mean platelet (thrombocyte) volume | rs17057622 | 6 | 130014988 | C | A | 0.037 | 0.041 | 0.006 | 1.48E-10 | 350470 | 0.013 | 46.694 |
| Mean platelet (thrombocyte) volume | rs211510 | 6 | 11593985 | G | A | 0.466 | 0.015 | 0.002 | 1.37E-09 | 350470 | 0.016 | 56.250 |
| Mean platelet (thrombocyte) volume | rs17136561 | 6 | 3439697 | G | T | 0.191 | 0.032 | 0.003 | 2.10E-25 | 350470 | 0.032 | 113.777 |
| Mean platelet (thrombocyte) volume | rs688181 | 6 | 158092638 | C | T | 0.719 | 0.022 | 0.003 | 4.45E-16 | 350470 | 0.015 | 53.777 |
| Mean platelet (thrombocyte) volume | rs45575537 | 6 | 31869154 | G | C | 0.043 | 0.092 | 0.006 | 4.70E-56 | 350470 | 0.067 | 235.110 |
| Mean platelet (thrombocyte) volume | rs2057149 | 6 | 110717493 | T | C | 0.649 | -0.038 | 0.003 | 2.10E-51 | 350470 | 0.046 | 160.444 |
| Mean platelet (thrombocyte) volume | rs28780086 | 6 | 30105154 | G | A | 0.023 | -0.11 | 0.008 | 3.59E-44 | 350470 | 0.054 | 189.061 |
| Mean platelet (thrombocyte) volume | rs2691475 | 6 | 71326034 | A | G | 0.366 | 0.022 | 0.003 | 2.51E-18 | 350470 | 0.015 | 53.777 |
| Mean platelet (thrombocyte) volume | rs9379419 | 6 | 22552547 | A | G | 0.168 | 0.023 | 0.003 | 1.05E-12 | 350470 | 0.017 | 58.777 |
| Mean platelet (thrombocyte) volume | rs2800446 | 6 | 157801040 | A | T | 0.835 | -0.02 | 0.003 | 6.24E-10 | 350470 | 0.013 | 44.444 |
| Mean platelet (thrombocyte) volume | rs1630961 | 6 | 147510466 | C | A | 0.6 | -0.014 | 0.002 | 8.46E-09 | 350470 | 0.014 | 49.000 |
| Mean platelet (thrombocyte) volume | rs4236051 | 6 | 36469821 | T | C | 0.009 | -0.236 | 0.013 | 1.50E-76 | 350470 | 0.094 | 329.560 |
| Mean platelet (thrombocyte) volume | rs3180068 | 6 | 52269685 | A | C | 0.109 | -0.033 | 0.004 | 5.55E-18 | 350470 | 0.019 | 68.062 |
| Mean platelet (thrombocyte) volume | rs28399993 | 6 | 31732155 | A | G | 0.012 | 0.08 | 0.011 | 4.57E-13 | 350470 | 0.015 | 52.892 |
| Mean platelet (thrombocyte) volume | rs76173020 | 6 | 146649257 | A | T | 0.035 | -0.04 | 0.006 | 6.76E-10 | 350470 | 0.013 | 44.444 |
| Mean platelet (thrombocyte) volume | rs72860306 | 6 | 31232401 | T | C | 0.012 | -0.068 | 0.011 | 9.00E-10 | 350470 | 0.011 | 38.215 |
| Mean platelet (thrombocyte) volume | rs9472903 | 6 | 46882904 | G | A | 0.137 | -0.032 | 0.003 | 7.93E-21 | 350470 | 0.032 | 113.777 |
| Mean platelet (thrombocyte) volume | rs114000233 | 6 | 119256332 | C | A | 0.312 | 0.022 | 0.003 | 6.08E-17 | 350470 | 0.015 | 53.777 |
| Mean platelet (thrombocyte) volume | rs62428833 | 6 | 143263270 | T | C | 0.213 | -0.022 | 0.003 | 1.71E-14 | 350470 | 0.015 | 53.777 |
| Mean platelet (thrombocyte) volume | rs7755223 | 6 | 44055333 | G | A | 0.202 | -0.019 | 0.003 | 5.89E-10 | 350470 | 0.011 | 40.111 |
| Mean platelet (thrombocyte) volume | rs7773815 | 6 | 109602343 | C | A | 0.524 | -0.014 | 0.002 | 3.33E-09 | 350470 | 0.014 | 49.000 |
| Mean platelet (thrombocyte) volume | rs73186227 | 7 | 106409419 | A | G | 0.08 | -0.047 | 0.004 | 1.31E-26 | 350470 | 0.039 | 138.062 |
| Mean platelet (thrombocyte) volume | rs4731120 | 7 | 123411223 | C | A | 0.081 | -0.047 | 0.004 | 1.77E-26 | 350470 | 0.039 | 138.062 |
| Mean platelet (thrombocyte) volume | rs7385935 | 7 | 50430871 | A | G | 0.373 | 0.016 | 0.002 | 1.39E-10 | 350470 | 0.018 | 64.000 |
| Mean platelet (thrombocyte) volume | rs55747707 | 7 | 73037366 | A | G | 0.204 | 0.018 | 0.003 | 7.89E-10 | 350470 | 0.010 | 36.000 |
| Mean platelet (thrombocyte) volume | rs2527207 | 7 | 158743078 | G | A | 0.803 | 0.018 | 0.003 | 3.47E-09 | 350470 | 0.010 | 36.000 |
| Mean platelet (thrombocyte) volume | rs2966431 | 7 | 5552436 | A | G | 0.114 | -0.022 | 0.004 | 5.68E-09 | 350470 | 0.009 | 30.250 |
| Mean platelet (thrombocyte) volume | rs7808005 | 7 | 37457359 | T | C | 0.279 | 0.029 | 0.003 | 4.96E-28 | 350470 | 0.027 | 93.444 |
| Mean platelet (thrombocyte) volume | rs10258803 | 7 | 106222328 | C | T | 0.1 | -0.04 | 0.004 | 6.56E-24 | 350470 | 0.029 | 99.999 |
| Mean platelet (thrombocyte) volume | rs6959832 | 7 | 135289854 | A | G | 0.508 | -0.022 | 0.002 | 1.01E-19 | 350470 | 0.035 | 120.999 |
| Mean platelet (thrombocyte) volume | rs342293 | 7 | 106372219 | G | C | 0.459 | 0.17 | 0.002 | 1.00E-200 | 350470 | 2.020 | 7224.959 |
| Mean platelet (thrombocyte) volume | rs6796 | 7 | 6502367 | C | T | 0.278 | -0.035 | 0.003 | 1.73E-39 | 350470 | 0.039 | 136.110 |
| Mean platelet (thrombocyte) volume | rs2788503 | 7 | 158590269 | G | A | 0.885 | 0.049 | 0.004 | 1.82E-39 | 350470 | 0.043 | 150.062 |
| Mean platelet (thrombocyte) volume | rs757694 | 7 | 44932538 | A | C | 0.437 | -0.02 | 0.002 | 4.46E-16 | 350470 | 0.029 | 99.999 |
| Mean platelet (thrombocyte) volume | rs56287412 | 7 | 17978828 | C | T | 0.51 | 0.017 | 0.002 | 1.88E-12 | 350470 | 0.021 | 72.250 |
| Mean platelet (thrombocyte) volume | rs896156 | 7 | 129214418 | C | T | 0.913 | 0.029 | 0.004 | 1.27E-11 | 350470 | 0.015 | 52.562 |
| Mean platelet (thrombocyte) volume | rs55813553 | 7 | 101686215 | C | T | 0.203 | 0.017 | 0.003 | 2.99E-08 | 350470 | 0.009 | 32.111 |
| Mean platelet (thrombocyte) volume | rs818586 | 7 | 16693122 | C | G | 0.537 | -0.025 | 0.002 | 1.54E-25 | 350470 | 0.045 | 156.249 |
| Mean platelet (thrombocyte) volume | rs59882870 | 7 | 75638421 | A | G | 0.164 | -0.033 | 0.003 | 1.38E-24 | 350470 | 0.035 | 120.999 |
| Mean platelet (thrombocyte) volume | rs4729607 | 7 | 100326744 | A | T | 0.247 | 0.028 | 0.003 | 2.13E-23 | 350470 | 0.025 | 87.111 |
| Mean platelet (thrombocyte) volume | rs12706122 | 7 | 116567732 | A | G | 0.243 | 0.03 | 0.003 | 3.98E-26 | 350470 | 0.029 | 99.999 |
| Mean platelet (thrombocyte) volume | rs13233747 | 7 | 73149302 | A | G | 0.347 | -0.022 | 0.003 | 5.33E-19 | 350470 | 0.015 | 53.777 |
| Mean platelet (thrombocyte) volume | rs2188420 | 7 | 106486243 | G | C | 0.121 | -0.026 | 0.004 | 1.12E-12 | 350470 | 0.012 | 42.250 |
| Mean platelet (thrombocyte) volume | rs3218517 | 7 | 152350022 | A | C | 0.035 | -0.046 | 0.007 | 2.64E-12 | 350470 | 0.012 | 43.183 |
| Mean platelet (thrombocyte) volume | rs10107388 | 8 | 145004944 | C | T | 0.368 | -0.033 | 0.002 | 2.19E-39 | 350470 | 0.078 | 272.248 |
| Mean platelet (thrombocyte) volume | rs13259933 | 8 | 56749584 | A | G | 0.507 | 0.031 | 0.002 | 4.02E-39 | 350470 | 0.069 | 240.249 |
| Mean platelet (thrombocyte) volume | rs2257081 | 8 | 25225804 | T | C | 0.412 | 0.02 | 0.002 | 1.21E-16 | 350470 | 0.029 | 99.999 |
| Mean platelet (thrombocyte) volume | rs2721972 | 8 | 101478881 | C | T | 0.839 | -0.022 | 0.003 | 8.56E-12 | 350470 | 0.015 | 53.777 |
| Mean platelet (thrombocyte) volume | rs56094005 | 8 | 21769432 | G | A | 0.044 | 0.065 | 0.006 | 4.17E-29 | 350470 | 0.033 | 117.360 |
| Mean platelet (thrombocyte) volume | rs13257521 | 8 | 9031995 | G | C | 0.21 | 0.024 | 0.003 | 1.05E-15 | 350470 | 0.018 | 64.000 |
| Mean platelet (thrombocyte) volume | rs9693128 | 8 | 82706128 | G | A | 0.609 | -0.018 | 0.002 | 2.20E-13 | 350470 | 0.023 | 81.000 |
| Mean platelet (thrombocyte) volume | rs7814758 | 8 | 23372914 | C | G | 0.382 | -0.018 | 0.002 | 5.54E-13 | 350470 | 0.023 | 81.000 |
| Mean platelet (thrombocyte) volume | rs6993770 | 8 | 106581528 | T | A | 0.286 | 0.046 | 0.003 | 3.24E-67 | 350470 | 0.067 | 235.110 |
| Mean platelet (thrombocyte) volume | rs7008182 | 8 | 131344745 | A | T | 0.49 | 0.041 | 0.002 | 4.58E-65 | 350470 | 0.120 | 420.248 |
| Mean platelet (thrombocyte) volume | rs4921998 | 8 | 19013570 | T | A | 0.695 | -0.027 | 0.003 | 1.82E-25 | 350470 | 0.023 | 81.000 |
| Mean platelet (thrombocyte) volume | rs59893263 | 8 | 81401331 | T | A | 0.239 | -0.017 | 0.003 | 4.94E-10 | 350470 | 0.009 | 32.111 |
| Mean platelet (thrombocyte) volume | rs34650936 | 8 | 142238339 | A | C | 0.185 | -0.019 | 0.003 | 1.34E-09 | 350470 | 0.011 | 40.111 |
| Mean platelet (thrombocyte) volume | rs11775560 | 8 | 61660163 | G | A | 0.187 | 0.018 | 0.003 | 2.31E-09 | 350470 | 0.010 | 36.000 |
| Mean platelet (thrombocyte) volume | rs62506682 | 8 | 38714130 | A | C | 0.108 | -0.022 | 0.004 | 1.60E-08 | 350470 | 0.009 | 30.250 |
| Mean platelet (thrombocyte) volume | rs60757417 | 9 | 135864436 | G | C | 0.06 | 0.115 | 0.005 | 4.30E-115 | 350470 | 0.151 | 528.997 |
| Mean platelet (thrombocyte) volume | rs10820606 | 9 | 99192919 | C | A | 0.228 | -0.035 | 0.003 | 3.64E-33 | 350470 | 0.039 | 136.110 |
| Mean platelet (thrombocyte) volume | rs61751937 | 9 | 113312231 | C | G | 0.029 | 0.057 | 0.007 | 6.26E-16 | 350470 | 0.019 | 66.306 |
| Mean platelet (thrombocyte) volume | rs4837794 | 9 | 123507855 | C | T | 0.668 | -0.015 | 0.003 | 5.61E-09 | 350470 | 0.007 | 25.000 |
| Mean platelet (thrombocyte) volume | rs739844 | 9 | 35072001 | C | G | 0.717 | -0.015 | 0.003 | 1.14E-08 | 350470 | 0.007 | 25.000 |
| Mean platelet (thrombocyte) volume | rs11789898 | 9 | 136925663 | T | G | 0.166 | -0.053 | 0.003 | 1.65E-61 | 350470 | 0.089 | 312.109 |
| Mean platelet (thrombocyte) volume | rs114968084 | 9 | 136022954 | A | G | 0.03 | 0.1 | 0.007 | 3.33E-43 | 350470 | 0.058 | 204.080 |
| Mean platelet (thrombocyte) volume | rs10970977 | 9 | 32433961 | T | C | 0.572 | 0.014 | 0.002 | 1.56E-08 | 350470 | 0.014 | 49.000 |
| Mean platelet (thrombocyte) volume | rs10813766 | 9 | 331490 | G | T | 0.662 | -0.057 | 0.003 | 8.75E-115 | 350470 | 0.103 | 360.998 |
| Mean platelet (thrombocyte) volume | rs10761209 | 9 | 96077465 | C | A | 0.296 | -0.015 | 0.003 | 4.26E-09 | 350470 | 0.007 | 25.000 |
| Mean platelet (thrombocyte) volume | rs10970979 | 9 | 334337 | G | A | 0.306 | 0.046 | 0.003 | 3.30E-70 | 350470 | 0.067 | 235.110 |
| Mean platelet (thrombocyte) volume | rs76026826 | 9 | 135431419 | A | G | 0.017 | 0.11 | 0.01 | 1.91E-30 | 350470 | 0.035 | 120.999 |
| Mean platelet (thrombocyte) volume | rs114665548 | 9 | 73057291 | T | A | 0.433 | 0.028 | 0.002 | 1.98E-30 | 350470 | 0.056 | 195.999 |
| Mean platelet (thrombocyte) volume | rs62531962 | 9 | 163788 | G | A | 0.037 | -0.039 | 0.007 | 6.70E-09 | 350470 | 0.009 | 31.041 |
| Mean platelet (thrombocyte) volume | rs7045844 | 9 | 19053114 | T | G | 0.388 | -0.019 | 0.002 | 2.90E-15 | 350470 | 0.026 | 90.249 |
| Mean platelet (thrombocyte) volume | rs1905765 | 9 | 4650575 | G | T | 0.447 | -0.015 | 0.002 | 9.83E-10 | 350470 | 0.016 | 56.250 |
| Mean platelet (thrombocyte) volume | rs62550974 | 9 | 91406124 | A | C | 0.063 | -0.027 | 0.005 | 3.26E-08 | 350470 | 0.008 | 29.160 |
| Mean platelet (thrombocyte) volume | rs7090111 | 10 | 65077994 | G | C | 0.417 | -0.126 | 0.002 | 1.00E-200 | 350470 | 1.120 | 3968.977 |
| Mean platelet (thrombocyte) volume | rs11245346 | 10 | 126425537 | C | G | 0.162 | 0.024 | 0.003 | 1.67E-13 | 350470 | 0.018 | 64.000 |
| Mean platelet (thrombocyte) volume | rs2671540 | 10 | 65732498 | G | T | 0.667 | 0.015 | 0.003 | 1.11E-08 | 350470 | 0.007 | 25.000 |
| Mean platelet (thrombocyte) volume | rs603424 | 10 | 102075479 | A | G | 0.168 | -0.064 | 0.003 | 1.23E-89 | 350470 | 0.130 | 455.109 |
| Mean platelet (thrombocyte) volume | rs11257361 | 10 | 11895507 | T | A | 0.204 | -0.037 | 0.003 | 2.81E-36 | 350470 | 0.043 | 152.110 |
| Mean platelet (thrombocyte) volume | rs993284 | 10 | 11210860 | G | C | 0.195 | 0.019 | 0.003 | 1.48E-10 | 350470 | 0.011 | 40.111 |
| Mean platelet (thrombocyte) volume | rs1328599 | 10 | 97053775 | T | G | 0.924 | 0.048 | 0.005 | 5.08E-26 | 350470 | 0.026 | 92.159 |
| Mean platelet (thrombocyte) volume | rs872398 | 10 | 3135699 | T | C | 0.352 | -0.017 | 0.003 | 4.36E-12 | 350470 | 0.009 | 32.111 |
| Mean platelet (thrombocyte) volume | rs11190583 | 10 | 102252015 | G | C | 0.225 | 0.018 | 0.003 | 4.22E-10 | 350470 | 0.010 | 36.000 |
| Mean platelet (thrombocyte) volume | rs807027 | 10 | 102764607 | A | T | 0.248 | -0.017 | 0.003 | 4.74E-10 | 350470 | 0.009 | 32.111 |
| Mean platelet (thrombocyte) volume | rs1981150 | 10 | 89854115 | T | C | 0.116 | -0.031 | 0.004 | 2.54E-16 | 350470 | 0.017 | 60.062 |
| Mean platelet (thrombocyte) volume | rs11186852 | 10 | 93962124 | C | T | 0.279 | 0.017 | 0.003 | 1.19E-10 | 350470 | 0.009 | 32.111 |
| Mean platelet (thrombocyte) volume | rs10824305 | 10 | 77140469 | G | C | 0.581 | 0.014 | 0.002 | 2.58E-08 | 350470 | 0.014 | 49.000 |
| Mean platelet (thrombocyte) volume | rs118033845 | 10 | 11836092 | A | G | 0.035 | 0.068 | 0.007 | 6.40E-25 | 350470 | 0.027 | 94.367 |
| Mean platelet (thrombocyte) volume | rs1250600 | 10 | 81010850 | G | T | 0.648 | 0.018 | 0.002 | 2.76E-13 | 350470 | 0.023 | 81.000 |
| Mean platelet (thrombocyte) volume | rs3824949 | 11 | 5701409 | C | G | 0.558 | 0.033 | 0.002 | 9.99E-42 | 350470 | 0.078 | 272.248 |
| Mean platelet (thrombocyte) volume | rs10750866 | 11 | 57404779 | G | A | 0.27 | -0.03 | 0.003 | 5.22E-28 | 350470 | 0.029 | 99.999 |
| Mean platelet (thrombocyte) volume | rs2071304 | 11 | 47372377 | C | G | 0.318 | 0.018 | 0.003 | 4.92E-12 | 350470 | 0.010 | 36.000 |
| Mean platelet (thrombocyte) volume | rs55709369 | 11 | 48116535 | A | G | 0.047 | 0.035 | 0.006 | 3.67E-10 | 350470 | 0.010 | 34.028 |
| Mean platelet (thrombocyte) volume | rs640221 | 11 | 94832645 | G | A | 0.834 | -0.043 | 0.003 | 1.23E-40 | 350470 | 0.059 | 205.443 |
| Mean platelet (thrombocyte) volume | rs4910165 | 11 | 10674044 | G | C | 0.689 | -0.032 | 0.003 | 1.48E-34 | 350470 | 0.032 | 113.777 |
| Mean platelet (thrombocyte) volume | rs61744384 | 11 | 65387378 | A | T | 0.439 | -0.024 | 0.002 | 1.48E-23 | 350470 | 0.041 | 143.999 |
| Mean platelet (thrombocyte) volume | rs11603461 | 11 | 17056587 | A | T | 0.173 | 0.022 | 0.003 | 1.95E-12 | 350470 | 0.015 | 53.777 |
| Mean platelet (thrombocyte) volume | rs56305604 | 11 | 4045995 | A | G | 0.046 | 0.034 | 0.006 | 2.09E-09 | 350470 | 0.009 | 32.111 |
| Mean platelet (thrombocyte) volume | rs174559 | 11 | 61581656 | A | G | 0.282 | -0.051 | 0.003 | 1.40E-83 | 350470 | 0.082 | 288.998 |
| Mean platelet (thrombocyte) volume | rs10898864 | 11 | 72405450 | T | C | 0.27 | 0.029 | 0.003 | 2.74E-27 | 350470 | 0.027 | 93.444 |
| Mean platelet (thrombocyte) volume | rs680210 | 11 | 117172820 | C | T | 0.62 | 0.026 | 0.002 | 2.01E-25 | 350470 | 0.048 | 168.999 |
| Mean platelet (thrombocyte) volume | rs6762 | 11 | 838722 | C | T | 0.248 | 0.026 | 0.003 | 1.52E-21 | 350470 | 0.021 | 75.111 |
| Mean platelet (thrombocyte) volume | rs73584250 | 11 | 129145619 | T | C | 0.317 | -0.02 | 0.003 | 2.08E-14 | 350470 | 0.013 | 44.444 |
| Mean platelet (thrombocyte) volume | rs511141 | 11 | 95884017 | A | G | 0.344 | -0.015 | 0.003 | 1.84E-09 | 350470 | 0.007 | 25.000 |
| Mean platelet (thrombocyte) volume | rs75412970 | 11 | 57842836 | C | G | 0.025 | -0.042 | 0.008 | 3.00E-08 | 350470 | 0.008 | 27.562 |
| Mean platelet (thrombocyte) volume | rs11604127 | 11 | 196944 | T | C | 0.232 | -0.12 | 0.003 | 1.00E-200 | 350470 | 0.454 | 1599.991 |
| Mean platelet (thrombocyte) volume | rs73000929 | 11 | 113953622 | A | G | 0.037 | 0.097 | 0.006 | 3.01E-54 | 350470 | 0.075 | 261.360 |
| Mean platelet (thrombocyte) volume | rs72947606 | 11 | 77383424 | C | G | 0.123 | -0.03 | 0.004 | 2.11E-16 | 350470 | 0.016 | 56.250 |
| Mean platelet (thrombocyte) volume | rs112744251 | 11 | 125587541 | T | C | 0.063 | -0.031 | 0.005 | 2.21E-10 | 350470 | 0.011 | 38.440 |
| Mean platelet (thrombocyte) volume | rs61905108 | 11 | 116624601 | A | C | 0.047 | 0.035 | 0.006 | 4.31E-10 | 350470 | 0.010 | 34.028 |
| Mean platelet (thrombocyte) volume | rs7729 | 11 | 94865454 | G | T | 0.641 | 0.014 | 0.002 | 9.22E-09 | 350470 | 0.014 | 49.000 |
| Mean platelet (thrombocyte) volume | rs7949566 | 11 | 126285301 | A | G | 0.422 | 0.037 | 0.002 | 6.84E-53 | 350470 | 0.098 | 342.248 |
| Mean platelet (thrombocyte) volume | rs75947414 | 12 | 123852510 | A | G | 0.116 | -0.035 | 0.004 | 8.11E-20 | 350470 | 0.022 | 76.562 |
| Mean platelet (thrombocyte) volume | rs758730 | 12 | 6314243 | C | T | 0.535 | -0.022 | 0.002 | 3.18E-19 | 350470 | 0.035 | 120.999 |
| Mean platelet (thrombocyte) volume | rs11168256 | 12 | 48217013 | G | A | 0.291 | -0.018 | 0.003 | 1.32E-11 | 350470 | 0.010 | 36.000 |
| Mean platelet (thrombocyte) volume | rs147903250 | 12 | 123938259 | T | C | 0.009 | 0.082 | 0.013 | 1.16E-09 | 350470 | 0.011 | 39.787 |
| Mean platelet (thrombocyte) volume | rs2230291 | 12 | 65141588 | T | C | 0.131 | 0.022 | 0.004 | 1.27E-09 | 350470 | 0.009 | 30.250 |
| Mean platelet (thrombocyte) volume | rs11061925 | 12 | 1803234 | T | C | 0.31 | -0.015 | 0.003 | 2.54E-09 | 350470 | 0.007 | 25.000 |
| Mean platelet (thrombocyte) volume | rs998908 | 12 | 6291892 | G | A | 0.494 | 0.104 | 0.002 | 1.00E-200 | 350470 | 0.766 | 2703.985 |
| Mean platelet (thrombocyte) volume | rs7952928 | 12 | 32828959 | A | G | 0.15 | -0.055 | 0.003 | 6.57E-61 | 350470 | 0.096 | 336.109 |
| Mean platelet (thrombocyte) volume | rs11544961 | 12 | 121868340 | T | C | 0.067 | 0.069 | 0.005 | 8.80E-46 | 350470 | 0.054 | 190.439 |
| Mean platelet (thrombocyte) volume | rs12230841 | 12 | 122475401 | A | G | 0.065 | -0.041 | 0.005 | 2.62E-17 | 350470 | 0.019 | 67.240 |
| Mean platelet (thrombocyte) volume | rs73219856 | 12 | 122618293 | T | C | 0.013 | -0.086 | 0.011 | 8.30E-16 | 350470 | 0.017 | 61.124 |
| Mean platelet (thrombocyte) volume | rs17199131 | 12 | 76963662 | T | C | 0.279 | -0.015 | 0.003 | 1.39E-08 | 350470 | 0.007 | 25.000 |
| Mean platelet (thrombocyte) volume | rs2950387 | 12 | 57021006 | C | T | 0.367 | -0.086 | 0.002 | 1.00E-200 | 350470 | 0.525 | 1848.989 |
| Mean platelet (thrombocyte) volume | rs7974597 | 12 | 49631531 | G | A | 0.076 | 0.099 | 0.005 | 3.44E-106 | 350470 | 0.112 | 392.038 |
| Mean platelet (thrombocyte) volume | rs2723261 | 12 | 40601808 | T | C | 0.66 | 0.019 | 0.003 | 3.64E-14 | 350470 | 0.011 | 40.111 |
| Mean platelet (thrombocyte) volume | rs2647908 | 12 | 64461875 | A | G | 0.664 | 0.018 | 0.003 | 3.23E-12 | 350470 | 0.010 | 36.000 |
| Mean platelet (thrombocyte) volume | rs2516211 | 12 | 121541160 | G | A | 0.791 | 0.02 | 0.003 | 1.67E-11 | 350470 | 0.013 | 44.444 |
| Mean platelet (thrombocyte) volume | rs10778463 | 12 | 106703716 | C | G | 0.77 | 0.016 | 0.003 | 2.41E-08 | 350470 | 0.008 | 28.444 |
| Mean platelet (thrombocyte) volume | rs10876550 | 12 | 54712308 | A | G | 0.559 | -0.099 | 0.002 | 1.00E-200 | 350470 | 0.694 | 2450.236 |
| Mean platelet (thrombocyte) volume | rs3858637 | 12 | 51694595 | G | T | 0.105 | -0.068 | 0.004 | 1.05E-67 | 350470 | 0.082 | 288.998 |
| Mean platelet (thrombocyte) volume | rs6489208 | 12 | 123180224 | G | C | 0.144 | 0.059 | 0.004 | 3.55E-60 | 350470 | 0.062 | 217.561 |
| Mean platelet (thrombocyte) volume | rs4964759 | 12 | 109203811 | A | G | 0.399 | -0.025 | 0.002 | 1.91E-25 | 350470 | 0.045 | 156.249 |
| Mean platelet (thrombocyte) volume | rs116891650 | 12 | 6154304 | T | C | 0.03 | 0.052 | 0.007 | 8.72E-14 | 350470 | 0.016 | 55.183 |
| Mean platelet (thrombocyte) volume | rs7133338 | 12 | 124780951 | A | G | 0.022 | 0.047 | 0.008 | 1.10E-08 | 350470 | 0.010 | 34.515 |
| Mean platelet (thrombocyte) volume | rs11553699 | 12 | 122216910 | G | A | 0.135 | 0.418 | 0.004 | 1.00E-200 | 350470 | 3.022 | 10920.188 |
| Mean platelet (thrombocyte) volume | rs2015599 | 12 | 29435480 | A | G | 0.46 | 0.079 | 0.002 | 1.00E-200 | 350470 | 0.443 | 1560.241 |
| Mean platelet (thrombocyte) volume | rs7296498 | 12 | 65038015 | C | G | 0.102 | -0.086 | 0.004 | 2.66E-105 | 350470 | 0.132 | 462.247 |
| Mean platelet (thrombocyte) volume | rs61922167 | 12 | 8189679 | G | C | 0.396 | 0.029 | 0.002 | 7.58E-33 | 350470 | 0.060 | 210.249 |
| Mean platelet (thrombocyte) volume | rs143858253 | 12 | 54497116 | C | T | 0.077 | 0.034 | 0.005 | 1.71E-13 | 350470 | 0.013 | 46.240 |
| Mean platelet (thrombocyte) volume | rs112811140 | 12 | 133092313 | T | C | 0.293 | 0.019 | 0.003 | 3.46E-13 | 350470 | 0.011 | 40.111 |
| Mean platelet (thrombocyte) volume | rs10466917 | 12 | 109977464 | G | T | 0.343 | 0.016 | 0.003 | 1.29E-10 | 350470 | 0.008 | 28.444 |
| Mean platelet (thrombocyte) volume | rs4762250 | 12 | 96278146 | G | A | 0.563 | -0.015 | 0.002 | 5.81E-10 | 350470 | 0.016 | 56.250 |
| Mean platelet (thrombocyte) volume | rs10744692 | 12 | 719136 | T | G | 0.16 | 0.019 | 0.003 | 6.30E-09 | 350470 | 0.011 | 40.111 |
| Mean platelet (thrombocyte) volume | rs4448753 | 12 | 80431544 | A | G | 0.009 | -0.072 | 0.013 | 2.61E-08 | 350470 | 0.009 | 30.674 |
| Mean platelet (thrombocyte) volume | rs9549753 | 13 | 114018038 | A | C | 0.532 | 0.048 | 0.002 | 6.14E-88 | 350470 | 0.164 | 575.997 |
| Mean platelet (thrombocyte) volume | rs9543534 | 13 | 74708696 | T | C | 0.733 | 0.019 | 0.003 | 7.01E-12 | 350470 | 0.011 | 40.111 |
| Mean platelet (thrombocyte) volume | rs66813596 | 13 | 76056606 | T | G | 0.265 | 0.018 | 0.003 | 9.69E-11 | 350470 | 0.010 | 36.000 |
| Mean platelet (thrombocyte) volume | rs9577481 | 13 | 113860900 | A | G | 0.177 | 0.027 | 0.003 | 3.77E-18 | 350470 | 0.023 | 81.000 |
| Mean platelet (thrombocyte) volume | rs7335710 | 13 | 33135424 | T | C | 0.371 | -0.032 | 0.002 | 6.08E-38 | 350470 | 0.073 | 255.999 |
| Mean platelet (thrombocyte) volume | rs4941565 | 13 | 47242912 | G | T | 0.652 | -0.032 | 0.003 | 3.06E-37 | 350470 | 0.032 | 113.777 |
| Mean platelet (thrombocyte) volume | rs76900683 | 13 | 28158035 | C | G | 0.049 | -0.054 | 0.006 | 1.65E-22 | 350470 | 0.023 | 81.000 |
| Mean platelet (thrombocyte) volume | rs57256419 | 14 | 51153621 | G | C | 0.151 | -0.037 | 0.003 | 1.93E-28 | 350470 | 0.043 | 152.110 |
| Mean platelet (thrombocyte) volume | rs35258120 | 14 | 103058769 | A | G | 0.819 | -0.029 | 0.003 | 2.24E-21 | 350470 | 0.027 | 93.444 |
| Mean platelet (thrombocyte) volume | rs149511257 | 14 | 69435176 | A | G | 0.008 | 0.088 | 0.014 | 1.16E-10 | 350470 | 0.011 | 39.510 |
| Mean platelet (thrombocyte) volume | rs2006676 | 14 | 53660236 | G | T | 0.825 | -0.026 | 0.003 | 1.73E-16 | 350470 | 0.021 | 75.111 |
| Mean platelet (thrombocyte) volume | rs1190333 | 14 | 103113414 | C | G | 0.969 | -0.04 | 0.007 | 6.34E-09 | 350470 | 0.009 | 32.653 |
| Mean platelet (thrombocyte) volume | rs10146346 | 14 | 93083572 | A | G | 0.408 | -0.014 | 0.002 | 7.48E-09 | 350470 | 0.014 | 49.000 |
| Mean platelet (thrombocyte) volume | rs2297066 | 14 | 103566835 | G | C | 0.242 | -0.078 | 0.003 | 7.14E-171 | 350470 | 0.193 | 675.996 |
| Mean platelet (thrombocyte) volume | rs74324636 | 14 | 55848253 | G | T | 0.084 | -0.082 | 0.004 | 8.58E-81 | 350470 | 0.120 | 420.248 |
| Mean platelet (thrombocyte) volume | rs28485304 | 14 | 69203017 | G | C | 0.352 | -0.031 | 0.003 | 7.10E-33 | 350470 | 0.030 | 106.777 |
| Mean platelet (thrombocyte) volume | rs12893404 | 14 | 65527122 | T | C | 0.313 | -0.025 | 0.003 | 8.07E-23 | 350470 | 0.020 | 69.444 |
| Mean platelet (thrombocyte) volume | rs74403919 | 14 | 92243978 | A | T | 0.173 | -0.039 | 0.003 | 2.38E-35 | 350470 | 0.048 | 168.999 |
| Mean platelet (thrombocyte) volume | rs10047928 | 14 | 31368063 | A | G | 0.254 | 0.031 | 0.003 | 3.43E-30 | 350470 | 0.030 | 106.777 |
| Mean platelet (thrombocyte) volume | rs4983594 | 14 | 105694870 | A | G | 0.853 | 0.037 | 0.004 | 2.56E-24 | 350470 | 0.024 | 85.562 |
| Mean platelet (thrombocyte) volume | rs12885436 | 14 | 23757540 | C | T | 0.628 | 0.018 | 0.002 | 2.01E-13 | 350470 | 0.023 | 81.000 |
| Mean platelet (thrombocyte) volume | rs7150606 | 14 | 68413076 | T | G | 0.14 | 0.034 | 0.003 | 8.17E-23 | 350470 | 0.037 | 128.444 |
| Mean platelet (thrombocyte) volume | rs11071720 | 15 | 63341996 | C | T | 0.702 | -0.057 | 0.003 | 5.15E-104 | 350470 | 0.103 | 360.998 |
| Mean platelet (thrombocyte) volume | rs12594925 | 15 | 91520287 | A | G | 0.128 | 0.046 | 0.004 | 7.11E-38 | 350470 | 0.038 | 132.249 |
| Mean platelet (thrombocyte) volume | rs12438271 | 15 | 89613227 | G | T | 0.287 | -0.024 | 0.003 | 2.09E-19 | 350470 | 0.018 | 64.000 |
| Mean platelet (thrombocyte) volume | rs140118737 | 15 | 90520917 | A | G | 0.025 | 0.06 | 0.008 | 1.74E-14 | 350470 | 0.016 | 56.250 |
| Mean platelet (thrombocyte) volume | rs61009920 | 15 | 65181280 | A | C | 0.137 | -0.053 | 0.003 | 1.53E-52 | 350470 | 0.089 | 312.109 |
| Mean platelet (thrombocyte) volume | rs3803462 | 15 | 74888495 | C | A | 0.149 | 0.027 | 0.003 | 4.92E-16 | 350470 | 0.023 | 81.000 |
| Mean platelet (thrombocyte) volume | rs12914876 | 15 | 49438415 | T | C | 0.214 | -0.021 | 0.003 | 6.80E-13 | 350470 | 0.014 | 49.000 |
| Mean platelet (thrombocyte) volume | rs3829483 | 15 | 34503867 | A | G | 0.135 | -0.02 | 0.003 | 1.18E-08 | 350470 | 0.013 | 44.444 |
| Mean platelet (thrombocyte) volume | rs5813801 | 15 | 75729518 | T | C | 0.249 | 0.023 | 0.003 | 2.40E-16 | 350470 | 0.017 | 58.777 |
| Mean platelet (thrombocyte) volume | rs2412653 | 15 | 42265236 | A | G | 0.885 | 0.026 | 0.004 | 1.20E-11 | 350470 | 0.012 | 42.250 |
| Mean platelet (thrombocyte) volume | rs11853810 | 15 | 57545292 | G | A | 0.158 | 0.032 | 0.003 | 8.40E-23 | 350470 | 0.032 | 113.777 |
| Mean platelet (thrombocyte) volume | rs112747606 | 15 | 66716106 | T | C | 0.225 | 0.02 | 0.003 | 2.85E-12 | 350470 | 0.013 | 44.444 |
| Mean platelet (thrombocyte) volume | rs12445050 | 16 | 81870969 | T | C | 0.138 | 0.058 | 0.003 | 7.51E-63 | 350470 | 0.107 | 373.776 |
| Mean platelet (thrombocyte) volume | rs151234 | 16 | 28505660 | C | G | 0.129 | -0.066 | 0.004 | 9.94E-76 | 350470 | 0.078 | 272.248 |
| Mean platelet (thrombocyte) volume | rs55877766 | 16 | 67473389 | A | G | 0.098 | 0.036 | 0.004 | 5.25E-19 | 350470 | 0.023 | 81.000 |
| Mean platelet (thrombocyte) volume | rs147649373 | 16 | 69789622 | T | A | 0.166 | 0.02 | 0.003 | 1.26E-09 | 350470 | 0.013 | 44.444 |
| Mean platelet (thrombocyte) volume | rs113038408 | 16 | 508573 | T | C | 0.063 | -0.031 | 0.005 | 2.43E-09 | 350470 | 0.011 | 38.440 |
| Mean platelet (thrombocyte) volume | rs11646743 | 16 | 5024041 | A | G | 0.485 | 0.033 | 0.002 | 2.69E-43 | 350470 | 0.078 | 272.248 |
| Mean platelet (thrombocyte) volume | rs9652625 | 16 | 53106223 | C | G | 0.77 | -0.026 | 0.003 | 8.21E-20 | 350470 | 0.021 | 75.111 |
| Mean platelet (thrombocyte) volume | rs2015319 | 16 | 4936280 | G | C | 0.575 | -0.018 | 0.002 | 7.34E-14 | 350470 | 0.023 | 81.000 |
| Mean platelet (thrombocyte) volume | rs80016599 | 16 | 24676401 | T | G | 0.009 | 0.09 | 0.013 | 5.98E-13 | 350470 | 0.014 | 47.929 |
| Mean platelet (thrombocyte) volume | rs72803037 | 16 | 85740078 | T | C | 0.356 | 0.014 | 0.003 | 4.19E-08 | 350470 | 0.006 | 21.778 |
| Mean platelet (thrombocyte) volume | rs12935775 | 16 | 89014871 | A | G | 0.371 | -0.022 | 0.002 | 6.75E-19 | 350470 | 0.035 | 120.999 |
| Mean platelet (thrombocyte) volume | rs55659628 | 16 | 85444655 | C | A | 0.666 | 0.017 | 0.003 | 2.82E-11 | 350470 | 0.009 | 32.111 |
| Mean platelet (thrombocyte) volume | rs34816462 | 16 | 9038761 | C | T | 0.188 | 0.028 | 0.003 | 1.77E-19 | 350470 | 0.025 | 87.111 |
| Mean platelet (thrombocyte) volume | rs59924446 | 16 | 53161774 | G | T | 0.329 | -0.019 | 0.003 | 2.20E-13 | 350470 | 0.011 | 40.111 |
| Mean platelet (thrombocyte) volume | rs72793380 | 16 | 30758743 | A | C | 0.105 | -0.023 | 0.004 | 5.43E-09 | 350470 | 0.009 | 33.062 |
| Mean platelet (thrombocyte) volume | rs150497606 | 17 | 55466426 | A | G | 0.05 | -0.069 | 0.006 | 1.59E-35 | 350470 | 0.038 | 132.249 |
| Mean platelet (thrombocyte) volume | rs939563 | 17 | 1415910 | A | T | 0.521 | 0.022 | 0.002 | 9.26E-21 | 350470 | 0.035 | 120.999 |
| Mean platelet (thrombocyte) volume | rs10438711 | 17 | 2478056 | T | A | 0.247 | -0.016 | 0.003 | 3.31E-09 | 350470 | 0.008 | 28.444 |
| Mean platelet (thrombocyte) volume | rs850736 | 17 | 42446420 | C | G | 0.374 | 0.052 | 0.002 | 1.39E-99 | 350470 | 0.193 | 675.996 |
| Mean platelet (thrombocyte) volume | rs62061820 | 17 | 44185431 | C | T | 0.227 | -0.033 | 0.003 | 8.94E-32 | 350470 | 0.035 | 120.999 |
| Mean platelet (thrombocyte) volume | rs59719244 | 17 | 62371243 | G | A | 0.517 | -0.027 | 0.002 | 3.98E-29 | 350470 | 0.052 | 182.249 |
| Mean platelet (thrombocyte) volume | rs8075385 | 17 | 27578514 | T | A | 0.165 | 0.028 | 0.003 | 7.77E-17 | 350470 | 0.025 | 87.111 |
| Mean platelet (thrombocyte) volume | rs4281782 | 17 | 16913281 | A | T | 0.468 | 0.02 | 0.002 | 5.97E-16 | 350470 | 0.029 | 99.999 |
| Mean platelet (thrombocyte) volume | rs2241233 | 17 | 7318396 | T | C | 0.861 | 0.021 | 0.004 | 1.56E-09 | 350470 | 0.008 | 27.562 |
| Mean platelet (thrombocyte) volume | rs73289314 | 17 | 1554636 | T | C | 0.016 | 0.056 | 0.009 | 3.19E-09 | 350470 | 0.011 | 38.716 |
| Mean platelet (thrombocyte) volume | rs1060431 | 17 | 4840868 | A | G | 0.073 | -0.058 | 0.005 | 6.40E-36 | 350470 | 0.038 | 134.559 |
| Mean platelet (thrombocyte) volume | rs11652957 | 17 | 45419139 | G | A | 0.217 | -0.034 | 0.003 | 1.50E-31 | 350470 | 0.037 | 128.444 |
| Mean platelet (thrombocyte) volume | rs79007502 | 17 | 33880305 | C | T | 0.038 | -0.101 | 0.006 | 2.00E-59 | 350470 | 0.081 | 283.359 |
| Mean platelet (thrombocyte) volume | rs55868394 | 17 | 73851113 | A | C | 0.127 | 0.023 | 0.004 | 3.02E-10 | 350470 | 0.009 | 33.062 |
| Mean platelet (thrombocyte) volume | rs3760221 | 17 | 65713369 | T | C | 0.555 | -0.014 | 0.002 | 6.71E-09 | 350470 | 0.014 | 49.000 |
| Mean platelet (thrombocyte) volume | rs28437451 | 17 | 28568301 | A | G | 0.021 | 0.05 | 0.009 | 1.04E-08 | 350470 | 0.009 | 30.864 |
| Mean platelet (thrombocyte) volume | rs8081187 | 17 | 29016360 | A | G | 0.083 | -0.048 | 0.004 | 1.57E-28 | 350470 | 0.041 | 143.999 |
| Mean platelet (thrombocyte) volume | rs149370750 | 17 | 80538796 | T | C | 0.059 | 0.04 | 0.005 | 5.66E-14 | 350470 | 0.018 | 64.000 |
| Mean platelet (thrombocyte) volume | rs238241 | 17 | 4851072 | G | A | 0.976 | -0.047 | 0.008 | 4.86E-09 | 350470 | 0.010 | 34.515 |
| Mean platelet (thrombocyte) volume | rs8099412 | 18 | 67536617 | C | T | 0.475 | -0.084 | 0.002 | 1.00E-200 | 350470 | 0.501 | 1763.990 |
| Mean platelet (thrombocyte) volume | rs1052025 | 18 | 77287776 | G | A | 0.06 | 0.048 | 0.005 | 3.01E-21 | 350470 | 0.026 | 92.159 |
| Mean platelet (thrombocyte) volume | rs11082304 | 18 | 20720973 | T | G | 0.514 | 0.046 | 0.002 | 1.93E-84 | 350470 | 0.151 | 528.997 |
| Mean platelet (thrombocyte) volume | rs671339 | 18 | 9618005 | G | A | 0.737 | -0.05 | 0.003 | 7.11E-77 | 350470 | 0.079 | 277.776 |
| Mean platelet (thrombocyte) volume | rs3937015 | 18 | 67599516 | C | T | 0.981 | -0.134 | 0.009 | 6.08E-53 | 350470 | 0.063 | 221.678 |
| Mean platelet (thrombocyte) volume | rs17758695 | 18 | 60920854 | T | C | 0.03 | -0.089 | 0.007 | 2.99E-36 | 350470 | 0.046 | 161.652 |
| Mean platelet (thrombocyte) volume | rs11664743 | 18 | 43837255 | A | C | 0.359 | 0.026 | 0.002 | 7.49E-26 | 350470 | 0.048 | 168.999 |
| Mean platelet (thrombocyte) volume | rs28782011 | 18 | 41997547 | T | G | 0.13 | 0.033 | 0.004 | 4.14E-20 | 350470 | 0.019 | 68.062 |
| Mean platelet (thrombocyte) volume | rs145875564 | 18 | 77154009 | A | G | 0.028 | -0.064 | 0.008 | 1.52E-16 | 350470 | 0.018 | 64.000 |
| Mean platelet (thrombocyte) volume | rs11662485 | 18 | 77276417 | A | G | 0.057 | -0.032 | 0.005 | 9.96E-10 | 350470 | 0.012 | 40.960 |
| Mean platelet (thrombocyte) volume | rs2043040 | 18 | 57309952 | T | C | 0.56 | 0.014 | 0.002 | 2.23E-09 | 350470 | 0.014 | 49.000 |
| Mean platelet (thrombocyte) volume | rs7254827 | 19 | 17202746 | T | A | 0.225 | 0.038 | 0.003 | 2.05E-40 | 350470 | 0.046 | 160.444 |
| Mean platelet (thrombocyte) volume | rs7248710 | 19 | 35665648 | A | C | 0.718 | 0.03 | 0.003 | 4.02E-29 | 350470 | 0.029 | 99.999 |
| Mean platelet (thrombocyte) volume | rs12978499 | 19 | 49237552 | C | A | 0.413 | 0.023 | 0.002 | 2.41E-21 | 350470 | 0.038 | 132.249 |
| Mean platelet (thrombocyte) volume | rs11672416 | 19 | 46206425 | C | T | 0.112 | 0.025 | 0.004 | 9.46E-11 | 350470 | 0.011 | 39.062 |
| Mean platelet (thrombocyte) volume | rs892090 | 19 | 55539072 | G | T | 0.835 | 0.063 | 0.003 | 1.35E-84 | 350470 | 0.126 | 440.997 |
| Mean platelet (thrombocyte) volume | rs11879091 | 19 | 1082265 | T | C | 0.518 | 0.025 | 0.002 | 9.70E-25 | 350470 | 0.045 | 156.249 |
| Mean platelet (thrombocyte) volume | rs74390626 | 19 | 45126457 | T | C | 0.025 | 0.065 | 0.008 | 2.79E-17 | 350470 | 0.019 | 66.015 |
| Mean platelet (thrombocyte) volume | rs41290102 | 19 | 45371188 | T | C | 0.014 | 0.057 | 0.01 | 1.97E-08 | 350470 | 0.009 | 32.490 |
| Mean platelet (thrombocyte) volume | rs11667509 | 19 | 45716192 | C | G | 0.377 | 0.068 | 0.002 | 3.13E-165 | 350470 | 0.329 | 1155.993 |
| Mean platelet (thrombocyte) volume | rs7258141 | 19 | 33099414 | T | A | 0.648 | 0.022 | 0.003 | 5.47E-17 | 350470 | 0.015 | 53.777 |
| Mean platelet (thrombocyte) volume | rs77808283 | 19 | 16189882 | A | G | 0.039 | -0.054 | 0.006 | 1.66E-18 | 350470 | 0.023 | 81.000 |
| Mean platelet (thrombocyte) volume | rs8182597 | 19 | 39250832 | T | A | 0.251 | -0.018 | 0.003 | 4.39E-11 | 350470 | 0.010 | 36.000 |
| Mean platelet (thrombocyte) volume | rs62116856 | 19 | 16210265 | A | G | 0.039 | 0.041 | 0.007 | 3.96E-10 | 350470 | 0.010 | 34.306 |
| Mean platelet (thrombocyte) volume | rs7252328 | 19 | 2003785 | G | A | 0.153 | -0.019 | 0.003 | 4.36E-08 | 350470 | 0.011 | 40.111 |
| Mean platelet (thrombocyte) volume | rs57843631 | 19 | 16206012 | T | C | 0.019 | 0.265 | 0.009 | 8.45E-190 | 350470 | 0.247 | 866.970 |
| Mean platelet (thrombocyte) volume | rs8106212 | 19 | 6802571 | T | C | 0.012 | 0.146 | 0.011 | 2.50E-39 | 350470 | 0.050 | 176.164 |
| Mean platelet (thrombocyte) volume | rs11673069 | 19 | 58934918 | G | C | 0.218 | -0.026 | 0.003 | 8.70E-19 | 350470 | 0.021 | 75.111 |
| Mean platelet (thrombocyte) volume | rs1110621 | 19 | 6060731 | C | T | 0.367 | 0.017 | 0.002 | 2.46E-12 | 350470 | 0.021 | 72.250 |
| Mean platelet (thrombocyte) volume | rs4814779 | 20 | 1923271 | A | C | 0.266 | -0.077 | 0.003 | 1.61E-177 | 350470 | 0.188 | 658.774 |
| Mean platelet (thrombocyte) volume | rs1369236 | 20 | 57779762 | G | A | 0.089 | -0.049 | 0.004 | 6.35E-31 | 350470 | 0.043 | 150.062 |
| Mean platelet (thrombocyte) volume | rs373018 | 20 | 44456059 | C | T | 0.549 | 0.022 | 0.002 | 2.93E-20 | 350470 | 0.035 | 120.999 |
| Mean platelet (thrombocyte) volume | rs7265519 | 20 | 43515270 | T | C | 0.267 | 0.031 | 0.003 | 6.30E-30 | 350470 | 0.030 | 106.777 |
| Mean platelet (thrombocyte) volume | rs16982339 | 20 | 57538175 | G | A | 0.209 | -0.022 | 0.003 | 1.35E-12 | 350470 | 0.015 | 53.777 |
| Mean platelet (thrombocyte) volume | rs6514693 | 20 | 16561360 | A | G | 0.13 | -0.024 | 0.004 | 7.75E-12 | 350470 | 0.010 | 36.000 |
| Mean platelet (thrombocyte) volume | rs156356 | 20 | 1819280 | C | T | 0.457 | 0.059 | 0.002 | 7.76E-128 | 350470 | 0.248 | 870.245 |
| Mean platelet (thrombocyte) volume | rs17726337 | 20 | 45944659 | C | G | 0.037 | -0.083 | 0.006 | 6.90E-39 | 350470 | 0.055 | 191.360 |
| Mean platelet (thrombocyte) volume | rs8115191 | 20 | 56019801 | T | C | 0.425 | -0.025 | 0.002 | 2.48E-24 | 350470 | 0.045 | 156.249 |
| Mean platelet (thrombocyte) volume | rs6081569 | 20 | 19303786 | C | G | 0.368 | 0.024 | 0.002 | 3.35E-22 | 350470 | 0.041 | 143.999 |
| Mean platelet (thrombocyte) volume | rs67784641 | 20 | 1477457 | C | A | 0.125 | -0.026 | 0.004 | 7.49E-13 | 350470 | 0.012 | 42.250 |
| Mean platelet (thrombocyte) volume | rs6070930 | 20 | 58301006 | T | C | 0.123 | 0.021 | 0.004 | 7.63E-09 | 350470 | 0.008 | 27.562 |
| Mean platelet (thrombocyte) volume | rs118062269 | 20 | 1590770 | G | A | 0.777 | 0.04 | 0.003 | 9.23E-45 | 350470 | 0.051 | 177.777 |
| Mean platelet (thrombocyte) volume | rs11702425 | 21 | 46908355 | C | T | 0.303 | -0.015 | 0.003 | 1.34E-08 | 350470 | 0.007 | 25.000 |
| Mean platelet (thrombocyte) volume | rs2242885 | 21 | 36386766 | T | C | 0.297 | -0.019 | 0.003 | 2.51E-13 | 350470 | 0.011 | 40.111 |
| Mean platelet (thrombocyte) volume | rs1736013 | 21 | 16804330 | C | G | 0.432 | -0.018 | 0.002 | 2.09E-14 | 350470 | 0.023 | 81.000 |
| Mean platelet (thrombocyte) volume | rs5758910 | 22 | 43145853 | C | G | 0.676 | -0.033 | 0.003 | 1.58E-37 | 350470 | 0.035 | 120.999 |
| Mean platelet (thrombocyte) volume | rs972577 | 22 | 43387645 | T | C | 0.552 | 0.061 | 0.002 | 1.17E-144 | 350470 | 0.265 | 930.245 |
| Mean platelet (thrombocyte) volume | rs2097598 | 22 | 19689697 | G | C | 0.377 | 0.04 | 0.003 | 1.25E-54 | 350470 | 0.051 | 177.777 |
| Mean platelet (thrombocyte) volume | rs4824112 | 22 | 50284345 | G | A | 0.192 | -0.029 | 0.003 | 4.03E-22 | 350470 | 0.027 | 93.444 |
| Mean platelet (thrombocyte) volume | rs9306345 | 22 | 40832375 | G | A | 0.148 | 0.019 | 0.003 | 3.09E-08 | 350470 | 0.011 | 40.111 |
| Mean platelet (thrombocyte) volume | rs5993717 | 22 | 19582021 | T | G | 0.231 | 0.017 | 0.003 | 1.74E-09 | 350470 | 0.009 | 32.111 |
| Mean platelet (thrombocyte) volume | rs9616906 | 22 | 51104680 | A | G | 0.439 | -0.031 | 0.002 | 2.40E-38 | 350470 | 0.069 | 240.249 |
| Mean platelet (thrombocyte) volume | rs140064 | 22 | 29689837 | G | A | 0.779 | -0.035 | 0.003 | 2.19E-33 | 350470 | 0.039 | 136.110 |
| Mean platelet (thrombocyte) volume | rs73152621 | 22 | 23527908 | T | G | 0.264 | 0.015 | 0.003 | 3.31E-08 | 350470 | 0.007 | 25.000 |
| Mean platelet (thrombocyte) volume | rs742396 | 22 | 38452254 | G | C | 0.625 | 0.015 | 0.002 | 1.87E-09 | 350470 | 0.016 | 56.250 |
| Platelet count | rs1768584 | 1 | 205240145 | G | A | 0.386 | 0.051 | 0.002 | 1.36E-102 | 350474 | 0.185 | 650.246 |
| Platelet count | rs55794721 | 1 | 25561667 | A | G | 0.407 | -0.035 | 0.002 | 3.23E-50 | 350474 | 0.087 | 306.248 |
| Platelet count | rs34789477 | 1 | 27000235 | G | A | 0.096 | 0.036 | 0.004 | 9.94E-20 | 350474 | 0.023 | 81.000 |
| Platelet count | rs76064941 | 1 | 171851052 | T | C | 0.032 | -0.058 | 0.007 | 6.24E-19 | 350474 | 0.020 | 68.653 |
| Platelet count | rs17625587 | 1 | 198990494 | A | G | 0.262 | -0.022 | 0.003 | 2.60E-17 | 350474 | 0.015 | 53.777 |
| Platelet count | rs67149561 | 1 | 204929196 | C | G | 0.156 | 0.022 | 0.003 | 2.84E-12 | 350474 | 0.015 | 53.777 |
| Platelet count | rs11204543 | 1 | 248058761 | C | G | 0.341 | -0.017 | 0.002 | 3.38E-12 | 350474 | 0.021 | 72.250 |
| Platelet count | rs78261031 | 1 | 156112470 | A | G | 0.059 | -0.033 | 0.005 | 4.37E-11 | 350474 | 0.012 | 43.560 |
| Platelet count | rs3122414 | 1 | 40520931 | A | G | 0.666 | -0.014 | 0.002 | 5.78E-09 | 350474 | 0.014 | 49.000 |
| Platelet count | rs6425522 | 1 | 171946082 | C | T | 0.802 | -0.067 | 0.003 | 1.20E-118 | 350474 | 0.142 | 498.775 |
| Platelet count | rs12041331 | 1 | 156869714 | A | G | 0.084 | 0.038 | 0.004 | 1.64E-19 | 350474 | 0.026 | 90.249 |
| Platelet count | rs12119893 | 1 | 10483167 | A | G | 0.136 | -0.031 | 0.004 | 2.31E-18 | 350474 | 0.017 | 60.062 |
| Platelet count | rs12136856 | 1 | 156473114 | G | C | 0.652 | -0.021 | 0.002 | 2.72E-17 | 350474 | 0.031 | 110.249 |
| Platelet count | rs12042229 | 1 | 150447398 | G | A | 0.394 | 0.02 | 0.002 | 1.11E-16 | 350474 | 0.029 | 99.999 |
| Platelet count | rs4357501 | 1 | 213948225 | T | C | 0.616 | -0.016 | 0.002 | 4.98E-11 | 350474 | 0.018 | 64.000 |
| Platelet count | rs3767809 | 1 | 118154831 | C | T | 0.221 | 0.039 | 0.003 | 6.90E-44 | 350474 | 0.048 | 168.999 |
| Platelet count | rs12045264 | 1 | 248021747 | A | G | 0.632 | -0.031 | 0.002 | 8.82E-38 | 350474 | 0.069 | 240.249 |
| Platelet count | rs79898419 | 1 | 91587561 | G | A | 0.246 | 0.023 | 0.003 | 3.08E-18 | 350474 | 0.017 | 58.777 |
| Platelet count | rs4477285 | 1 | 94876928 | A | G | 0.421 | 0.019 | 0.002 | 2.61E-16 | 350474 | 0.026 | 90.249 |
| Platelet count | rs12126199 | 1 | 43859227 | T | C | 0.034 | 0.051 | 0.006 | 2.37E-15 | 350474 | 0.021 | 72.250 |
| Platelet count | rs1933295 | 1 | 62107021 | G | A | 0.776 | -0.019 | 0.003 | 4.55E-12 | 350474 | 0.011 | 40.111 |
| Platelet count | rs56333341 | 1 | 68054209 | T | A | 0.061 | -0.03 | 0.005 | 3.67E-10 | 350474 | 0.010 | 36.000 |
| Platelet count | rs1926310 | 1 | 65188498 | C | G | 0.236 | -0.016 | 0.003 | 4.49E-09 | 350474 | 0.008 | 28.444 |
| Platelet count | rs112096491 | 1 | 234735980 | T | C | 0.553 | 0.013 | 0.002 | 2.86E-08 | 350474 | 0.012 | 42.250 |
| Platelet count | rs61781296 | 1 | 40419732 | T | C | 0.262 | 0.027 | 0.003 | 3.13E-25 | 350474 | 0.023 | 81.000 |
| Platelet count | rs4846914 | 1 | 230295691 | A | G | 0.607 | 0.024 | 0.002 | 8.63E-24 | 350474 | 0.041 | 143.999 |
| Platelet count | rs2494663 | 1 | 154092056 | A | C | 0.551 | -0.019 | 0.002 | 4.98E-16 | 350474 | 0.026 | 90.249 |
| Platelet count | rs4916186 | 1 | 172396020 | G | A | 0.499 | 0.017 | 0.002 | 7.60E-13 | 350474 | 0.021 | 72.250 |
| Platelet count | rs630505 | 1 | 111737916 | C | T | 0.265 | 0.018 | 0.003 | 8.44E-12 | 350474 | 0.010 | 36.000 |
| Platelet count | rs28706215 | 1 | 236702973 | T | A | 0.695 | -0.017 | 0.003 | 2.39E-11 | 350474 | 0.009 | 32.111 |
| Platelet count | rs2355710 | 1 | 45166513 | A | T | 0.202 | -0.016 | 0.003 | 2.06E-08 | 350474 | 0.008 | 28.444 |
| Platelet count | rs56043070 | 1 | 247719769 | A | G | 0.072 | -0.127 | 0.004 | 3.24E-178 | 350474 | 0.287 | 1008.057 |
| Platelet count | rs2236055 | 1 | 12042261 | G | A | 0.468 | -0.038 | 0.002 | 5.25E-61 | 350474 | 0.103 | 360.998 |
| Platelet count | rs12065863 | 1 | 45852564 | C | T | 0.251 | -0.037 | 0.003 | 1.95E-43 | 350474 | 0.043 | 152.110 |
| Platelet count | rs2236074 | 1 | 28211384 | A | G | 0.361 | -0.021 | 0.002 | 4.89E-18 | 350474 | 0.031 | 110.249 |
| Platelet count | rs28549287 | 1 | 110230138 | A | G | 0.774 | 0.023 | 0.003 | 7.65E-15 | 350474 | 0.017 | 58.777 |
| Platelet count | rs945631 | 1 | 93426167 | A | G | 0.041 | -0.044 | 0.006 | 5.47E-14 | 350474 | 0.015 | 53.777 |
| Platelet count | rs661126 | 1 | 93678968 | G | T | 0.625 | 0.016 | 0.002 | 1.55E-11 | 350474 | 0.018 | 64.000 |
| Platelet count | rs7526446 | 1 | 63167984 | A | C | 0.637 | -0.015 | 0.002 | 6.94E-10 | 350474 | 0.016 | 56.250 |
| Platelet count | rs35181127 | 1 | 161590743 | G | A | 0.509 | -0.014 | 0.002 | 1.96E-09 | 350474 | 0.014 | 49.000 |
| Platelet count | rs655029 | 2 | 31477838 | A | G | 0.708 | 0.074 | 0.003 | 1.83E-183 | 350474 | 0.173 | 608.441 |
| Platelet count | rs1047891 | 2 | 211540507 | A | C | 0.316 | -0.035 | 0.002 | 2.88E-44 | 350474 | 0.087 | 306.248 |
| Platelet count | rs12052715 | 2 | 160677375 | G | C | 0.727 | -0.03 | 0.003 | 9.93E-31 | 350474 | 0.029 | 99.999 |
| Platelet count | rs11686139 | 2 | 227291415 | C | A | 0.194 | 0.031 | 0.003 | 8.32E-27 | 350474 | 0.030 | 106.777 |
| Platelet count | rs939136 | 2 | 66677531 | C | G | 0.663 | 0.024 | 0.002 | 2.84E-22 | 350474 | 0.041 | 143.999 |
| Platelet count | rs6547617 | 2 | 85655402 | A | T | 0.567 | -0.017 | 0.002 | 4.43E-13 | 350474 | 0.021 | 72.250 |
| Platelet count | rs893808 | 2 | 198860837 | G | C | 0.671 | 0.017 | 0.002 | 1.91E-11 | 350474 | 0.021 | 72.250 |
| Platelet count | rs16844866 | 2 | 161063407 | C | T | 0.177 | 0.02 | 0.003 | 3.11E-11 | 350474 | 0.013 | 44.444 |
| Platelet count | rs78909033 | 2 | 241510903 | A | G | 0.135 | 0.064 | 0.003 | 7.28E-80 | 350474 | 0.130 | 455.109 |
| Platelet count | rs149290349 | 2 | 43451957 | A | G | 0.075 | -0.071 | 0.004 | 6.19E-57 | 350474 | 0.090 | 315.061 |
| Platelet count | rs4849845 | 2 | 121013169 | A | G | 0.688 | -0.032 | 0.002 | 1.96E-37 | 350474 | 0.073 | 255.999 |
| Platelet count | rs6706095 | 2 | 46075677 | G | T | 0.76 | 0.024 | 0.003 | 2.13E-19 | 350474 | 0.018 | 64.000 |
| Platelet count | rs7585866 | 2 | 192696255 | G | A | 0.353 | 0.02 | 0.002 | 8.37E-17 | 350474 | 0.029 | 99.999 |
| Platelet count | rs35330522 | 2 | 12877060 | A | G | 0.477 | 0.018 | 0.002 | 2.08E-15 | 350474 | 0.023 | 81.000 |
| Platelet count | rs72932729 | 2 | 203650267 | T | C | 0.475 | 0.018 | 0.002 | 2.49E-15 | 350474 | 0.023 | 81.000 |
| Platelet count | rs10048745 | 2 | 68962137 | A | G | 0.256 | 0.019 | 0.003 | 1.63E-12 | 350474 | 0.011 | 40.111 |
| Platelet count | rs2714483 | 2 | 202362955 | G | A | 0.394 | -0.015 | 0.002 | 3.27E-10 | 350474 | 0.016 | 56.250 |
| Platelet count | rs4853721 | 2 | 191477846 | T | C | 0.167 | -0.018 | 0.003 | 4.65E-09 | 350474 | 0.010 | 36.000 |
| Platelet count | rs2289321 | 2 | 111870220 | C | T | 0.063 | 0.026 | 0.005 | 3.05E-08 | 350474 | 0.008 | 27.040 |
| Platelet count | rs7349311 | 2 | 70165966 | A | G | 0.187 | -0.033 | 0.003 | 4.70E-28 | 350474 | 0.035 | 120.999 |
| Platelet count | rs10865130 | 2 | 38046787 | G | C | 0.322 | -0.02 | 0.002 | 9.04E-16 | 350474 | 0.029 | 99.999 |
| Platelet count | rs55664157 | 2 | 234274654 | A | T | 0.122 | -0.028 | 0.004 | 2.44E-15 | 350474 | 0.014 | 49.000 |
| Platelet count | rs16858573 | 2 | 143875725 | C | T | 0.125 | -0.025 | 0.004 | 6.38E-13 | 350474 | 0.011 | 39.062 |
| Platelet count | rs6744560 | 2 | 37130658 | C | A | 0.37 | 0.016 | 0.002 | 6.60E-12 | 350474 | 0.018 | 64.000 |
| Platelet count | rs6759839 | 2 | 16620803 | G | A | 0.678 | 0.015 | 0.002 | 1.16E-09 | 350474 | 0.016 | 56.250 |
| Platelet count | rs1820583 | 2 | 145425249 | C | T | 0.919 | 0.024 | 0.004 | 1.15E-08 | 350474 | 0.010 | 36.000 |
| Platelet count | rs4296389 | 2 | 21142994 | T | C | 0.246 | 0.015 | 0.003 | 2.90E-08 | 350474 | 0.007 | 25.000 |
| Platelet count | rs1260326 | 2 | 27730940 | C | T | 0.607 | -0.042 | 0.002 | 2.87E-72 | 350474 | 0.126 | 440.997 |
| Platelet count | rs17572109 | 2 | 219093934 | A | G | 0.234 | 0.037 | 0.003 | 1.11E-40 | 350474 | 0.043 | 152.110 |
| Platelet count | rs72627489 | 2 | 110445505 | T | C | 0.096 | -0.032 | 0.004 | 1.10E-15 | 350474 | 0.018 | 64.000 |
| Platelet count | rs12621712 | 2 | 74716140 | G | A | 0.142 | 0.026 | 0.003 | 1.05E-14 | 350474 | 0.021 | 75.111 |
| Platelet count | rs113889746 | 2 | 208408748 | G | A | 0.33 | -0.016 | 0.002 | 2.61E-10 | 350474 | 0.018 | 64.000 |
| Platelet count | rs6545465 | 2 | 55212337 | C | T | 0.808 | -0.019 | 0.003 | 3.08E-10 | 350474 | 0.011 | 40.111 |
| Platelet count | rs12614923 | 2 | 170616257 | G | A | 0.376 | 0.014 | 0.002 | 2.82E-09 | 350474 | 0.014 | 49.000 |
| Platelet count | rs7618405 | 3 | 18250509 | A | C | 0.209 | -0.042 | 0.003 | 3.50E-48 | 350474 | 0.056 | 195.999 |
| Platelet count | rs2046823 | 3 | 56779011 | A | G | 0.264 | 0.036 | 0.003 | 8.33E-43 | 350474 | 0.041 | 143.999 |
| Platelet count | rs56268840 | 3 | 12271381 | T | C | 0.274 | -0.03 | 0.003 | 4.45E-31 | 350474 | 0.029 | 99.999 |
| Platelet count | rs62251184 | 3 | 56674328 | T | C | 0.063 | 0.047 | 0.005 | 2.35E-22 | 350474 | 0.025 | 88.359 |
| Platelet count | rs7630419 | 3 | 101133037 | A | G | 0.404 | -0.016 | 0.002 | 2.05E-11 | 350474 | 0.018 | 64.000 |
| Platelet count | rs62240976 | 3 | 18487337 | G | C | 0.091 | -0.027 | 0.004 | 5.05E-11 | 350474 | 0.013 | 45.562 |
| Platelet count | rs12490795 | 3 | 15694630 | C | G | 0.087 | 0.025 | 0.004 | 6.28E-10 | 350474 | 0.011 | 39.062 |
| Platelet count | rs9879207 | 3 | 126584403 | C | T | 0.168 | 0.018 | 0.003 | 5.38E-09 | 350474 | 0.010 | 36.000 |
| Platelet count | rs1354034 | 3 | 56849749 | C | T | 0.601 | 0.137 | 0.002 | 1.00E-200 | 350474 | 1.321 | 4692.223 |
| Platelet count | rs6141 | 3 | 184090266 | T | C | 0.53 | 0.056 | 0.002 | 2.78E-126 | 350474 | 0.223 | 783.996 |
| Platelet count | rs3796215 | 3 | 58316657 | G | A | 0.322 | -0.032 | 0.002 | 2.22E-39 | 350474 | 0.073 | 255.999 |
| Platelet count | rs9809116 | 3 | 72397279 | G | A | 0.407 | 0.022 | 0.002 | 2.48E-20 | 350474 | 0.035 | 120.999 |
| Platelet count | rs13084357 | 3 | 184094501 | C | T | 0.084 | 0.036 | 0.004 | 7.61E-18 | 350474 | 0.023 | 81.000 |
| Platelet count | rs167924 | 3 | 107379837 | G | A | 0.621 | 0.018 | 0.002 | 1.05E-14 | 350474 | 0.023 | 81.000 |
| Platelet count | rs6444571 | 3 | 167439397 | C | T | 0.26 | 0.018 | 0.003 | 1.04E-11 | 350474 | 0.010 | 36.000 |
| Platelet count | rs13084317 | 3 | 39187858 | A | G | 0.33 | 0.015 | 0.002 | 4.98E-10 | 350474 | 0.016 | 56.250 |
| Platelet count | rs115243514 | 3 | 183926133 | C | G | 0.018 | 0.109 | 0.009 | 3.92E-34 | 350474 | 0.042 | 146.678 |
| Platelet count | rs7641761 | 3 | 178740422 | A | T | 0.697 | -0.023 | 0.003 | 3.05E-19 | 350474 | 0.017 | 58.777 |
| Platelet count | rs1602456 | 3 | 56541253 | A | G | 0.021 | -0.069 | 0.008 | 7.37E-18 | 350474 | 0.021 | 74.390 |
| Platelet count | rs7640747 | 3 | 37596805 | G | C | 0.372 | -0.015 | 0.002 | 3.47E-10 | 350474 | 0.016 | 56.250 |
| Platelet count | rs4679703 | 3 | 152997867 | A | C | 0.483 | 0.013 | 0.002 | 5.72E-09 | 350474 | 0.012 | 42.250 |
| Platelet count | rs3804749 | 3 | 122833003 | T | C | 0.593 | -0.033 | 0.002 | 7.20E-45 | 350474 | 0.078 | 272.248 |
| Platelet count | rs2859868 | 3 | 168860010 | A | G | 0.55 | -0.02 | 0.002 | 1.74E-18 | 350474 | 0.029 | 99.999 |
| Platelet count | rs9829114 | 3 | 196518623 | A | G | 0.418 | -0.018 | 0.002 | 6.78E-14 | 350474 | 0.023 | 81.000 |
| Platelet count | rs2343681 | 3 | 136535024 | A | G | 0.79 | 0.019 | 0.003 | 4.58E-11 | 350474 | 0.011 | 40.111 |
| Platelet count | rs13078720 | 3 | 18730252 | T | C | 0.165 | 0.02 | 0.003 | 2.01E-10 | 350474 | 0.013 | 44.444 |
| Platelet count | rs13322435 | 3 | 156795468 | G | A | 0.401 | 0.014 | 0.002 | 1.50E-09 | 350474 | 0.014 | 49.000 |
| Platelet count | rs76445378 | 3 | 124366890 | T | C | 0.016 | 0.1 | 0.009 | 3.47E-27 | 350474 | 0.035 | 123.456 |
| Platelet count | rs11917130 | 3 | 69855694 | A | T | 0.648 | -0.018 | 0.002 | 4.87E-14 | 350474 | 0.023 | 81.000 |
| Platelet count | rs721412 | 4 | 110947478 | G | A | 0.319 | -0.027 | 0.002 | 5.61E-27 | 350474 | 0.052 | 182.249 |
| Platelet count | rs113128512 | 4 | 157682188 | C | T | 0.092 | -0.029 | 0.004 | 4.29E-13 | 350474 | 0.015 | 52.562 |
| Platelet count | rs868489 | 4 | 7055253 | T | C | 0.238 | -0.017 | 0.003 | 1.80E-10 | 350474 | 0.009 | 32.111 |
| Platelet count | rs144317085 | 4 | 105806108 | T | A | 0.034 | 0.039 | 0.006 | 2.20E-09 | 350474 | 0.012 | 42.250 |
| Platelet count | rs11731274 | 4 | 6891455 | G | T | 0.176 | 0.052 | 0.003 | 1.50E-65 | 350474 | 0.086 | 300.443 |
| Platelet count | rs7665147 | 4 | 57767327 | A | T | 0.186 | -0.03 | 0.003 | 1.31E-23 | 350474 | 0.029 | 99.999 |
| Platelet count | rs3912392 | 4 | 39698738 | A | G | 0.507 | -0.016 | 0.002 | 1.20E-11 | 350474 | 0.018 | 64.000 |
| Platelet count | rs2315560 | 4 | 17677305 | G | C | 0.588 | -0.017 | 0.002 | 3.42E-13 | 350474 | 0.021 | 72.250 |
| Platelet count | rs2017058 | 4 | 120257739 | C | T | 0.512 | -0.017 | 0.002 | 8.14E-13 | 350474 | 0.021 | 72.250 |
| Platelet count | rs6851940 | 4 | 2767234 | G | C | 0.367 | 0.013 | 0.002 | 4.86E-08 | 350474 | 0.012 | 42.250 |
| Platelet count | rs71633359 | 4 | 88183820 | C | T | 0.32 | 0.026 | 0.003 | 3.97E-25 | 350474 | 0.021 | 75.111 |
| Platelet count | rs113693454 | 4 | 106207763 | A | T | 0.032 | -0.063 | 0.007 | 1.83E-21 | 350474 | 0.023 | 81.000 |
| Platelet count | rs2724564 | 4 | 152349962 | T | G | 0.561 | 0.022 | 0.002 | 7.69E-21 | 350474 | 0.035 | 120.999 |
| Platelet count | rs13108218 | 4 | 3443931 | G | A | 0.618 | -0.022 | 0.002 | 2.94E-19 | 350474 | 0.035 | 120.999 |
| Platelet count | rs363096 | 4 | 3180021 | C | T | 0.574 | -0.018 | 0.002 | 2.11E-14 | 350474 | 0.023 | 81.000 |
| Platelet count | rs17425951 | 4 | 124788456 | A | G | 0.109 | 0.026 | 0.004 | 1.99E-12 | 350474 | 0.012 | 42.250 |
| Platelet count | rs6857262 | 4 | 145026050 | G | T | 0.464 | 0.015 | 0.002 | 2.75E-10 | 350474 | 0.016 | 56.250 |
| Platelet count | rs34592828 | 5 | 75996909 | A | G | 0.046 | -0.149 | 0.006 | 4.03E-160 | 350474 | 0.176 | 616.691 |
| Platelet count | rs6556405 | 5 | 158635102 | C | T | 0.249 | -0.035 | 0.003 | 1.04E-38 | 350474 | 0.039 | 136.110 |
| Platelet count | rs13357739 | 5 | 176745683 | A | T | 0.024 | 0.062 | 0.008 | 5.91E-16 | 350474 | 0.017 | 60.062 |
| Platelet count | rs6880286 | 5 | 61539413 | T | C | 0.413 | 0.019 | 0.002 | 1.21E-15 | 350474 | 0.026 | 90.249 |
| Platelet count | rs11134475 | 5 | 156399950 | G | A | 0.637 | -0.018 | 0.002 | 1.49E-14 | 350474 | 0.023 | 81.000 |
| Platelet count | rs2032885 | 5 | 74613918 | G | C | 0.552 | -0.018 | 0.002 | 9.60E-14 | 350474 | 0.023 | 81.000 |
| Platelet count | rs11747856 | 5 | 60113649 | A | G | 0.794 | 0.019 | 0.003 | 4.42E-11 | 350474 | 0.011 | 40.111 |
| Platelet count | rs2246090 | 5 | 172503329 | A | T | 0.592 | 0.014 | 0.002 | 2.29E-09 | 350474 | 0.014 | 49.000 |
| Platelet count | rs114694170 | 5 | 88180196 | C | T | 0.059 | 0.144 | 0.005 | 1.11E-186 | 350474 | 0.236 | 829.435 |
| Platelet count | rs13154903 | 5 | 77636732 | C | T | 0.112 | -0.021 | 0.004 | 1.69E-08 | 350474 | 0.008 | 27.562 |
| Platelet count | rs457648 | 5 | 34658419 | T | C | 0.461 | 0.013 | 0.002 | 2.19E-08 | 350474 | 0.012 | 42.250 |
| Platelet count | rs7705526 | 5 | 1285974 | A | C | 0.325 | 0.038 | 0.002 | 2.52E-52 | 350474 | 0.103 | 360.998 |
| Platelet count | rs61215818 | 5 | 111059676 | A | G | 0.077 | -0.044 | 0.004 | 1.06E-23 | 350474 | 0.035 | 120.999 |
| Platelet count | rs10900828 | 5 | 133836294 | G | T | 0.58 | 0.017 | 0.002 | 2.82E-12 | 350474 | 0.021 | 72.250 |
| Platelet count | rs10056083 | 5 | 67226402 | C | A | 0.686 | -0.014 | 0.003 | 1.80E-08 | 350474 | 0.006 | 21.778 |
| Platelet count | rs2546979 | 5 | 159595612 | C | G | 0.702 | -0.041 | 0.003 | 8.22E-59 | 350474 | 0.053 | 186.777 |
| Platelet count | rs11950562 | 5 | 131652529 | C | A | 0.476 | -0.028 | 0.002 | 7.08E-34 | 350474 | 0.056 | 195.999 |
| Platelet count | rs249681 | 5 | 141538017 | C | G | 0.616 | 0.026 | 0.002 | 2.77E-28 | 350474 | 0.048 | 168.999 |
| Platelet count | rs34651 | 5 | 72144005 | T | C | 0.918 | 0.044 | 0.004 | 1.14E-25 | 350474 | 0.035 | 120.999 |
| Platelet count | rs35267052 | 5 | 87949118 | G | T | 0.102 | 0.029 | 0.004 | 3.61E-14 | 350474 | 0.015 | 52.562 |
| Platelet count | rs183072 | 5 | 139054033 | C | T | 0.327 | -0.015 | 0.002 | 2.90E-09 | 350474 | 0.016 | 56.250 |
| Platelet count | rs10040653 | 5 | 158229824 | T | C | 0.084 | -0.031 | 0.004 | 1.40E-13 | 350474 | 0.017 | 60.062 |
| Platelet count | rs139039269 | 5 | 1105142 | C | A | 0.069 | -0.028 | 0.005 | 9.94E-10 | 350474 | 0.009 | 31.360 |
| Platelet count | rs301371 | 6 | 25519515 | A | G | 0.409 | 0.041 | 0.002 | 7.18E-68 | 350474 | 0.120 | 420.248 |
| Platelet count | rs9357551 | 6 | 47606029 | T | C | 0.267 | 0.029 | 0.003 | 6.86E-29 | 350474 | 0.027 | 93.444 |
| Platelet count | rs1224382 | 6 | 37156606 | C | T | 0.54 | -0.019 | 0.002 | 1.95E-16 | 350474 | 0.026 | 90.249 |
| Platelet count | rs61025394 | 6 | 7085817 | A | G | 0.21 | -0.017 | 0.003 | 4.49E-09 | 350474 | 0.009 | 32.111 |
| Platelet count | rs761841 | 6 | 131114386 | C | T | 0.576 | 0.013 | 0.002 | 3.69E-08 | 350474 | 0.012 | 42.250 |
| Platelet count | rs34164109 | 6 | 135421176 | T | C | 0.262 | 0.109 | 0.003 | 1.00E-200 | 350474 | 0.375 | 1320.104 |
| Platelet count | rs9260012 | 6 | 29905900 | G | A | 0.392 | 0.028 | 0.002 | 5.36E-30 | 350474 | 0.056 | 195.999 |
| Platelet count | rs4715332 | 6 | 52669185 | A | C | 0.572 | -0.023 | 0.002 | 3.01E-22 | 350474 | 0.038 | 132.249 |
| Platelet count | rs2057149 | 6 | 110717493 | T | C | 0.649 | 0.019 | 0.002 | 1.65E-14 | 350474 | 0.026 | 90.249 |
| Platelet count | rs78157248 | 6 | 52284245 | C | T | 0.069 | 0.029 | 0.005 | 3.32E-10 | 350474 | 0.010 | 33.640 |
| Platelet count | rs2523842 | 6 | 31029282 | A | G | 0.03 | -0.041 | 0.007 | 1.87E-09 | 350474 | 0.010 | 34.306 |
| Platelet count | rs4072989 | 6 | 166140927 | C | A | 0.549 | 0.013 | 0.002 | 2.69E-08 | 350474 | 0.012 | 42.250 |
| Platelet count | rs9376060 | 6 | 135052237 | G | A | 0.22 | 0.033 | 0.003 | 5.14E-33 | 350474 | 0.035 | 120.999 |
| Platelet count | rs2818866 | 6 | 147493989 | T | C | 0.435 | 0.013 | 0.002 | 2.25E-08 | 350474 | 0.012 | 42.250 |
| Platelet count | rs9266658 | 6 | 31347644 | A | G | 0.162 | 0.06 | 0.003 | 3.39E-80 | 350474 | 0.114 | 399.998 |
| Platelet count | rs381500 | 6 | 164478388 | A | C | 0.452 | 0.027 | 0.002 | 7.53E-32 | 350474 | 0.052 | 182.249 |
| Platelet count | rs9320282 | 6 | 109619377 | G | A | 0.523 | 0.024 | 0.002 | 2.82E-25 | 350474 | 0.041 | 143.999 |
| Platelet count | rs210798 | 6 | 135514558 | G | T | 0.521 | -0.019 | 0.002 | 5.43E-17 | 350474 | 0.026 | 90.249 |
| Platelet count | rs511515 | 6 | 33541507 | G | A | 0.699 | 0.098 | 0.003 | 1.00E-200 | 350474 | 0.304 | 1067.105 |
| Platelet count | rs9274419 | 6 | 32632968 | C | G | 0.425 | -0.024 | 0.002 | 3.75E-24 | 350474 | 0.041 | 143.999 |
| Platelet count | rs13208045 | 6 | 47348288 | T | C | 0.107 | -0.031 | 0.004 | 1.09E-16 | 350474 | 0.017 | 60.062 |
| Platelet count | rs211510 | 6 | 11593985 | G | A | 0.466 | -0.014 | 0.002 | 5.13E-10 | 350474 | 0.014 | 49.000 |
| Platelet count | rs12175076 | 6 | 169663240 | C | T | 0.696 | -0.014 | 0.003 | 4.26E-08 | 350474 | 0.006 | 21.778 |
| Platelet count | rs13224082 | 7 | 116515781 | T | A | 0.242 | -0.03 | 0.003 | 1.16E-28 | 350474 | 0.029 | 99.999 |
| Platelet count | rs2015210 | 7 | 130737982 | G | T | 0.753 | -0.021 | 0.003 | 2.64E-15 | 350474 | 0.014 | 49.000 |
| Platelet count | rs62491415 | 7 | 139832617 | A | G | 0.098 | -0.025 | 0.004 | 2.93E-10 | 350474 | 0.011 | 39.062 |
| Platelet count | rs58221005 | 7 | 17458747 | A | G | 0.2 | -0.016 | 0.003 | 3.01E-08 | 350474 | 0.008 | 28.444 |
| Platelet count | rs11764390 | 7 | 80216205 | A | G | 0.438 | 0.024 | 0.002 | 3.09E-24 | 350474 | 0.041 | 143.999 |
| Platelet count | rs1154002 | 7 | 158616993 | A | G | 0.942 | -0.038 | 0.005 | 6.27E-15 | 350474 | 0.016 | 57.760 |
| Platelet count | rs4947490 | 7 | 55160538 | G | A | 0.679 | 0.016 | 0.002 | 3.00E-10 | 350474 | 0.018 | 64.000 |
| Platelet count | rs7385804 | 7 | 100235970 | A | C | 0.624 | 0.027 | 0.002 | 1.92E-30 | 350474 | 0.052 | 182.249 |
| Platelet count | rs7789916 | 7 | 14027557 | A | G | 0.713 | -0.018 | 0.003 | 1.40E-12 | 350474 | 0.010 | 36.000 |
| Platelet count | rs342293 | 7 | 106372219 | G | C | 0.459 | -0.076 | 0.002 | 1.00E-200 | 350474 | 0.410 | 1443.992 |
| Platelet count | rs77300440 | 7 | 123411910 | T | C | 0.08 | 0.074 | 0.004 | 5.99E-68 | 350474 | 0.098 | 342.248 |
| Platelet count | rs714543 | 7 | 44887076 | G | A | 0.434 | -0.03 | 0.002 | 1.43E-38 | 350474 | 0.064 | 224.999 |
| Platelet count | rs13243214 | 7 | 2817455 | T | G | 0.299 | 0.029 | 0.003 | 6.19E-30 | 350474 | 0.027 | 93.444 |
| Platelet count | rs9638749 | 7 | 18190255 | A | G | 0.213 | -0.025 | 0.003 | 1.86E-18 | 350474 | 0.020 | 69.444 |
| Platelet count | rs4389834 | 7 | 25987129 | A | G | 0.275 | -0.016 | 0.003 | 2.19E-09 | 350474 | 0.008 | 28.444 |
| Platelet count | rs42033 | 7 | 92237533 | T | A | 0.209 | -0.016 | 0.003 | 1.47E-08 | 350474 | 0.008 | 28.444 |
| Platelet count | rs73164936 | 7 | 135667802 | G | A | 0.075 | 0.035 | 0.004 | 2.26E-15 | 350474 | 0.022 | 76.562 |
| Platelet count | rs6967348 | 7 | 129247134 | A | G | 0.912 | -0.032 | 0.004 | 3.97E-15 | 350474 | 0.018 | 64.000 |
| Platelet count | rs2700936 | 7 | 36084910 | G | T | 0.459 | 0.017 | 0.002 | 1.22E-13 | 350474 | 0.021 | 72.250 |
| Platelet count | rs59543286 | 7 | 135351310 | C | A | 0.162 | -0.019 | 0.003 | 2.90E-09 | 350474 | 0.011 | 40.111 |
| Platelet count | rs10264122 | 7 | 1009337 | C | T | 0.605 | 0.014 | 0.002 | 3.85E-09 | 350474 | 0.014 | 49.000 |
| Platelet count | rs6592965 | 7 | 50427982 | A | G | 0.454 | 0.013 | 0.002 | 1.83E-08 | 350474 | 0.012 | 42.250 |
| Platelet count | rs12542032 | 8 | 121043057 | G | C | 0.28 | -0.015 | 0.003 | 2.71E-09 | 350474 | 0.007 | 25.000 |
| Platelet count | rs12676105 | 8 | 56795439 | T | C | 0.505 | -0.019 | 0.002 | 4.90E-16 | 350474 | 0.026 | 90.249 |
| Platelet count | rs55679363 | 8 | 125867834 | T | A | 0.317 | 0.017 | 0.002 | 2.31E-12 | 350474 | 0.021 | 72.250 |
| Platelet count | rs2915607 | 8 | 30279916 | C | T | 0.742 | 0.018 | 0.003 | 3.44E-12 | 350474 | 0.010 | 36.000 |
| Platelet count | rs4841600 | 8 | 11688148 | C | T | 0.544 | -0.013 | 0.002 | 8.60E-09 | 350474 | 0.012 | 42.250 |
| Platelet count | rs7000170 | 8 | 142322896 | A | G | 0.29 | -0.018 | 0.003 | 2.03E-12 | 350474 | 0.010 | 36.000 |
| Platelet count | rs4921997 | 8 | 19013568 | T | G | 0.695 | 0.015 | 0.003 | 2.43E-09 | 350474 | 0.007 | 25.000 |
| Platelet count | rs6998007 | 8 | 40045119 | T | C | 0.566 | 0.014 | 0.002 | 6.45E-09 | 350474 | 0.014 | 49.000 |
| Platelet count | rs7833924 | 8 | 144996029 | G | A | 0.43 | 0.041 | 0.002 | 5.14E-69 | 350474 | 0.120 | 420.248 |
| Platelet count | rs28538623 | 8 | 130484346 | C | G | 0.331 | 0.019 | 0.002 | 4.30E-14 | 350474 | 0.026 | 90.249 |
| Platelet count | rs16932411 | 8 | 66922465 | G | A | 0.316 | -0.018 | 0.002 | 4.76E-13 | 350474 | 0.023 | 81.000 |
| Platelet count | rs6993770 | 8 | 106581528 | T | A | 0.286 | -0.066 | 0.003 | 1.30E-147 | 350474 | 0.138 | 483.997 |
| Platelet count | rs11779638 | 8 | 22440208 | C | A | 0.234 | 0.023 | 0.003 | 1.31E-17 | 350474 | 0.017 | 58.777 |
| Platelet count | rs113443561 | 8 | 103411756 | T | C | 0.152 | 0.019 | 0.003 | 1.75E-09 | 350474 | 0.011 | 40.111 |
| Platelet count | rs77338166 | 8 | 105469502 | G | A | 0.066 | -0.026 | 0.005 | 2.16E-08 | 350474 | 0.008 | 27.040 |
| Platelet count | rs9693128 | 8 | 82706128 | G | A | 0.609 | 0.013 | 0.002 | 3.81E-08 | 350474 | 0.012 | 42.250 |
| Platelet count | rs385893 | 9 | 4763176 | C | T | 0.526 | 0.097 | 0.002 | 1.00E-200 | 350474 | 0.667 | 2352.237 |
| Platelet count | rs114968084 | 9 | 136022954 | A | G | 0.03 | -0.079 | 0.007 | 4.00E-29 | 350474 | 0.036 | 127.367 |
| Platelet count | rs12343705 | 9 | 38197187 | T | A | 0.491 | -0.025 | 0.002 | 3.44E-26 | 350474 | 0.045 | 156.249 |
| Platelet count | rs2017536 | 9 | 123496234 | T | G | 0.696 | 0.022 | 0.003 | 3.76E-18 | 350474 | 0.015 | 53.777 |
| Platelet count | rs12343129 | 9 | 116345565 | A | C | 0.095 | 0.023 | 0.004 | 6.82E-09 | 350474 | 0.009 | 33.062 |
| Platelet count | rs7860776 | 9 | 4836566 | A | G | 0.291 | -0.033 | 0.003 | 8.65E-37 | 350474 | 0.035 | 120.999 |
| Platelet count | rs12000252 | 9 | 73037963 | G | A | 0.55 | -0.017 | 0.002 | 4.65E-13 | 350474 | 0.021 | 72.250 |
| Platelet count | rs10820606 | 9 | 99192919 | C | A | 0.228 | 0.048 | 0.003 | 4.42E-67 | 350474 | 0.073 | 255.999 |
| Platelet count | rs12376511 | 9 | 22142756 | C | T | 0.16 | -0.046 | 0.003 | 7.03E-48 | 350474 | 0.067 | 235.110 |
| Platelet count | rs59602331 | 9 | 276451 | C | T | 0.252 | -0.028 | 0.003 | 1.24E-25 | 350474 | 0.025 | 87.111 |
| Platelet count | rs3901421 | 9 | 96204538 | C | G | 0.488 | -0.014 | 0.002 | 8.24E-10 | 350474 | 0.014 | 49.000 |
| Platelet count | rs943501 | 9 | 93915270 | T | C | 0.928 | 0.027 | 0.004 | 2.64E-09 | 350474 | 0.013 | 45.562 |
| Platelet count | rs7036656 | 9 | 21990457 | T | C | 0.722 | 0.041 | 0.003 | 1.31E-57 | 350474 | 0.053 | 186.777 |
| Platelet count | rs7033052 | 9 | 5042981 | C | G | 0.531 | -0.033 | 0.002 | 2.05E-45 | 350474 | 0.078 | 272.248 |
| Platelet count | rs408307 | 9 | 136892523 | T | C | 0.264 | 0.03 | 0.003 | 1.05E-30 | 350474 | 0.029 | 99.999 |
| Platelet count | rs684562 | 9 | 35060302 | C | T | 0.719 | 0.017 | 0.003 | 2.99E-11 | 350474 | 0.009 | 32.111 |
| Platelet count | rs10117872 | 9 | 4888355 | T | C | 0.008 | -0.083 | 0.013 | 7.77E-11 | 350474 | 0.012 | 40.763 |
| Platelet count | rs60757417 | 9 | 135864436 | G | C | 0.06 | -0.081 | 0.005 | 2.38E-61 | 350474 | 0.075 | 262.439 |
| Platelet count | rs296852 | 9 | 4787167 | A | G | 0.635 | -0.039 | 0.002 | 2.68E-58 | 350474 | 0.108 | 380.248 |
| Platelet count | rs62549698 | 9 | 91396592 | T | G | 0.062 | -0.057 | 0.005 | 1.26E-32 | 350474 | 0.037 | 129.959 |
| Platelet count | rs117119759 | 9 | 136212168 | A | G | 0.029 | -0.051 | 0.007 | 6.57E-13 | 350474 | 0.015 | 53.081 |
| Platelet count | rs10817007 | 9 | 113155247 | G | T | 0.129 | 0.025 | 0.003 | 8.11E-13 | 350474 | 0.020 | 69.444 |
| Platelet count | rs760402 | 9 | 35686376 | A | G | 0.688 | -0.018 | 0.002 | 1.09E-12 | 350474 | 0.023 | 81.000 |
| Platelet count | rs12767683 | 10 | 104316581 | A | C | 0.325 | 0.018 | 0.002 | 1.06E-13 | 350474 | 0.023 | 81.000 |
| Platelet count | rs10458728 | 10 | 101323976 | C | T | 0.261 | -0.018 | 0.003 | 2.86E-12 | 350474 | 0.010 | 36.000 |
| Platelet count | rs2767627 | 10 | 30251648 | A | C | 0.453 | 0.014 | 0.002 | 6.15E-09 | 350474 | 0.014 | 49.000 |
| Platelet count | rs116052829 | 10 | 81164146 | T | C | 0.103 | 0.032 | 0.004 | 1.86E-17 | 350474 | 0.018 | 64.000 |
| Platelet count | rs11259186 | 10 | 14603429 | G | T | 0.54 | -0.017 | 0.002 | 6.43E-13 | 350474 | 0.021 | 72.250 |
| Platelet count | rs1340837 | 10 | 97542035 | G | A | 0.413 | 0.015 | 0.002 | 6.19E-10 | 350474 | 0.016 | 56.250 |
| Platelet count | rs4747508 | 10 | 25222094 | T | C | 0.583 | -0.021 | 0.002 | 6.35E-19 | 350474 | 0.031 | 110.249 |
| Platelet count | rs224082 | 10 | 64566258 | T | C | 0.376 | -0.017 | 0.002 | 3.01E-13 | 350474 | 0.021 | 72.250 |
| Platelet count | rs34346558 | 10 | 11904834 | C | A | 0.205 | 0.019 | 0.003 | 8.77E-11 | 350474 | 0.011 | 40.111 |
| Platelet count | rs10764672 | 10 | 27392541 | A | C | 0.809 | 0.017 | 0.003 | 2.75E-09 | 350474 | 0.009 | 32.111 |
| Platelet count | rs7098181 | 10 | 65027143 | T | G | 0.417 | 0.074 | 0.002 | 1.00E-200 | 350474 | 0.389 | 1368.992 |
| Platelet count | rs28505677 | 10 | 50259007 | G | C | 0.237 | -0.032 | 0.003 | 7.45E-31 | 350474 | 0.032 | 113.777 |
| Platelet count | rs2068888 | 10 | 94839642 | A | G | 0.449 | -0.021 | 0.002 | 1.28E-19 | 350474 | 0.031 | 110.249 |
| Platelet count | rs10886430 | 10 | 121010256 | G | A | 0.127 | -0.03 | 0.004 | 7.82E-17 | 350474 | 0.016 | 56.250 |
| Platelet count | rs4980067 | 10 | 81136129 | A | C | 0.504 | 0.018 | 0.002 | 8.00E-15 | 350474 | 0.023 | 81.000 |
| Platelet count | rs7912035 | 10 | 3707315 | C | T | 0.857 | 0.025 | 0.003 | 7.96E-14 | 350474 | 0.020 | 69.444 |
| Platelet count | rs725529 | 10 | 63814070 | T | A | 0.369 | 0.015 | 0.002 | 2.89E-10 | 350474 | 0.016 | 56.250 |
| Platelet count | rs7080838 | 10 | 126419841 | T | C | 0.568 | 0.014 | 0.002 | 9.55E-10 | 350474 | 0.014 | 49.000 |
| Platelet count | rs2066334 | 10 | 30323189 | G | A | 0.406 | -0.013 | 0.002 | 1.22E-08 | 350474 | 0.012 | 42.250 |
| Platelet count | rs7103603 | 11 | 128297998 | A | G | 0.39 | -0.029 | 0.002 | 1.29E-32 | 350474 | 0.060 | 210.249 |
| Platelet count | rs55775277 | 11 | 65457575 | G | T | 0.359 | -0.026 | 0.002 | 4.00E-27 | 350474 | 0.048 | 168.999 |
| Platelet count | rs10769981 | 11 | 9024460 | C | T | 0.456 | -0.024 | 0.002 | 5.50E-26 | 350474 | 0.041 | 143.999 |
| Platelet count | rs58405523 | 11 | 47918362 | T | A | 0.16 | 0.028 | 0.003 | 1.70E-18 | 350474 | 0.025 | 87.111 |
| Platelet count | rs10832728 | 11 | 17070581 | G | A | 0.523 | -0.018 | 0.002 | 1.53E-14 | 350474 | 0.023 | 81.000 |
| Platelet count | rs28456 | 11 | 61589481 | G | A | 0.315 | 0.04 | 0.002 | 2.28E-59 | 350474 | 0.114 | 399.998 |
| Platelet count | rs28362951 | 11 | 57372526 | G | A | 0.267 | 0.023 | 0.003 | 3.89E-19 | 350474 | 0.017 | 58.777 |
| Platelet count | rs56133113 | 11 | 72501220 | G | A | 0.119 | 0.02 | 0.004 | 3.87E-08 | 350474 | 0.007 | 25.000 |
| Platelet count | rs7940646 | 11 | 10669228 | C | T | 0.69 | 0.024 | 0.002 | 2.26E-21 | 350474 | 0.041 | 143.999 |
| Platelet count | rs2282492 | 11 | 63918589 | C | T | 0.377 | -0.019 | 0.002 | 1.11E-15 | 350474 | 0.026 | 90.249 |
| Platelet count | rs556562 | 11 | 85666440 | C | A | 0.808 | 0.023 | 0.003 | 4.84E-15 | 350474 | 0.017 | 58.777 |
| Platelet count | rs11601507 | 11 | 5701074 | A | C | 0.068 | -0.033 | 0.005 | 1.45E-13 | 350474 | 0.012 | 43.560 |
| Platelet count | rs10750385 | 11 | 127937766 | G | A | 0.672 | 0.016 | 0.002 | 4.82E-11 | 350474 | 0.018 | 64.000 |
| Platelet count | rs183811190 | 11 | 32861405 | T | A | 0.036 | -0.04 | 0.006 | 1.91E-10 | 350474 | 0.013 | 44.444 |
| Platelet count | rs7123132 | 11 | 44598235 | G | A | 0.543 | 0.014 | 0.002 | 4.27E-10 | 350474 | 0.014 | 49.000 |
| Platelet count | rs10792079 | 11 | 57010414 | A | C | 0.57 | -0.013 | 0.002 | 1.57E-08 | 350474 | 0.012 | 42.250 |
| Platelet count | rs11604127 | 11 | 196944 | T | C | 0.232 | 0.092 | 0.003 | 1.00E-200 | 350474 | 0.268 | 940.439 |
| Platelet count | rs2155311 | 11 | 126291475 | A | G | 0.481 | -0.027 | 0.002 | 9.70E-31 | 350474 | 0.052 | 182.249 |
| Platelet count | rs964184 | 11 | 116648917 | C | G | 0.868 | 0.039 | 0.003 | 1.19E-29 | 350474 | 0.048 | 168.999 |
| Platelet count | rs55928935 | 11 | 113999364 | T | G | 0.116 | -0.025 | 0.004 | 2.27E-12 | 350474 | 0.011 | 39.062 |
| Platelet count | rs4936066 | 11 | 128584765 | C | A | 0.19 | -0.019 | 0.003 | 4.30E-11 | 350474 | 0.011 | 40.111 |
| Platelet count | rs2155380 | 11 | 119080037 | G | A | 0.271 | 0.054 | 0.003 | 6.23E-94 | 350474 | 0.092 | 323.998 |
| Platelet count | rs7934719 | 11 | 108341864 | T | C | 0.409 | 0.021 | 0.002 | 1.10E-19 | 350474 | 0.031 | 110.249 |
| Platelet count | rs7127210 | 11 | 77704282 | T | G | 0.315 | 0.019 | 0.003 | 8.08E-14 | 350474 | 0.011 | 40.111 |
| Platelet count | rs640221 | 11 | 94832645 | G | A | 0.834 | 0.017 | 0.003 | 1.77E-08 | 350474 | 0.009 | 32.111 |
| Platelet count | rs10876550 | 12 | 54712308 | A | G | 0.559 | 0.052 | 0.002 | 4.86E-111 | 350474 | 0.193 | 675.996 |
| Platelet count | rs7973618 | 12 | 57019443 | C | T | 0.366 | 0.043 | 0.002 | 1.69E-70 | 350474 | 0.132 | 462.247 |
| Platelet count | rs12425835 | 12 | 93903228 | T | C | 0.159 | 0.026 | 0.003 | 1.10E-16 | 350474 | 0.021 | 75.111 |
| Platelet count | rs111893804 | 12 | 6509886 | G | A | 0.5 | -0.02 | 0.002 | 5.36E-16 | 350474 | 0.029 | 99.999 |
| Platelet count | rs2122403 | 12 | 80347212 | A | G | 0.921 | -0.031 | 0.004 | 1.09E-12 | 350474 | 0.017 | 60.062 |
| Platelet count | rs55633676 | 12 | 6194991 | C | T | 0.156 | 0.018 | 0.003 | 3.11E-08 | 350474 | 0.010 | 36.000 |
| Platelet count | rs3184504 | 12 | 111884608 | C | T | 0.518 | -0.096 | 0.002 | 1.00E-200 | 350474 | 0.653 | 2303.987 |
| Platelet count | rs2015599 | 12 | 29435480 | A | G | 0.46 | -0.044 | 0.002 | 3.18E-80 | 350474 | 0.138 | 483.997 |
| Platelet count | rs78689302 | 12 | 65033149 | G | T | 0.101 | 0.051 | 0.004 | 3.59E-40 | 350474 | 0.046 | 162.562 |
| Platelet count | rs73109811 | 12 | 48212719 | T | C | 0.199 | 0.032 | 0.003 | 4.01E-28 | 350474 | 0.032 | 113.777 |
| Platelet count | rs12824685 | 12 | 123817569 | T | G | 0.204 | -0.03 | 0.003 | 9.25E-25 | 350474 | 0.029 | 99.999 |
| Platelet count | rs1558328 | 12 | 6288834 | T | C | 0.497 | -0.052 | 0.002 | 1.94E-111 | 350474 | 0.193 | 675.996 |
| Platelet count | rs10849333 | 12 | 719311 | T | G | 0.276 | -0.024 | 0.003 | 3.96E-20 | 350474 | 0.018 | 64.000 |
| Platelet count | rs11107880 | 12 | 95452854 | A | G | 0.069 | 0.026 | 0.005 | 1.51E-08 | 350474 | 0.008 | 27.040 |
| Platelet count | rs3742028 | 12 | 109491468 | A | G | 0.582 | -0.04 | 0.002 | 8.10E-66 | 350474 | 0.114 | 399.998 |
| Platelet count | rs17860346 | 12 | 51726824 | T | C | 0.106 | 0.039 | 0.004 | 1.91E-25 | 350474 | 0.027 | 95.062 |
| Platelet count | rs2255531 | 12 | 121414915 | A | G | 0.348 | -0.023 | 0.002 | 1.14E-20 | 350474 | 0.038 | 132.249 |
| Platelet count | rs7966357 | 12 | 51209838 | G | C | 0.36 | 0.019 | 0.002 | 6.13E-15 | 350474 | 0.026 | 90.249 |
| Platelet count | rs1436367 | 12 | 64715216 | C | A | 0.223 | -0.018 | 0.003 | 1.40E-10 | 350474 | 0.010 | 36.000 |
| Platelet count | rs1035317 | 12 | 109195024 | G | A | 0.388 | 0.015 | 0.002 | 2.38E-10 | 350474 | 0.016 | 56.250 |
| Platelet count | rs7132697 | 12 | 68532841 | A | T | 0.702 | 0.014 | 0.003 | 2.01E-08 | 350474 | 0.006 | 21.778 |
| Platelet count | rs11553699 | 12 | 122216910 | G | A | 0.135 | -0.11 | 0.004 | 1.00E-200 | 350474 | 0.215 | 756.246 |
| Platelet count | rs7974597 | 12 | 49631531 | G | A | 0.076 | -0.047 | 0.004 | 1.01E-26 | 350474 | 0.039 | 138.062 |
| Platelet count | rs113825134 | 12 | 78220740 | A | G | 0.234 | -0.018 | 0.003 | 2.93E-11 | 350474 | 0.010 | 36.000 |
| Platelet count | rs670179 | 13 | 71236607 | A | T | 0.574 | 0.021 | 0.002 | 1.26E-18 | 350474 | 0.031 | 110.249 |
| Platelet count | rs303968 | 13 | 72340143 | C | T | 0.398 | -0.016 | 0.002 | 4.42E-11 | 350474 | 0.018 | 64.000 |
| Platelet count | rs4771666 | 13 | 110999252 | T | C | 0.235 | 0.015 | 0.003 | 3.02E-08 | 350474 | 0.007 | 25.000 |
| Platelet count | rs11841319 | 13 | 110492626 | T | C | 0.103 | -0.061 | 0.004 | 1.14E-57 | 350474 | 0.066 | 232.561 |
| Platelet count | rs9532658 | 13 | 41494245 | C | A | 0.685 | 0.022 | 0.002 | 1.44E-19 | 350474 | 0.035 | 120.999 |
| Platelet count | rs9591137 | 13 | 33152482 | C | T | 0.383 | 0.023 | 0.002 | 2.27E-22 | 350474 | 0.038 | 132.249 |
| Platelet count | rs374039502 | 13 | 108960385 | A | T | 0.021 | -0.049 | 0.009 | 8.69E-09 | 350474 | 0.008 | 29.642 |
| Platelet count | rs66527945 | 13 | 114075135 | T | C | 0.069 | 0.026 | 0.005 | 1.80E-08 | 350474 | 0.008 | 27.040 |
| Platelet count | rs3956381 | 13 | 95972874 | A | C | 0.58 | 0.017 | 0.002 | 2.37E-12 | 350474 | 0.021 | 72.250 |
| Platelet count | rs4148445 | 13 | 95897359 | T | C | 0.918 | 0.064 | 0.004 | 3.74E-53 | 350474 | 0.073 | 255.999 |
| Platelet count | rs11164132 | 13 | 114018953 | C | T | 0.357 | 0.03 | 0.002 | 3.21E-35 | 350474 | 0.064 | 224.999 |
| Platelet count | rs9535492 | 13 | 51384616 | T | C | 0.564 | -0.015 | 0.002 | 2.69E-10 | 350474 | 0.016 | 56.250 |
| Platelet count | rs2297066 | 14 | 103566835 | G | C | 0.242 | 0.052 | 0.003 | 2.57E-82 | 350474 | 0.086 | 300.443 |
| Platelet count | rs72725173 | 14 | 68509589 | A | G | 0.157 | -0.043 | 0.003 | 6.54E-42 | 350474 | 0.059 | 205.443 |
| Platelet count | rs2934701 | 14 | 51133287 | T | A | 0.427 | -0.017 | 0.002 | 4.64E-13 | 350474 | 0.021 | 72.250 |
| Platelet count | rs4083463 | 14 | 81856323 | A | G | 0.335 | -0.017 | 0.002 | 4.17E-12 | 350474 | 0.021 | 72.250 |
| Platelet count | rs947001 | 14 | 104638441 | G | A | 0.673 | -0.015 | 0.002 | 1.19E-09 | 350474 | 0.016 | 56.250 |
| Platelet count | rs112879674 | 14 | 55420273 | A | G | 0.437 | 0.014 | 0.002 | 2.02E-09 | 350474 | 0.014 | 49.000 |
| Platelet count | rs17127600 | 14 | 92279983 | G | T | 0.172 | 0.017 | 0.003 | 1.81E-08 | 350474 | 0.009 | 32.111 |
| Platelet count | rs7142089 | 14 | 101172229 | A | T | 0.217 | -0.049 | 0.003 | 1.53E-61 | 350474 | 0.076 | 266.776 |
| Platelet count | rs34750619 | 14 | 93514882 | A | G | 0.07 | 0.037 | 0.005 | 3.99E-16 | 350474 | 0.016 | 54.760 |
| Platelet count | rs178761 | 14 | 23702056 | G | A | 0.285 | -0.016 | 0.003 | 1.67E-10 | 350474 | 0.008 | 28.444 |
| Platelet count | rs10145353 | 14 | 96204459 | G | A | 0.17 | -0.018 | 0.003 | 1.08E-08 | 350474 | 0.010 | 36.000 |
| Platelet count | rs35258120 | 14 | 103058769 | A | G | 0.819 | 0.046 | 0.003 | 1.12E-53 | 350474 | 0.067 | 235.110 |
| Platelet count | rs11627485 | 14 | 65487694 | C | T | 0.45 | 0.025 | 0.002 | 1.38E-27 | 350474 | 0.045 | 156.249 |
| Platelet count | rs12147994 | 14 | 65751627 | A | C | 0.26 | -0.022 | 0.003 | 5.94E-16 | 350474 | 0.015 | 53.777 |
| Platelet count | rs10047928 | 14 | 31368063 | A | G | 0.254 | -0.018 | 0.003 | 5.47E-12 | 350474 | 0.010 | 36.000 |
| Platelet count | rs10220411 | 14 | 69452088 | G | A | 0.262 | 0.028 | 0.003 | 3.51E-27 | 350474 | 0.025 | 87.111 |
| Platelet count | rs11157951 | 14 | 53599024 | A | G | 0.833 | 0.02 | 0.003 | 1.27E-10 | 350474 | 0.013 | 44.444 |
| Platelet count | rs1519819 | 15 | 78541769 | T | C | 0.608 | 0.015 | 0.002 | 5.19E-10 | 350474 | 0.016 | 56.250 |
| Platelet count | rs147233090 | 15 | 44028047 | T | C | 0.024 | 0.108 | 0.008 | 7.24E-46 | 350474 | 0.052 | 182.249 |
| Platelet count | rs4966015 | 15 | 99248132 | T | G | 0.144 | -0.035 | 0.003 | 5.11E-27 | 350474 | 0.039 | 136.110 |
| Platelet count | rs1982101 | 15 | 39300917 | T | A | 0.393 | -0.019 | 0.002 | 1.37E-15 | 350474 | 0.026 | 90.249 |
| Platelet count | rs8028182 | 15 | 75718669 | T | G | 0.193 | -0.021 | 0.003 | 1.22E-12 | 350474 | 0.014 | 49.000 |
| Platelet count | rs12903325 | 15 | 50353277 | G | T | 0.241 | 0.019 | 0.003 | 2.61E-12 | 350474 | 0.011 | 40.111 |
| Platelet count | rs9919955 | 15 | 57083831 | C | G | 0.163 | -0.027 | 0.003 | 4.43E-18 | 350474 | 0.023 | 81.000 |
| Platelet count | rs17687755 | 15 | 42229838 | C | G | 0.289 | 0.018 | 0.003 | 2.57E-12 | 350474 | 0.010 | 36.000 |
| Platelet count | rs11071720 | 15 | 63341996 | C | T | 0.702 | 0.038 | 0.003 | 1.46E-51 | 350474 | 0.046 | 160.444 |
| Platelet count | rs61009920 | 15 | 65181280 | A | C | 0.137 | 0.046 | 0.003 | 3.53E-43 | 350474 | 0.067 | 235.110 |
| Platelet count | rs2301825 | 15 | 91528070 | G | C | 0.129 | -0.031 | 0.003 | 7.09E-19 | 350474 | 0.030 | 106.777 |
| Platelet count | rs11073731 | 15 | 85355113 | A | C | 0.448 | 0.015 | 0.002 | 1.78E-10 | 350474 | 0.016 | 56.250 |
| Platelet count | rs113721422 | 16 | 530720 | A | G | 0.079 | 0.049 | 0.004 | 1.07E-29 | 350474 | 0.043 | 150.062 |
| Platelet count | rs4888387 | 16 | 75389755 | G | T | 0.595 | 0.017 | 0.002 | 3.05E-13 | 350474 | 0.021 | 72.250 |
| Platelet count | rs72807421 | 16 | 88590467 | T | C | 0.026 | -0.053 | 0.008 | 2.28E-12 | 350474 | 0.013 | 43.890 |
| Platelet count | rs12932413 | 16 | 4650551 | T | C | 0.161 | 0.019 | 0.003 | 1.93E-09 | 350474 | 0.011 | 40.111 |
| Platelet count | rs62048972 | 16 | 88556007 | T | C | 0.164 | 0.039 | 0.003 | 1.22E-36 | 350474 | 0.048 | 168.999 |
| Platelet count | rs4783183 | 16 | 85415465 | G | C | 0.876 | -0.043 | 0.004 | 3.85E-34 | 350474 | 0.033 | 115.562 |
| Platelet count | rs183725 | 16 | 9048035 | C | T | 0.773 | 0.026 | 0.003 | 1.88E-19 | 350474 | 0.021 | 75.111 |
| Platelet count | rs11075750 | 16 | 53154361 | G | A | 0.399 | 0.017 | 0.002 | 3.14E-13 | 350474 | 0.021 | 72.250 |
| Platelet count | rs9927293 | 16 | 5020954 | A | C | 0.46 | -0.016 | 0.002 | 1.33E-11 | 350474 | 0.018 | 64.000 |
| Platelet count | rs4785386 | 16 | 50164049 | A | G | 0.751 | 0.016 | 0.003 | 2.74E-09 | 350474 | 0.008 | 28.444 |
| Platelet count | rs12149202 | 16 | 85700360 | A | G | 0.276 | 0.022 | 0.003 | 3.86E-17 | 350474 | 0.015 | 53.777 |
| Platelet count | rs11648192 | 16 | 28618708 | T | C | 0.435 | -0.014 | 0.002 | 4.05E-09 | 350474 | 0.014 | 49.000 |
| Platelet count | rs151234 | 16 | 28505660 | C | G | 0.129 | 0.065 | 0.003 | 3.84E-78 | 350474 | 0.134 | 469.442 |
| Platelet count | rs11150621 | 16 | 31406756 | T | C | 0.357 | 0.014 | 0.002 | 4.03E-09 | 350474 | 0.014 | 49.000 |
| Platelet count | rs74505413 | 17 | 33923552 | G | T | 0.043 | 0.094 | 0.006 | 1.10E-59 | 350474 | 0.070 | 245.443 |
| Platelet count | rs8178824 | 17 | 64224775 | T | C | 0.03 | 0.089 | 0.007 | 3.13E-39 | 350474 | 0.046 | 161.652 |
| Platelet count | rs4061659 | 17 | 1997444 | C | G | 0.58 | -0.029 | 0.002 | 3.95E-35 | 350474 | 0.060 | 210.249 |
| Platelet count | rs853195 | 17 | 35848255 | A | C | 0.637 | -0.02 | 0.002 | 3.11E-17 | 350474 | 0.029 | 99.999 |
| Platelet count | rs9895429 | 17 | 57496970 | C | A | 0.236 | -0.022 | 0.003 | 1.03E-15 | 350474 | 0.015 | 53.777 |
| Platelet count | rs2084312 | 17 | 72695211 | T | C | 0.8 | 0.018 | 0.003 | 2.26E-10 | 350474 | 0.010 | 36.000 |
| Platelet count | rs6505129 | 17 | 27753618 | A | G | 0.517 | -0.068 | 0.002 | 9.64E-192 | 350474 | 0.329 | 1155.993 |
| Platelet count | rs4531775 | 17 | 33886938 | A | C | 0.087 | 0.053 | 0.004 | 6.44E-38 | 350474 | 0.050 | 175.561 |
| Platelet count | rs183583130 | 17 | 4942516 | T | G | 0.024 | -0.061 | 0.008 | 1.01E-14 | 350474 | 0.017 | 58.140 |
| Platelet count | rs36034566 | 17 | 19911649 | C | A | 0.483 | 0.018 | 0.002 | 2.57E-14 | 350474 | 0.023 | 81.000 |
| Platelet count | rs242554 | 17 | 44039365 | T | C | 0.413 | -0.016 | 0.002 | 1.01E-11 | 350474 | 0.018 | 64.000 |
| Platelet count | rs112951098 | 17 | 46563393 | T | C | 0.158 | -0.021 | 0.003 | 6.42E-11 | 350474 | 0.014 | 49.000 |
| Platelet count | rs12451471 | 17 | 78102517 | T | C | 0.374 | -0.014 | 0.002 | 3.58E-09 | 350474 | 0.014 | 49.000 |
| Platelet count | rs2641444 | 17 | 2037402 | C | T | 0.286 | -0.025 | 0.003 | 6.23E-22 | 350474 | 0.020 | 69.444 |
| Platelet count | rs12945043 | 17 | 16896277 | G | A | 0.434 | -0.015 | 0.002 | 3.93E-10 | 350474 | 0.016 | 56.250 |
| Platelet count | rs2241233 | 17 | 7318396 | T | C | 0.861 | -0.02 | 0.003 | 2.74E-09 | 350474 | 0.013 | 44.444 |
| Platelet count | rs2243103 | 17 | 4839400 | G | C | 0.073 | 0.07 | 0.004 | 1.64E-56 | 350474 | 0.087 | 306.248 |
| Platelet count | rs9910058 | 17 | 76144314 | A | G | 0.54 | -0.014 | 0.002 | 1.38E-09 | 350474 | 0.014 | 49.000 |
| Platelet count | rs1865761 | 18 | 67539392 | C | T | 0.523 | -0.017 | 0.002 | 2.32E-13 | 350474 | 0.021 | 72.250 |
| Platelet count | rs8091707 | 18 | 22465537 | C | A | 0.087 | 0.026 | 0.004 | 1.11E-10 | 350474 | 0.012 | 42.250 |
| Platelet count | rs79010100 | 18 | 42005538 | C | T | 0.131 | 0.026 | 0.003 | 6.00E-14 | 350474 | 0.021 | 75.111 |
| Platelet count | rs632157 | 18 | 72911548 | A | G | 0.065 | -0.035 | 0.005 | 7.82E-14 | 350474 | 0.014 | 49.000 |
| Platelet count | rs7233932 | 18 | 812713 | G | C | 0.319 | 0.017 | 0.002 | 2.74E-11 | 350474 | 0.021 | 72.250 |
| Platelet count | rs11082304 | 18 | 20720973 | T | G | 0.514 | -0.052 | 0.002 | 9.77E-113 | 350474 | 0.193 | 675.996 |
| Platelet count | rs583306 | 18 | 9617742 | T | C | 0.738 | 0.023 | 0.003 | 2.78E-18 | 350474 | 0.017 | 58.777 |
| Platelet count | rs17758695 | 18 | 60920854 | T | C | 0.03 | -0.053 | 0.007 | 4.97E-15 | 350474 | 0.016 | 57.326 |
| Platelet count | rs72969820 | 18 | 57345190 | T | C | 0.115 | -0.028 | 0.004 | 3.81E-14 | 350474 | 0.014 | 49.000 |
| Platelet count | rs11667430 | 19 | 45716197 | G | A | 0.395 | -0.041 | 0.002 | 3.80E-67 | 350474 | 0.120 | 420.248 |
| Platelet count | rs7252328 | 19 | 2003785 | G | A | 0.153 | 0.023 | 0.003 | 2.42E-12 | 350474 | 0.017 | 58.777 |
| Platelet count | rs4802252 | 19 | 45904759 | C | T | 0.387 | -0.016 | 0.002 | 3.58E-12 | 350474 | 0.018 | 64.000 |
| Platelet count | rs11879091 | 19 | 1082265 | T | C | 0.518 | -0.015 | 0.002 | 3.08E-10 | 350474 | 0.016 | 56.250 |
| Platelet count | rs7254827 | 19 | 17202746 | T | A | 0.225 | -0.032 | 0.003 | 5.11E-31 | 350474 | 0.032 | 113.777 |
| Platelet count | rs72999007 | 19 | 19328357 | C | T | 0.011 | 0.122 | 0.011 | 1.30E-26 | 350474 | 0.035 | 123.008 |
| Platelet count | rs62126620 | 19 | 33753200 | A | G | 0.195 | 0.019 | 0.003 | 2.48E-10 | 350474 | 0.011 | 40.111 |
| Platelet count | rs12459419 | 19 | 51728477 | T | C | 0.326 | -0.022 | 0.002 | 2.62E-18 | 350474 | 0.035 | 120.999 |
| Platelet count | rs7253820 | 19 | 38726076 | G | A | 0.468 | 0.02 | 0.002 | 5.46E-18 | 350474 | 0.029 | 99.999 |
| Platelet count | rs9304813 | 19 | 58931557 | G | A | 0.484 | 0.02 | 0.002 | 9.14E-18 | 350474 | 0.029 | 99.999 |
| Platelet count | rs11882796 | 19 | 49225766 | T | A | 0.544 | 0.018 | 0.002 | 3.10E-15 | 350474 | 0.023 | 81.000 |
| Platelet count | rs112671186 | 19 | 12787701 | A | G | 0.027 | -0.043 | 0.007 | 9.19E-09 | 350474 | 0.011 | 37.734 |
| Platelet count | rs57843631 | 19 | 16206012 | T | C | 0.019 | -0.225 | 0.009 | 1.44E-147 | 350474 | 0.178 | 624.996 |
| Platelet count | rs34536443 | 19 | 10463118 | C | G | 0.046 | -0.055 | 0.006 | 2.47E-22 | 350474 | 0.024 | 84.027 |
| Platelet count | rs8106212 | 19 | 6802571 | T | C | 0.012 | -0.086 | 0.011 | 1.59E-15 | 350474 | 0.017 | 61.124 |
| Platelet count | rs78400579 | 19 | 39178653 | A | G | 0.059 | 0.053 | 0.005 | 2.18E-26 | 350474 | 0.032 | 112.359 |
| Platelet count | rs8108355 | 19 | 35663736 | A | G | 0.717 | -0.017 | 0.003 | 5.31E-11 | 350474 | 0.009 | 32.111 |
| Platelet count | rs59192911 | 19 | 2129029 | A | C | 0.235 | 0.017 | 0.003 | 3.81E-09 | 350474 | 0.009 | 32.111 |
| Platelet count | rs16979901 | 20 | 54988877 | G | A | 0.102 | 0.032 | 0.004 | 7.79E-17 | 350474 | 0.018 | 64.000 |
| Platelet count | rs6026699 | 20 | 57674276 | A | T | 0.28 | 0.018 | 0.003 | 1.48E-11 | 350474 | 0.010 | 36.000 |
| Platelet count | rs4812449 | 20 | 39272739 | G | C | 0.407 | 0.014 | 0.002 | 2.89E-09 | 350474 | 0.014 | 49.000 |
| Platelet count | rs6103669 | 20 | 42816845 | A | G | 0.287 | -0.019 | 0.003 | 3.36E-14 | 350474 | 0.011 | 40.111 |
| Platelet count | rs4809330 | 20 | 62349586 | G | A | 0.666 | 0.017 | 0.002 | 4.44E-12 | 350474 | 0.021 | 72.250 |
| Platelet count | rs6060978 | 20 | 30416893 | A | G | 0.216 | -0.025 | 0.003 | 1.92E-19 | 350474 | 0.020 | 69.444 |
| Platelet count | rs80054178 | 20 | 30294682 | C | T | 0.022 | 0.122 | 0.008 | 2.11E-55 | 350474 | 0.066 | 232.561 |
| Platelet count | rs4630833 | 20 | 1930798 | C | T | 0.261 | -0.033 | 0.003 | 1.12E-36 | 350474 | 0.035 | 120.999 |
| Platelet count | rs149142833 | 20 | 32188142 | T | C | 0.156 | 0.021 | 0.003 | 1.12E-10 | 350474 | 0.014 | 49.000 |
| Platelet count | rs1883932 | 20 | 8609588 | T | A | 0.508 | -0.021 | 0.002 | 8.19E-19 | 350474 | 0.031 | 110.249 |
| Platelet count | rs1984021 | 21 | 39836204 | A | G | 0.136 | 0.03 | 0.003 | 1.86E-19 | 350474 | 0.029 | 99.999 |
| Platelet count | rs55773542 | 21 | 36368272 | T | C | 0.105 | -0.025 | 0.004 | 1.56E-11 | 350474 | 0.011 | 39.062 |
| Platelet count | rs2142218 | 21 | 16431444 | C | T | 0.157 | -0.02 | 0.003 | 2.94E-10 | 350474 | 0.013 | 44.444 |
| Platelet count | rs4257458 | 21 | 36404290 | G | A | 0.39 | 0.023 | 0.002 | 6.69E-22 | 350474 | 0.038 | 132.249 |
| Platelet count | rs4818033 | 21 | 40747624 | C | T | 0.321 | -0.018 | 0.002 | 8.37E-13 | 350474 | 0.023 | 81.000 |
| Platelet count | rs2238784 | 22 | 19972559 | A | G | 0.286 | 0.023 | 0.003 | 1.59E-19 | 350474 | 0.017 | 58.777 |
| Platelet count | rs3970551 | 22 | 18906839 | G | A | 0.115 | 0.02 | 0.004 | 3.33E-08 | 350474 | 0.007 | 25.000 |
| Platelet count | rs8138438 | 22 | 51080757 | G | A | 0.432 | 0.014 | 0.002 | 1.54E-09 | 350474 | 0.014 | 49.000 |
| Platelet count | rs5759002 | 22 | 43263432 | G | A | 0.521 | -0.033 | 0.002 | 3.43E-45 | 350474 | 0.078 | 272.248 |
| Platelet count | rs12484795 | 22 | 44343626 | C | A | 0.173 | -0.028 | 0.003 | 7.14E-20 | 350474 | 0.025 | 87.111 |
| Platelet count | rs138844 | 22 | 50184484 | G | T | 0.216 | 0.016 | 0.003 | 6.10E-09 | 350474 | 0.008 | 28.444 |
| Platelet count | rs75107793 | 22 | 50628937 | A | G | 0.073 | 0.107 | 0.004 | 1.12E-127 | 350474 | 0.204 | 715.558 |
| Platelet count | rs2097598 | 22 | 19689697 | G | C | 0.377 | -0.024 | 0.002 | 3.71E-23 | 350474 | 0.041 | 143.999 |
| Platelet count | rs12158292 | 22 | 29633234 | C | T | 0.563 | -0.017 | 0.002 | 1.55E-12 | 350474 | 0.021 | 72.250 |
| Platelet count | rs5763928 | 22 | 30678530 | G | A | 0.242 | -0.019 | 0.003 | 6.81E-12 | 350474 | 0.011 | 40.111 |
| Platelet count | rs855791 | 22 | 37462936 | G | A | 0.561 | -0.028 | 0.002 | 2.96E-33 | 350474 | 0.056 | 195.999 |
| Platelet count | rs148762611 | 22 | 50769029 | G | A | 0.347 | -0.019 | 0.002 | 7.63E-15 | 350474 | 0.026 | 90.249 |
| Platelet crit | rs2236055 | 1 | 12042261 | G | A | 0.468 | -0.047 | 0.002 | 4.22E-93 | 350471 | 0.157 | 552.247 |
| Platelet crit | rs1434282 | 1 | 199010721 | T | C | 0.726 | 0.04 | 0.003 | 5.54E-56 | 350471 | 0.051 | 177.777 |
| Platelet crit | rs928391 | 1 | 156776717 | C | T | 0.268 | -0.021 | 0.003 | 9.07E-17 | 350471 | 0.014 | 49.000 |
| Platelet crit | rs4284303 | 1 | 204276172 | C | T | 0.279 | -0.02 | 0.003 | 1.10E-14 | 350471 | 0.013 | 44.444 |
| Platelet crit | rs6696074 | 1 | 225962690 | T | C | 0.566 | 0.016 | 0.002 | 3.12E-12 | 350471 | 0.018 | 64.000 |
| Platelet crit | rs12142474 | 1 | 68056314 | G | A | 0.085 | -0.023 | 0.004 | 3.15E-08 | 350471 | 0.009 | 33.062 |
| Platelet crit | rs2274319 | 1 | 156450873 | C | T | 0.652 | -0.026 | 0.002 | 6.92E-27 | 350471 | 0.048 | 168.999 |
| Platelet crit | rs182050989 | 1 | 27262545 | T | C | 0.029 | 0.071 | 0.007 | 1.77E-25 | 350471 | 0.029 | 102.877 |
| Platelet crit | rs16829945 | 1 | 25040315 | T | C | 0.25 | -0.023 | 0.003 | 1.22E-18 | 350471 | 0.017 | 58.777 |
| Platelet crit | rs75139539 | 1 | 43840982 | T | C | 0.033 | 0.053 | 0.006 | 1.14E-16 | 350471 | 0.022 | 78.027 |
| Platelet crit | rs78261031 | 1 | 156112470 | A | G | 0.059 | -0.034 | 0.005 | 3.08E-12 | 350471 | 0.013 | 46.240 |
| Platelet crit | rs78634365 | 1 | 153667317 | C | A | 0.486 | 0.016 | 0.002 | 9.16E-12 | 350471 | 0.018 | 64.000 |
| Platelet crit | rs1926310 | 1 | 65188498 | C | G | 0.236 | -0.017 | 0.003 | 8.28E-10 | 350471 | 0.009 | 32.111 |
| Platelet crit | rs2089366 | 1 | 91930841 | T | C | 0.486 | 0.014 | 0.002 | 1.29E-09 | 350471 | 0.014 | 49.000 |
| Platelet crit | rs56043070 | 1 | 247719769 | A | G | 0.072 | -0.077 | 0.004 | 4.86E-69 | 350471 | 0.106 | 370.560 |
| Platelet crit | rs55794721 | 1 | 25561667 | A | G | 0.407 | -0.04 | 0.002 | 4.35E-68 | 350471 | 0.114 | 399.998 |
| Platelet crit | rs1569419 | 1 | 2996602 | C | T | 0.767 | 0.039 | 0.003 | 3.34E-47 | 350471 | 0.048 | 168.999 |
| Platelet crit | rs2047094 | 1 | 8917815 | A | C | 0.503 | 0.028 | 0.002 | 1.05E-35 | 350471 | 0.056 | 195.999 |
| Platelet crit | rs7525849 | 1 | 45859343 | G | A | 0.253 | -0.028 | 0.003 | 1.89E-26 | 350471 | 0.025 | 87.111 |
| Platelet crit | rs1694367 | 1 | 150329469 | A | T | 0.393 | 0.021 | 0.002 | 2.54E-20 | 350471 | 0.031 | 110.249 |
| Platelet crit | rs34754277 | 1 | 118147892 | C | T | 0.168 | 0.023 | 0.003 | 3.40E-14 | 350471 | 0.017 | 58.777 |
| Platelet crit | rs7413585 | 1 | 94606871 | C | T | 0.517 | -0.017 | 0.002 | 4.92E-14 | 350471 | 0.021 | 72.250 |
| Platelet crit | rs28570583 | 1 | 41422196 | G | A | 0.204 | 0.02 | 0.003 | 3.91E-12 | 350471 | 0.013 | 44.444 |
| Platelet crit | rs1539019 | 1 | 247600301 | C | A | 0.62 | 0.016 | 0.002 | 5.15E-12 | 350471 | 0.018 | 64.000 |
| Platelet crit | rs425709 | 1 | 112653291 | T | C | 0.756 | 0.017 | 0.003 | 3.27E-10 | 350471 | 0.009 | 32.111 |
| Platelet crit | rs10912870 | 1 | 174988016 | T | C | 0.336 | -0.015 | 0.002 | 4.19E-10 | 350471 | 0.016 | 56.250 |
| Platelet crit | rs3134615 | 1 | 40362066 | A | C | 0.254 | 0.015 | 0.003 | 2.65E-08 | 350471 | 0.007 | 25.000 |
| Platelet crit | rs4655557 | 1 | 66080787 | C | T | 0.366 | -0.018 | 0.002 | 7.94E-15 | 350471 | 0.023 | 81.000 |
| Platelet crit | rs16865296 | 1 | 185069843 | A | T | 0.058 | -0.035 | 0.005 | 1.31E-12 | 350471 | 0.014 | 49.000 |
| Platelet crit | rs2029858 | 1 | 62107806 | C | T | 0.781 | -0.019 | 0.003 | 1.18E-11 | 350471 | 0.011 | 40.111 |
| Platelet crit | rs140436199 | 1 | 88102437 | A | G | 0.058 | -0.05 | 0.005 | 2.24E-24 | 350471 | 0.029 | 99.999 |
| Platelet crit | rs10127775 | 1 | 230295789 | T | A | 0.607 | 0.019 | 0.002 | 8.88E-17 | 350471 | 0.026 | 90.249 |
| Platelet crit | rs2503706 | 1 | 2075251 | G | A | 0.678 | -0.02 | 0.002 | 3.02E-16 | 350471 | 0.029 | 99.999 |
| Platelet crit | rs147908554 | 1 | 202324006 | C | T | 0.019 | -0.067 | 0.008 | 5.05E-16 | 350471 | 0.020 | 70.140 |
| Platelet crit | rs3795503 | 1 | 180905694 | T | C | 0.316 | -0.019 | 0.002 | 3.31E-14 | 350471 | 0.026 | 90.249 |
| Platelet crit | rs28706215 | 1 | 236702973 | T | A | 0.695 | -0.016 | 0.002 | 1.97E-10 | 350471 | 0.018 | 64.000 |
| Platelet crit | rs11586371 | 1 | 178058063 | C | T | 0.103 | 0.024 | 0.004 | 2.79E-10 | 350471 | 0.010 | 36.000 |
| Platelet crit | rs10048745 | 2 | 68962137 | A | G | 0.256 | 0.027 | 0.003 | 4.54E-24 | 350471 | 0.023 | 81.000 |
| Platelet crit | rs6706095 | 2 | 46075677 | G | T | 0.76 | 0.021 | 0.003 | 1.02E-14 | 350471 | 0.014 | 49.000 |
| Platelet crit | rs4251961 | 2 | 113874467 | C | T | 0.376 | 0.015 | 0.002 | 3.12E-10 | 350471 | 0.016 | 56.250 |
| Platelet crit | rs79447483 | 2 | 112312475 | A | G | 0.132 | -0.02 | 0.003 | 3.40E-09 | 350471 | 0.013 | 44.444 |
| Platelet crit | rs12471768 | 2 | 64928603 | C | T | 0.705 | 0.014 | 0.002 | 8.19E-09 | 350471 | 0.014 | 49.000 |
| Platelet crit | rs56263467 | 2 | 234274667 | T | G | 0.122 | -0.035 | 0.003 | 6.12E-24 | 350471 | 0.039 | 136.110 |
| Platelet crit | rs12987212 | 2 | 100761156 | T | C | 0.358 | 0.021 | 0.002 | 7.10E-19 | 350471 | 0.031 | 110.249 |
| Platelet crit | rs78909033 | 2 | 241510903 | A | G | 0.135 | -0.024 | 0.003 | 4.92E-13 | 350471 | 0.018 | 64.000 |
| Platelet crit | rs75770358 | 2 | 191248847 | T | C | 0.326 | 0.017 | 0.002 | 2.08E-12 | 350471 | 0.021 | 72.250 |
| Platelet crit | rs6708784 | 2 | 111927379 | G | A | 0.498 | -0.014 | 0.002 | 6.77E-10 | 350471 | 0.014 | 49.000 |
| Platelet crit | rs72932729 | 2 | 203650267 | T | C | 0.475 | 0.013 | 0.002 | 3.30E-08 | 350471 | 0.012 | 42.250 |
| Platelet crit | rs11690316 | 2 | 219017462 | G | A | 0.519 | 0.016 | 0.002 | 5.69E-13 | 350471 | 0.018 | 64.000 |
| Platelet crit | rs147348486 | 2 | 85766300 | A | G | 0.016 | 0.057 | 0.009 | 5.49E-10 | 350471 | 0.011 | 40.111 |
| Platelet crit | rs12693829 | 2 | 198885425 | T | G | 0.679 | 0.015 | 0.002 | 2.44E-09 | 350471 | 0.016 | 56.250 |
| Platelet crit | rs12052715 | 2 | 160677375 | G | C | 0.727 | -0.043 | 0.003 | 1.30E-64 | 350471 | 0.059 | 205.443 |
| Platelet crit | rs1260326 | 2 | 27730940 | C | T | 0.607 | -0.038 | 0.002 | 4.13E-60 | 350471 | 0.103 | 360.998 |
| Platelet crit | rs3769631 | 2 | 31462373 | A | C | 0.716 | 0.036 | 0.003 | 8.46E-47 | 350471 | 0.041 | 143.999 |
| Platelet crit | rs11676298 | 2 | 227291731 | G | C | 0.193 | 0.038 | 0.003 | 5.13E-39 | 350471 | 0.046 | 160.444 |
| Platelet crit | rs10167625 | 2 | 170579634 | T | C | 0.619 | 0.016 | 0.002 | 2.09E-11 | 350471 | 0.018 | 64.000 |
| Platelet crit | rs9653568 | 2 | 85466885 | G | A | 0.448 | 0.014 | 0.002 | 3.23E-09 | 350471 | 0.014 | 49.000 |
| Platelet crit | rs149290349 | 2 | 43451957 | A | G | 0.075 | -0.066 | 0.004 | 1.97E-52 | 350471 | 0.078 | 272.248 |
| Platelet crit | rs62160676 | 2 | 112167931 | C | T | 0.297 | 0.033 | 0.002 | 8.06E-41 | 350471 | 0.078 | 272.248 |
| Platelet crit | rs72781680 | 2 | 24238928 | T | C | 0.135 | -0.036 | 0.003 | 1.46E-26 | 350471 | 0.041 | 143.999 |
| Platelet crit | rs4669879 | 2 | 12942653 | C | A | 0.451 | 0.02 | 0.002 | 9.33E-18 | 350471 | 0.029 | 99.999 |
| Platelet crit | rs76840964 | 2 | 160754947 | G | A | 0.125 | -0.028 | 0.003 | 1.53E-15 | 350471 | 0.025 | 87.111 |
| Platelet crit | rs12621712 | 2 | 74716140 | G | A | 0.142 | 0.022 | 0.003 | 9.35E-12 | 350471 | 0.015 | 53.777 |
| Platelet crit | rs1250247 | 2 | 216299629 | G | C | 0.727 | -0.015 | 0.003 | 6.67E-09 | 350471 | 0.007 | 25.000 |
| Platelet crit | rs6796069 | 3 | 58332508 | T | C | 0.326 | -0.024 | 0.002 | 6.22E-24 | 350471 | 0.041 | 143.999 |
| Platelet crit | rs9809116 | 3 | 72397279 | G | A | 0.407 | 0.018 | 0.002 | 8.80E-15 | 350471 | 0.023 | 81.000 |
| Platelet crit | rs12497690 | 3 | 27795397 | C | A | 0.371 | 0.017 | 0.002 | 5.84E-13 | 350471 | 0.021 | 72.250 |
| Platelet crit | rs4686388 | 3 | 185484257 | A | G | 0.632 | -0.014 | 0.002 | 1.99E-09 | 350471 | 0.014 | 49.000 |
| Platelet crit | rs165177 | 3 | 8603243 | C | T | 0.746 | 0.016 | 0.003 | 2.54E-09 | 350471 | 0.008 | 28.444 |
| Platelet crit | rs78565404 | 3 | 184090242 | T | C | 0.053 | 0.134 | 0.005 | 1.80E-148 | 350471 | 0.205 | 718.236 |
| Platelet crit | rs11715288 | 3 | 183748637 | C | T | 0.546 | 0.023 | 0.002 | 9.08E-23 | 350471 | 0.038 | 132.249 |
| Platelet crit | rs9310842 | 3 | 27369765 | T | A | 0.728 | 0.019 | 0.003 | 9.58E-14 | 350471 | 0.011 | 40.111 |
| Platelet crit | rs116223294 | 3 | 184112652 | A | C | 0.014 | 0.067 | 0.01 | 5.57E-12 | 350471 | 0.013 | 44.890 |
| Platelet crit | rs1394094 | 3 | 136195788 | T | A | 0.641 | 0.016 | 0.002 | 7.01E-12 | 350471 | 0.018 | 64.000 |
| Platelet crit | rs137975544 | 3 | 149083238 | T | G | 0.366 | 0.016 | 0.002 | 8.62E-11 | 350471 | 0.018 | 64.000 |
| Platelet crit | rs150858838 | 3 | 48956257 | G | A | 0.034 | 0.038 | 0.006 | 2.39E-09 | 350471 | 0.011 | 40.111 |
| Platelet crit | rs12495290 | 3 | 156819988 | A | G | 0.147 | -0.019 | 0.003 | 7.02E-09 | 350471 | 0.011 | 40.111 |
| Platelet crit | rs4857906 | 3 | 128275459 | T | C | 0.195 | -0.016 | 0.003 | 4.30E-08 | 350471 | 0.008 | 28.444 |
| Platelet crit | rs12631447 | 3 | 168858226 | C | T | 0.625 | -0.034 | 0.002 | 1.36E-46 | 350471 | 0.082 | 288.998 |
| Platelet crit | rs1354034 | 3 | 56849749 | C | T | 0.601 | 0.029 | 0.002 | 3.78E-35 | 350471 | 0.060 | 210.249 |
| Platelet crit | rs16858720 | 3 | 184066615 | G | A | 0.125 | 0.041 | 0.003 | 1.47E-31 | 350471 | 0.053 | 186.777 |
| Platelet crit | rs115169601 | 3 | 48597018 | A | G | 0.032 | -0.053 | 0.007 | 2.10E-15 | 350471 | 0.016 | 57.326 |
| Platelet crit | rs55931951 | 3 | 123060758 | C | G | 0.221 | -0.021 | 0.003 | 9.67E-15 | 350471 | 0.014 | 49.000 |
| Platelet crit | rs55921103 | 3 | 69810294 | T | G | 0.651 | -0.015 | 0.002 | 1.38E-09 | 350471 | 0.016 | 56.250 |
| Platelet crit | rs7641761 | 3 | 178740422 | A | T | 0.697 | -0.015 | 0.002 | 3.85E-09 | 350471 | 0.016 | 56.250 |
| Platelet crit | rs116712492 | 3 | 172252149 | C | T | 0.047 | 0.031 | 0.005 | 1.10E-08 | 350471 | 0.011 | 38.440 |
| Platelet crit | rs12491937 | 3 | 12268244 | G | A | 0.418 | -0.037 | 0.002 | 4.09E-57 | 350471 | 0.098 | 342.248 |
| Platelet crit | rs873093 | 3 | 107501282 | C | T | 0.386 | 0.018 | 0.002 | 8.22E-15 | 350471 | 0.023 | 81.000 |
| Platelet crit | rs10510412 | 3 | 12346962 | A | G | 0.241 | 0.02 | 0.003 | 3.27E-14 | 350471 | 0.013 | 44.444 |
| Platelet crit | rs3792379 | 3 | 122845165 | C | A | 0.519 | -0.014 | 0.002 | 8.49E-10 | 350471 | 0.014 | 49.000 |
| Platelet crit | rs2089979 | 3 | 196501413 | G | A | 0.418 | -0.025 | 0.002 | 4.83E-28 | 350471 | 0.045 | 156.249 |
| Platelet crit | rs10934681 | 3 | 124450321 | C | T | 0.261 | -0.025 | 0.003 | 1.89E-21 | 350471 | 0.020 | 69.444 |
| Platelet crit | rs4553954 | 3 | 101233149 | G | A | 0.404 | -0.015 | 0.002 | 2.57E-10 | 350471 | 0.016 | 56.250 |
| Platelet crit | rs2298993 | 4 | 110895113 | A | G | 0.402 | -0.028 | 0.002 | 1.52E-33 | 350471 | 0.056 | 195.999 |
| Platelet crit | rs2699425 | 4 | 3478051 | C | T | 0.373 | 0.017 | 0.002 | 2.03E-13 | 350471 | 0.021 | 72.250 |
| Platelet crit | rs4588 | 4 | 72618323 | T | G | 0.292 | -0.014 | 0.002 | 6.50E-09 | 350471 | 0.014 | 49.000 |
| Platelet crit | rs7665170 | 4 | 185788095 | T | C | 0.455 | -0.016 | 0.002 | 2.19E-12 | 350471 | 0.018 | 64.000 |
| Platelet crit | rs5023357 | 4 | 124770793 | G | T | 0.109 | 0.024 | 0.004 | 2.90E-11 | 350471 | 0.010 | 36.000 |
| Platelet crit | rs7665147 | 4 | 57767327 | A | T | 0.186 | -0.018 | 0.003 | 3.36E-10 | 350471 | 0.010 | 36.000 |
| Platelet crit | rs2868342 | 4 | 83238255 | A | C | 0.231 | -0.016 | 0.003 | 2.87E-09 | 350471 | 0.008 | 28.444 |
| Platelet crit | rs72952310 | 4 | 106206542 | T | G | 0.032 | -0.062 | 0.006 | 1.58E-21 | 350471 | 0.030 | 106.777 |
| Platelet crit | rs112969588 | 4 | 157692093 | C | T | 0.092 | -0.031 | 0.004 | 1.69E-15 | 350471 | 0.017 | 60.062 |
| Platelet crit | rs218265 | 4 | 55408999 | C | T | 0.155 | 0.023 | 0.003 | 1.08E-12 | 350471 | 0.017 | 58.777 |
| Platelet crit | rs7676961 | 4 | 39684217 | C | T | 0.507 | -0.015 | 0.002 | 1.44E-11 | 350471 | 0.016 | 56.250 |
| Platelet crit | rs7671752 | 4 | 17661398 | T | C | 0.587 | -0.015 | 0.002 | 5.69E-11 | 350471 | 0.016 | 56.250 |
| Platelet crit | rs6815294 | 4 | 7042349 | A | G | 0.573 | 0.026 | 0.002 | 7.12E-29 | 350471 | 0.048 | 168.999 |
| Platelet crit | rs71633359 | 4 | 88183820 | C | T | 0.32 | 0.021 | 0.002 | 1.91E-16 | 350471 | 0.031 | 110.249 |
| Platelet crit | rs7694483 | 4 | 120405929 | A | T | 0.516 | -0.017 | 0.002 | 1.55E-13 | 350471 | 0.021 | 72.250 |
| Platelet crit | rs4565031 | 4 | 145044759 | A | G | 0.55 | -0.016 | 0.002 | 6.93E-13 | 350471 | 0.018 | 64.000 |
| Platelet crit | rs28666858 | 4 | 152416382 | C | T | 0.558 | 0.02 | 0.002 | 3.40E-19 | 350471 | 0.029 | 99.999 |
| Platelet crit | rs144317085 | 4 | 105806108 | T | A | 0.034 | 0.051 | 0.006 | 6.95E-16 | 350471 | 0.021 | 72.250 |
| Platelet crit | rs13118519 | 4 | 141851355 | T | C | 0.501 | -0.018 | 0.002 | 1.69E-14 | 350471 | 0.023 | 81.000 |
| Platelet crit | rs114694170 | 5 | 88180196 | C | T | 0.059 | 0.081 | 0.005 | 3.92E-62 | 350471 | 0.075 | 262.439 |
| Platelet crit | rs10793824 | 5 | 137824833 | T | C | 0.432 | 0.014 | 0.002 | 3.40E-09 | 350471 | 0.014 | 49.000 |
| Platelet crit | rs62371571 | 5 | 111110597 | G | T | 0.106 | 0.022 | 0.004 | 3.81E-09 | 350471 | 0.009 | 30.250 |
| Platelet crit | rs34325695 | 5 | 88044252 | A | G | 0.032 | 0.049 | 0.006 | 3.14E-14 | 350471 | 0.019 | 66.694 |
| Platelet crit | rs61215818 | 5 | 111059676 | A | G | 0.077 | -0.064 | 0.004 | 6.30E-50 | 350471 | 0.073 | 255.999 |
| Platelet crit | rs4704727 | 5 | 156380067 | G | T | 0.66 | -0.017 | 0.002 | 3.18E-13 | 350471 | 0.021 | 72.250 |
| Platelet crit | rs10941418 | 5 | 39080694 | G | A | 0.442 | -0.013 | 0.002 | 1.82E-08 | 350471 | 0.012 | 42.250 |
| Platelet crit | rs7705526 | 5 | 1285974 | A | C | 0.325 | 0.051 | 0.002 | 1.17E-97 | 350471 | 0.185 | 650.246 |
| Platelet crit | rs28727880 | 5 | 77683507 | A | G | 0.496 | 0.029 | 0.002 | 8.19E-38 | 350471 | 0.060 | 210.249 |
| Platelet crit | rs72648830 | 5 | 177608542 | T | C | 0.11 | -0.041 | 0.004 | 6.54E-30 | 350471 | 0.030 | 105.062 |
| Platelet crit | rs2438150 | 5 | 122199196 | T | C | 0.555 | 0.025 | 0.002 | 3.97E-27 | 350471 | 0.045 | 156.249 |
| Platelet crit | rs13189073 | 5 | 1307648 | A | G | 0.115 | -0.024 | 0.004 | 1.21E-11 | 350471 | 0.010 | 36.000 |
| Platelet crit | rs35123781 | 5 | 139065180 | G | A | 0.333 | -0.015 | 0.002 | 1.15E-09 | 350471 | 0.016 | 56.250 |
| Platelet crit | rs7703616 | 5 | 1115115 | T | C | 0.593 | -0.013 | 0.002 | 1.15E-08 | 350471 | 0.012 | 42.250 |
| Platelet crit | rs210213 | 6 | 33572159 | A | C | 0.67 | -0.037 | 0.002 | 3.35E-51 | 350471 | 0.098 | 342.248 |
| Platelet crit | rs3173420 | 6 | 29911114 | A | G | 0.522 | 0.029 | 0.002 | 4.12E-36 | 350471 | 0.060 | 210.249 |
| Platelet crit | rs210798 | 6 | 135514558 | G | T | 0.521 | -0.026 | 0.002 | 3.54E-29 | 350471 | 0.048 | 168.999 |
| Platelet crit | rs9460100 | 6 | 170583022 | C | T | 0.551 | -0.018 | 0.002 | 1.15E-14 | 350471 | 0.023 | 81.000 |
| Platelet crit | rs511515 | 6 | 33541507 | G | A | 0.699 | 0.103 | 0.002 | 1.00E-200 | 350471 | 0.751 | 2652.235 |
| Platelet crit | rs116234817 | 6 | 31321364 | A | G | 0.052 | 0.082 | 0.005 | 1.30E-57 | 350471 | 0.077 | 268.958 |
| Platelet crit | rs4715332 | 6 | 52669185 | A | C | 0.572 | -0.024 | 0.002 | 1.88E-25 | 350471 | 0.041 | 143.999 |
| Platelet crit | rs9320282 | 6 | 109619377 | G | A | 0.523 | 0.019 | 0.002 | 2.99E-17 | 350471 | 0.026 | 90.249 |
| Platelet crit | rs1187115 | 6 | 34172055 | A | C | 0.83 | 0.024 | 0.003 | 2.71E-15 | 350471 | 0.018 | 64.000 |
| Platelet crit | rs9389269 | 6 | 135427159 | C | T | 0.273 | 0.121 | 0.003 | 1.00E-200 | 350471 | 0.462 | 1626.768 |
| Platelet crit | rs381500 | 6 | 164478388 | A | C | 0.452 | 0.035 | 0.002 | 1.81E-53 | 350471 | 0.087 | 306.248 |
| Platelet crit | rs71531541 | 6 | 7066481 | T | C | 0.728 | 0.021 | 0.003 | 6.27E-16 | 350471 | 0.014 | 49.000 |
| Platelet crit | rs1611236 | 6 | 29748690 | A | G | 0.322 | -0.019 | 0.002 | 1.78E-15 | 350471 | 0.026 | 90.249 |
| Platelet crit | rs484552 | 6 | 122749893 | T | A | 0.506 | 0.016 | 0.002 | 1.30E-12 | 350471 | 0.018 | 64.000 |
| Platelet crit | rs9457338 | 6 | 158730742 | A | G | 0.905 | 0.024 | 0.004 | 1.51E-09 | 350471 | 0.010 | 36.000 |
| Platelet crit | rs117199940 | 6 | 158929980 | T | C | 0.058 | 0.027 | 0.005 | 2.39E-08 | 350471 | 0.008 | 29.160 |
| Platelet crit | rs1040804 | 6 | 135042768 | A | T | 0.209 | 0.042 | 0.003 | 5.84E-51 | 350471 | 0.056 | 195.999 |
| Platelet crit | rs12528656 | 6 | 41916072 | G | T | 0.285 | 0.017 | 0.003 | 6.57E-11 | 350471 | 0.009 | 32.111 |
| Platelet crit | rs6926250 | 6 | 131302473 | C | T | 0.414 | 0.015 | 0.002 | 1.05E-10 | 350471 | 0.016 | 56.250 |
| Platelet crit | rs62408225 | 6 | 90956409 | G | A | 0.352 | -0.015 | 0.002 | 7.47E-10 | 350471 | 0.016 | 56.250 |
| Platelet crit | rs112370520 | 6 | 26036092 | T | C | 0.121 | -0.021 | 0.003 | 2.07E-09 | 350471 | 0.014 | 49.000 |
| Platelet crit | rs12155039 | 7 | 44873125 | A | C | 0.435 | -0.047 | 0.002 | 6.15E-92 | 350471 | 0.157 | 552.247 |
| Platelet crit | rs35150201 | 7 | 135346262 | G | T | 0.484 | -0.027 | 0.002 | 5.49E-33 | 350471 | 0.052 | 182.249 |
| Platelet crit | rs17138703 | 7 | 18201223 | G | A | 0.213 | -0.031 | 0.003 | 1.74E-28 | 350471 | 0.030 | 106.777 |
| Platelet crit | rs2700936 | 7 | 36084910 | G | T | 0.459 | 0.016 | 0.002 | 5.78E-12 | 350471 | 0.018 | 64.000 |
| Platelet crit | rs11764390 | 7 | 80216205 | A | G | 0.438 | 0.016 | 0.002 | 1.15E-11 | 350471 | 0.018 | 64.000 |
| Platelet crit | rs877279 | 7 | 158430907 | T | C | 0.62 | 0.013 | 0.002 | 8.23E-09 | 350471 | 0.012 | 42.250 |
| Platelet crit | rs78026924 | 7 | 40152917 | A | T | 0.064 | 0.025 | 0.005 | 4.56E-08 | 350471 | 0.007 | 25.000 |
| Platelet crit | rs77738210 | 7 | 123428916 | T | G | 0.08 | 0.058 | 0.004 | 9.52E-44 | 350471 | 0.060 | 210.249 |
| Platelet crit | rs75612655 | 7 | 101594247 | T | C | 0.009 | 0.128 | 0.012 | 3.17E-25 | 350471 | 0.032 | 113.777 |
| Platelet crit | rs10240931 | 7 | 149419721 | C | G | 0.114 | -0.026 | 0.004 | 4.58E-13 | 350471 | 0.012 | 42.250 |
| Platelet crit | rs10954300 | 7 | 130761235 | A | G | 0.737 | -0.029 | 0.003 | 5.70E-30 | 350471 | 0.027 | 93.444 |
| Platelet crit | rs4548095 | 7 | 100226665 | T | C | 0.633 | 0.022 | 0.002 | 7.60E-21 | 350471 | 0.035 | 120.999 |
| Platelet crit | rs56403542 | 7 | 116528563 | G | A | 0.438 | 0.016 | 0.002 | 5.71E-12 | 350471 | 0.018 | 64.000 |
| Platelet crit | rs35355140 | 7 | 27204732 | A | C | 0.068 | -0.03 | 0.005 | 2.21E-11 | 350471 | 0.010 | 36.000 |
| Platelet crit | rs62491369 | 7 | 139773656 | T | G | 0.14 | -0.02 | 0.003 | 7.80E-10 | 350471 | 0.013 | 44.444 |
| Platelet crit | rs3757388 | 7 | 128576023 | A | G | 0.631 | -0.014 | 0.002 | 3.27E-09 | 350471 | 0.014 | 49.000 |
| Platelet crit | rs3213661 | 7 | 14026357 | G | A | 0.713 | -0.014 | 0.003 | 1.29E-08 | 350471 | 0.006 | 21.778 |
| Platelet crit | rs11982272 | 7 | 1970766 | C | T | 0.202 | -0.018 | 0.003 | 6.58E-10 | 350471 | 0.010 | 36.000 |
| Platelet crit | rs60750824 | 7 | 99927436 | G | A | 0.185 | 0.035 | 0.003 | 6.58E-31 | 350471 | 0.039 | 136.110 |
| Platelet crit | rs6592965 | 7 | 50427982 | A | G | 0.454 | 0.02 | 0.002 | 1.38E-17 | 350471 | 0.029 | 99.999 |
| Platelet crit | rs4947490 | 7 | 55160538 | G | A | 0.679 | 0.018 | 0.002 | 5.99E-14 | 350471 | 0.023 | 81.000 |
| Platelet crit | rs7784559 | 7 | 1002973 | G | A | 0.644 | 0.013 | 0.002 | 2.44E-08 | 350471 | 0.012 | 42.250 |
| Platelet crit | rs7833924 | 8 | 144996029 | G | A | 0.43 | 0.029 | 0.002 | 9.49E-38 | 350471 | 0.060 | 210.249 |
| Platelet crit | rs11786352 | 8 | 22437141 | A | G | 0.22 | 0.022 | 0.003 | 2.29E-15 | 350471 | 0.015 | 53.777 |
| Platelet crit | rs1823964 | 8 | 40038193 | G | A | 0.565 | 0.017 | 0.002 | 2.87E-14 | 350471 | 0.021 | 72.250 |
| Platelet crit | rs7814625 | 8 | 130541447 | A | G | 0.363 | -0.017 | 0.002 | 2.16E-12 | 350471 | 0.021 | 72.250 |
| Platelet crit | rs2915607 | 8 | 30279916 | C | T | 0.742 | 0.026 | 0.003 | 2.74E-24 | 350471 | 0.021 | 75.111 |
| Platelet crit | rs7010394 | 8 | 126343520 | C | T | 0.543 | 0.015 | 0.002 | 2.47E-11 | 350471 | 0.016 | 56.250 |
| Platelet crit | rs72665715 | 8 | 71348177 | A | G | 0.135 | 0.021 | 0.003 | 4.53E-10 | 350471 | 0.014 | 49.000 |
| Platelet crit | rs140753685 | 8 | 55407312 | C | T | 0.212 | -0.017 | 0.003 | 2.49E-09 | 350471 | 0.009 | 32.111 |
| Platelet crit | rs6993770 | 8 | 106581528 | T | A | 0.286 | -0.05 | 0.003 | 1.67E-88 | 350471 | 0.079 | 277.776 |
| Platelet crit | rs7832014 | 8 | 128846435 | A | G | 0.581 | -0.018 | 0.002 | 9.74E-15 | 350471 | 0.023 | 81.000 |
| Platelet crit | rs77338166 | 8 | 105469502 | G | A | 0.066 | -0.028 | 0.005 | 9.42E-10 | 350471 | 0.009 | 31.360 |
| Platelet crit | rs10086797 | 8 | 120993823 | A | G | 0.258 | -0.018 | 0.003 | 4.01E-12 | 350471 | 0.010 | 36.000 |
| Platelet crit | rs7000170 | 8 | 142322896 | A | G | 0.29 | -0.021 | 0.003 | 8.04E-17 | 350471 | 0.014 | 49.000 |
| Platelet crit | rs11995704 | 8 | 66926428 | G | A | 0.316 | -0.02 | 0.002 | 6.07E-16 | 350471 | 0.029 | 99.999 |
| Platelet crit | rs35469695 | 8 | 23406169 | G | C | 0.171 | -0.02 | 0.003 | 1.29E-11 | 350471 | 0.013 | 44.444 |
| Platelet crit | rs8176746 | 9 | 136131322 | T | G | 0.061 | -0.047 | 0.005 | 4.44E-23 | 350471 | 0.025 | 88.359 |
| Platelet crit | rs2140483 | 9 | 110532522 | A | C | 0.664 | -0.017 | 0.002 | 7.28E-13 | 350471 | 0.021 | 72.250 |
| Platelet crit | rs10821066 | 9 | 95840479 | G | A | 0.16 | 0.019 | 0.003 | 7.71E-10 | 350471 | 0.011 | 40.111 |
| Platelet crit | rs385893 | 9 | 4763176 | C | T | 0.526 | 0.106 | 0.002 | 1.00E-200 | 350471 | 0.795 | 2808.984 |
| Platelet crit | rs7036656 | 9 | 21990457 | T | C | 0.722 | 0.048 | 0.003 | 3.64E-79 | 350471 | 0.073 | 255.999 |
| Platelet crit | rs61750929 | 9 | 91495135 | T | C | 0.055 | -0.084 | 0.005 | 9.33E-64 | 350471 | 0.080 | 282.238 |
| Platelet crit | rs12376511 | 9 | 22142756 | C | T | 0.16 | -0.052 | 0.003 | 9.57E-64 | 350471 | 0.086 | 300.443 |
| Platelet crit | rs7033052 | 9 | 5042981 | C | G | 0.531 | -0.034 | 0.002 | 3.51E-50 | 350471 | 0.082 | 288.998 |
| Platelet crit | rs11794772 | 9 | 99118445 | A | G | 0.257 | 0.032 | 0.003 | 1.50E-35 | 350471 | 0.032 | 113.777 |
| Platelet crit | rs9299006 | 9 | 4889324 | A | G | 0.008 | -0.095 | 0.013 | 7.69E-14 | 350471 | 0.015 | 53.402 |
| Platelet crit | rs2416759 | 9 | 123358262 | A | G | 0.697 | 0.015 | 0.002 | 1.59E-09 | 350471 | 0.016 | 56.250 |
| Platelet crit | rs296852 | 9 | 4787167 | A | G | 0.635 | -0.043 | 0.002 | 1.85E-71 | 350471 | 0.132 | 462.247 |
| Platelet crit | rs28578007 | 9 | 139838690 | C | T | 0.521 | -0.016 | 0.002 | 1.49E-12 | 350471 | 0.018 | 64.000 |
| Platelet crit | rs681470 | 9 | 135865359 | G | A | 0.128 | 0.019 | 0.003 | 1.51E-08 | 350471 | 0.011 | 40.111 |
| Platelet crit | rs7860776 | 9 | 4836566 | A | G | 0.291 | -0.035 | 0.003 | 1.42E-41 | 350471 | 0.039 | 136.110 |
| Platelet crit | rs10974836 | 9 | 4888724 | C | T | 0.908 | 0.023 | 0.004 | 4.15E-09 | 350471 | 0.009 | 33.062 |
| Platelet crit | rs10973700 | 9 | 38196117 | C | G | 0.491 | -0.024 | 0.002 | 4.55E-25 | 350471 | 0.041 | 143.999 |
| Platelet crit | rs10818963 | 9 | 127189915 | A | T | 0.653 | -0.023 | 0.002 | 2.37E-21 | 350471 | 0.038 | 132.249 |
| Platelet crit | rs10991780 | 9 | 93906723 | C | T | 0.928 | 0.03 | 0.004 | 4.85E-12 | 350471 | 0.016 | 56.250 |
| Platelet crit | rs760402 | 9 | 35686376 | A | G | 0.688 | -0.015 | 0.002 | 1.34E-09 | 350471 | 0.016 | 56.250 |
| Platelet crit | rs725529 | 10 | 63814070 | T | A | 0.369 | 0.015 | 0.002 | 7.27E-11 | 350471 | 0.016 | 56.250 |
| Platelet crit | rs11259151 | 10 | 14562793 | T | C | 0.491 | -0.014 | 0.002 | 9.77E-10 | 350471 | 0.014 | 49.000 |
| Platelet crit | rs12412214 | 10 | 101276256 | A | G | 0.284 | -0.026 | 0.003 | 1.47E-25 | 350471 | 0.021 | 75.111 |
| Platelet crit | rs224082 | 10 | 64566258 | T | C | 0.376 | -0.02 | 0.002 | 1.74E-17 | 350471 | 0.029 | 99.999 |
| Platelet crit | rs7920539 | 10 | 43880833 | T | C | 0.441 | -0.014 | 0.002 | 2.30E-09 | 350471 | 0.014 | 49.000 |
| Platelet crit | rs11818463 | 10 | 25223367 | C | G | 0.37 | -0.026 | 0.002 | 3.27E-28 | 350471 | 0.048 | 168.999 |
| Platelet crit | rs116052829 | 10 | 81164146 | T | C | 0.103 | 0.033 | 0.004 | 1.42E-18 | 350471 | 0.019 | 68.062 |
| Platelet crit | rs7080386 | 10 | 65048306 | A | C | 0.414 | 0.016 | 0.002 | 1.24E-12 | 350471 | 0.018 | 64.000 |
| Platelet crit | rs1537132 | 10 | 30249153 | C | T | 0.49 | 0.019 | 0.002 | 9.72E-18 | 350471 | 0.026 | 90.249 |
| Platelet crit | rs2077218 | 10 | 96071561 | A | G | 0.76 | 0.022 | 0.003 | 6.99E-17 | 350471 | 0.015 | 53.777 |
| Platelet crit | rs17464824 | 10 | 17210822 | C | A | 0.532 | 0.016 | 0.002 | 5.17E-12 | 350471 | 0.018 | 64.000 |
| Platelet crit | rs41297163 | 10 | 91371329 | G | A | 0.109 | 0.022 | 0.004 | 3.42E-09 | 350471 | 0.009 | 30.250 |
| Platelet crit | rs28505677 | 10 | 50259007 | G | C | 0.237 | -0.037 | 0.003 | 4.99E-42 | 350471 | 0.043 | 152.110 |
| Platelet crit | rs2152240 | 10 | 120822033 | G | T | 0.437 | 0.014 | 0.002 | 2.62E-10 | 350471 | 0.014 | 49.000 |
| Platelet crit | rs60335838 | 10 | 114074087 | T | A | 0.49 | -0.013 | 0.002 | 6.86E-09 | 350471 | 0.012 | 42.250 |
| Platelet crit | rs2155380 | 11 | 119080037 | G | A | 0.271 | 0.056 | 0.003 | 4.90E-108 | 350471 | 0.099 | 348.442 |
| Platelet crit | rs5130 | 11 | 116703146 | T | C | 0.868 | 0.026 | 0.003 | 3.95E-15 | 350471 | 0.021 | 75.111 |
| Platelet crit | rs72895301 | 11 | 32723257 | G | T | 0.04 | -0.044 | 0.006 | 6.22E-14 | 350471 | 0.015 | 53.777 |
| Platelet crit | rs55966801 | 11 | 200343 | C | T | 0.231 | 0.039 | 0.003 | 8.49E-47 | 350471 | 0.048 | 168.999 |
| Platelet crit | rs7103603 | 11 | 128297998 | A | G | 0.39 | -0.027 | 0.002 | 8.98E-31 | 350471 | 0.052 | 182.249 |
| Platelet crit | rs2283228 | 11 | 2849530 | C | A | 0.074 | 0.026 | 0.004 | 4.60E-09 | 350471 | 0.012 | 42.250 |
| Platelet crit | rs10832728 | 11 | 17070581 | G | A | 0.523 | -0.012 | 0.002 | 3.97E-08 | 350471 | 0.010 | 36.000 |
| Platelet crit | rs682845 | 11 | 65254201 | C | T | 0.335 | -0.021 | 0.002 | 7.10E-19 | 350471 | 0.031 | 110.249 |
| Platelet crit | rs62621409 | 11 | 36458997 | G | A | 0.043 | 0.034 | 0.006 | 6.78E-10 | 350471 | 0.009 | 32.111 |
| Platelet crit | rs7124215 | 11 | 64938066 | G | T | 0.184 | 0.018 | 0.003 | 1.00E-09 | 350471 | 0.010 | 36.000 |
| Platelet crit | rs7934719 | 11 | 108341864 | T | C | 0.409 | 0.03 | 0.002 | 9.64E-39 | 350471 | 0.064 | 224.999 |
| Platelet crit | rs4910442 | 11 | 9044337 | C | T | 0.446 | -0.02 | 0.002 | 7.30E-19 | 350471 | 0.029 | 99.999 |
| Platelet crit | rs3741156 | 11 | 72946140 | C | G | 0.24 | 0.02 | 0.003 | 2.53E-14 | 350471 | 0.013 | 44.444 |
| Platelet crit | rs174574 | 11 | 61600342 | C | A | 0.647 | -0.02 | 0.002 | 3.17E-17 | 350471 | 0.029 | 99.999 |
| Platelet crit | rs231353 | 11 | 2709019 | G | A | 0.348 | -0.02 | 0.002 | 1.70E-16 | 350471 | 0.029 | 99.999 |
| Platelet crit | rs72996119 | 11 | 100457812 | G | A | 0.097 | -0.03 | 0.004 | 9.09E-15 | 350471 | 0.016 | 56.250 |
| Platelet crit | rs10750385 | 11 | 127937766 | G | A | 0.672 | 0.016 | 0.002 | 9.73E-12 | 350471 | 0.018 | 64.000 |
| Platelet crit | rs4936066 | 11 | 128584765 | C | A | 0.19 | -0.018 | 0.003 | 4.01E-10 | 350471 | 0.010 | 36.000 |
| Platelet crit | rs11225007 | 11 | 101667294 | C | T | 0.084 | 0.023 | 0.004 | 1.29E-08 | 350471 | 0.009 | 33.062 |
| Platelet crit | rs4937124 | 11 | 126278563 | C | T | 0.473 | -0.013 | 0.002 | 2.34E-08 | 350471 | 0.012 | 42.250 |
| Platelet crit | rs4426188 | 12 | 80263235 | A | C | 0.941 | -0.03 | 0.005 | 2.88E-10 | 350471 | 0.010 | 36.000 |
| Platelet crit | rs1074958 | 12 | 62664272 | T | C | 0.099 | -0.024 | 0.004 | 6.53E-10 | 350471 | 0.010 | 36.000 |
| Platelet crit | rs138003409 | 12 | 110852606 | G | C | 0.024 | -0.045 | 0.008 | 1.85E-08 | 350471 | 0.009 | 31.640 |
| Platelet crit | rs79977579 | 12 | 54694560 | A | C | 0.1 | 0.061 | 0.004 | 4.65E-58 | 350471 | 0.066 | 232.561 |
| Platelet crit | rs4020660 | 12 | 6511657 | A | G | 0.465 | -0.025 | 0.002 | 6.78E-27 | 350471 | 0.045 | 156.249 |
| Platelet crit | rs4913405 | 12 | 68517877 | A | G | 0.752 | 0.019 | 0.003 | 3.41E-13 | 350471 | 0.011 | 40.111 |
| Platelet crit | rs11553699 | 12 | 122216910 | G | A | 0.135 | 0.112 | 0.003 | 1.00E-200 | 350471 | 0.396 | 1393.770 |
| Platelet crit | rs3742028 | 12 | 109491468 | A | G | 0.582 | -0.046 | 0.002 | 3.36E-87 | 350471 | 0.151 | 528.997 |
| Platelet crit | rs7953929 | 12 | 123758235 | G | C | 0.201 | -0.032 | 0.003 | 1.55E-29 | 350471 | 0.032 | 113.777 |
| Platelet crit | rs2255531 | 12 | 121414915 | A | G | 0.348 | -0.026 | 0.002 | 7.45E-27 | 350471 | 0.048 | 168.999 |
| Platelet crit | rs10734834 | 12 | 10640984 | T | C | 0.877 | -0.024 | 0.003 | 1.12E-11 | 350471 | 0.018 | 64.000 |
| Platelet crit | rs78235098 | 12 | 109969175 | T | C | 0.034 | 0.038 | 0.006 | 1.81E-09 | 350471 | 0.011 | 40.111 |
| Platelet crit | rs3184504 | 12 | 111884608 | C | T | 0.518 | -0.106 | 0.002 | 1.00E-200 | 350471 | 0.795 | 2808.984 |
| Platelet crit | rs11168249 | 12 | 48208368 | C | T | 0.46 | 0.023 | 0.002 | 2.19E-24 | 350471 | 0.038 | 132.249 |
| Platelet crit | rs76852203 | 12 | 46881929 | T | C | 0.067 | 0.035 | 0.005 | 6.98E-15 | 350471 | 0.014 | 49.000 |
| Platelet crit | rs56165099 | 12 | 21357731 | T | C | 0.183 | 0.016 | 0.003 | 2.13E-08 | 350471 | 0.008 | 28.444 |
| Platelet crit | rs804327 | 12 | 95862225 | C | T | 0.331 | 0.014 | 0.002 | 2.32E-08 | 350471 | 0.014 | 49.000 |
| Platelet crit | rs12425835 | 12 | 93903228 | T | C | 0.159 | 0.025 | 0.003 | 4.48E-16 | 350471 | 0.020 | 69.444 |
| Platelet crit | rs6489695 | 12 | 6255425 | G | A | 0.689 | -0.02 | 0.002 | 4.19E-15 | 350471 | 0.029 | 99.999 |
| Platelet crit | rs2607921 | 12 | 711496 | C | T | 0.601 | -0.016 | 0.002 | 4.00E-12 | 350471 | 0.018 | 64.000 |
| Platelet crit | rs7972367 | 12 | 26267648 | C | T | 0.675 | -0.014 | 0.002 | 1.43E-08 | 350471 | 0.014 | 49.000 |
| Platelet crit | rs670179 | 13 | 71236607 | A | T | 0.574 | 0.031 | 0.002 | 3.96E-40 | 350471 | 0.069 | 240.249 |
| Platelet crit | rs374039502 | 13 | 108960385 | A | T | 0.021 | -0.067 | 0.008 | 2.21E-15 | 350471 | 0.020 | 70.140 |
| Platelet crit | rs4773860 | 13 | 95901241 | T | C | 0.522 | 0.038 | 0.002 | 1.51E-60 | 350471 | 0.103 | 360.998 |
| Platelet crit | rs11618989 | 13 | 110489152 | T | C | 0.102 | -0.055 | 0.004 | 4.58E-49 | 350471 | 0.054 | 189.061 |
| Platelet crit | rs9590569 | 13 | 41584690 | C | T | 0.283 | -0.026 | 0.003 | 1.17E-25 | 350471 | 0.021 | 75.111 |
| Platelet crit | rs9572786 | 13 | 72342605 | G | A | 0.378 | -0.019 | 0.002 | 1.73E-15 | 350471 | 0.026 | 90.249 |
| Platelet crit | rs9521733 | 13 | 111034542 | C | T | 0.375 | -0.018 | 0.002 | 5.51E-14 | 350471 | 0.023 | 81.000 |
| Platelet crit | rs9535492 | 13 | 51384616 | T | C | 0.564 | -0.015 | 0.002 | 4.63E-11 | 350471 | 0.016 | 56.250 |
| Platelet crit | rs1924930 | 13 | 78447373 | A | T | 0.776 | -0.016 | 0.003 | 1.12E-08 | 350471 | 0.008 | 28.444 |
| Platelet crit | rs10145353 | 14 | 96204459 | G | A | 0.17 | -0.024 | 0.003 | 1.15E-15 | 350471 | 0.018 | 64.000 |
| Platelet crit | rs8017228 | 14 | 25449226 | C | A | 0.394 | 0.016 | 0.002 | 3.14E-12 | 350471 | 0.018 | 64.000 |
| Platelet crit | rs1033686 | 14 | 68362510 | A | G | 0.152 | -0.031 | 0.003 | 3.62E-22 | 350471 | 0.030 | 106.777 |
| Platelet crit | rs11160085 | 14 | 93112102 | C | T | 0.304 | -0.02 | 0.002 | 2.44E-16 | 350471 | 0.029 | 99.999 |
| Platelet crit | rs947001 | 14 | 104638441 | G | A | 0.673 | -0.014 | 0.002 | 3.99E-09 | 350471 | 0.014 | 49.000 |
| Platelet crit | rs12889267 | 14 | 21542766 | G | A | 0.168 | 0.017 | 0.003 | 1.64E-08 | 350471 | 0.009 | 32.111 |
| Platelet crit | rs34750619 | 14 | 93514882 | A | G | 0.07 | 0.042 | 0.004 | 1.93E-21 | 350471 | 0.031 | 110.249 |
| Platelet crit | rs12432021 | 14 | 81878043 | A | G | 0.651 | 0.014 | 0.002 | 4.13E-09 | 350471 | 0.014 | 49.000 |
| Platelet crit | rs1555405 | 14 | 101176769 | A | G | 0.248 | -0.061 | 0.003 | 1.14E-119 | 350471 | 0.118 | 413.442 |
| Platelet crit | rs2127870 | 14 | 65796846 | C | G | 0.791 | 0.03 | 0.003 | 2.83E-27 | 350471 | 0.029 | 99.999 |
| Platelet crit | rs194743 | 14 | 69286152 | C | T | 0.782 | -0.018 | 0.003 | 9.82E-11 | 350471 | 0.010 | 36.000 |
| Platelet crit | rs1057119 | 14 | 23746269 | G | C | 0.239 | -0.015 | 0.003 | 2.22E-08 | 350471 | 0.007 | 25.000 |
| Platelet crit | rs35258120 | 14 | 103058769 | A | G | 0.819 | 0.036 | 0.003 | 4.86E-35 | 350471 | 0.041 | 143.999 |
| Platelet crit | rs11549015 | 15 | 42235316 | T | C | 0.166 | 0.021 | 0.003 | 6.06E-12 | 350471 | 0.014 | 49.000 |
| Platelet crit | rs12708514 | 15 | 75366486 | G | A | 0.206 | -0.016 | 0.003 | 1.25E-08 | 350471 | 0.008 | 28.444 |
| Platelet crit | rs113513485 | 15 | 50606388 | T | A | 0.373 | -0.013 | 0.002 | 3.28E-08 | 350471 | 0.012 | 42.250 |
| Platelet crit | rs1719262 | 15 | 65185904 | A | T | 0.156 | 0.024 | 0.003 | 3.39E-14 | 350471 | 0.018 | 64.000 |
| Platelet crit | rs12910114 | 15 | 78530261 | T | C | 0.318 | 0.015 | 0.002 | 4.07E-10 | 350471 | 0.016 | 56.250 |
| Platelet crit | rs17507300 | 15 | 83722059 | G | A | 0.165 | -0.017 | 0.003 | 4.71E-08 | 350471 | 0.009 | 32.111 |
| Platelet crit | rs139974673 | 15 | 44027885 | C | T | 0.025 | 0.107 | 0.007 | 2.46E-48 | 350471 | 0.067 | 233.652 |
| Platelet crit | rs4965426 | 15 | 99248041 | A | G | 0.143 | -0.032 | 0.003 | 2.04E-22 | 350471 | 0.032 | 113.777 |
| Platelet crit | rs1982101 | 15 | 39300917 | T | A | 0.393 | -0.021 | 0.002 | 1.16E-18 | 350471 | 0.031 | 110.249 |
| Platelet crit | rs75395345 | 15 | 68373718 | C | G | 0.299 | 0.016 | 0.002 | 1.17E-10 | 350471 | 0.018 | 64.000 |
| Platelet crit | rs59865663 | 16 | 88558312 | A | G | 0.203 | 0.036 | 0.003 | 1.79E-36 | 350471 | 0.041 | 143.999 |
| Platelet crit | rs35666389 | 16 | 629458 | G | A | 0.43 | -0.016 | 0.002 | 1.51E-12 | 350471 | 0.018 | 64.000 |
| Platelet crit | rs11648192 | 16 | 28618708 | T | C | 0.435 | -0.016 | 0.002 | 2.11E-11 | 350471 | 0.018 | 64.000 |
| Platelet crit | rs56079926 | 16 | 30120372 | T | C | 0.348 | 0.014 | 0.002 | 3.95E-09 | 350471 | 0.014 | 49.000 |
| Platelet crit | rs4783187 | 16 | 85415838 | C | T | 0.877 | -0.038 | 0.003 | 3.86E-28 | 350471 | 0.046 | 160.444 |
| Platelet crit | rs4888387 | 16 | 75389755 | G | T | 0.595 | 0.019 | 0.002 | 1.85E-16 | 350471 | 0.026 | 90.249 |
| Platelet crit | rs12932413 | 16 | 4650551 | T | C | 0.161 | 0.018 | 0.003 | 5.70E-09 | 350471 | 0.010 | 36.000 |
| Platelet crit | rs918739 | 16 | 11439733 | T | G | 0.677 | -0.013 | 0.002 | 4.61E-08 | 350471 | 0.012 | 42.250 |
| Platelet crit | rs151234 | 16 | 28505660 | C | G | 0.129 | 0.038 | 0.003 | 3.07E-29 | 350471 | 0.046 | 160.444 |
| Platelet crit | rs113721422 | 16 | 530720 | A | G | 0.079 | 0.039 | 0.004 | 1.01E-20 | 350471 | 0.027 | 95.062 |
| Platelet crit | rs4785386 | 16 | 50164049 | A | G | 0.751 | 0.02 | 0.003 | 9.22E-14 | 350471 | 0.013 | 44.444 |
| Platelet crit | rs6499186 | 16 | 68660565 | T | C | 0.766 | -0.019 | 0.003 | 5.76E-13 | 350471 | 0.011 | 40.111 |
| Platelet crit | rs72807421 | 16 | 88590467 | T | C | 0.026 | -0.046 | 0.007 | 3.76E-10 | 350471 | 0.012 | 43.183 |
| Platelet crit | rs8049394 | 16 | 9037904 | A | G | 0.251 | -0.017 | 0.003 | 3.98E-11 | 350471 | 0.009 | 32.111 |
| Platelet crit | rs11150084 | 16 | 78561820 | G | C | 0.669 | 0.015 | 0.002 | 1.27E-09 | 350471 | 0.016 | 56.250 |
| Platelet crit | rs7185512 | 16 | 79713111 | G | A | 0.366 | -0.013 | 0.002 | 1.80E-08 | 350471 | 0.012 | 42.250 |
| Platelet crit | rs7220358 | 17 | 2023081 | G | A | 0.296 | -0.031 | 0.002 | 2.85E-36 | 350471 | 0.069 | 240.249 |
| Platelet crit | rs118083884 | 17 | 16522922 | A | G | 0.017 | -0.076 | 0.01 | 1.47E-14 | 350471 | 0.016 | 57.760 |
| Platelet crit | rs614973 | 17 | 18311854 | C | G | 0.499 | 0.014 | 0.002 | 1.95E-09 | 350471 | 0.014 | 49.000 |
| Platelet crit | rs9904395 | 17 | 55435275 | G | A | 0.04 | -0.075 | 0.006 | 2.94E-37 | 350471 | 0.045 | 156.249 |
| Platelet crit | rs150568286 | 17 | 42594755 | A | G | 0.012 | -0.096 | 0.01 | 1.65E-20 | 350471 | 0.026 | 92.159 |
| Platelet crit | rs11655709 | 17 | 56400617 | C | T | 0.104 | 0.024 | 0.004 | 7.45E-11 | 350471 | 0.010 | 36.000 |
| Platelet crit | rs8065615 | 17 | 57798165 | C | T | 0.295 | -0.016 | 0.002 | 2.72E-10 | 350471 | 0.018 | 64.000 |
| Platelet crit | rs7220821 | 17 | 61704147 | A | G | 0.275 | -0.015 | 0.003 | 1.05E-08 | 350471 | 0.007 | 25.000 |
| Platelet crit | rs61296342 | 17 | 29707521 | A | G | 0.548 | 0.013 | 0.002 | 1.41E-08 | 350471 | 0.012 | 42.250 |
| Platelet crit | rs4455005 | 17 | 1958536 | A | G | 0.64 | -0.037 | 0.002 | 3.90E-55 | 350471 | 0.098 | 342.248 |
| Platelet crit | rs2084312 | 17 | 72695211 | T | C | 0.8 | 0.02 | 0.003 | 7.16E-13 | 350471 | 0.013 | 44.444 |
| Platelet crit | rs12451471 | 17 | 78102517 | T | C | 0.374 | -0.016 | 0.002 | 3.63E-12 | 350471 | 0.018 | 64.000 |
| Platelet crit | rs12953087 | 17 | 59265201 | T | C | 0.796 | -0.017 | 0.003 | 1.89E-09 | 350471 | 0.009 | 32.111 |
| Platelet crit | rs7503168 | 17 | 33885904 | G | A | 0.172 | 0.041 | 0.003 | 6.96E-42 | 350471 | 0.053 | 186.777 |
| Platelet crit | rs113593735 | 17 | 35755546 | G | A | 0.362 | 0.022 | 0.002 | 2.27E-20 | 350471 | 0.035 | 120.999 |
| Platelet crit | rs9910058 | 17 | 76144314 | A | G | 0.54 | -0.018 | 0.002 | 3.29E-14 | 350471 | 0.023 | 81.000 |
| Platelet crit | rs73307474 | 17 | 42970500 | G | T | 0.068 | 0.032 | 0.005 | 6.53E-13 | 350471 | 0.012 | 40.960 |
| Platelet crit | rs113120049 | 17 | 17048374 | A | T | 0.018 | -0.052 | 0.009 | 1.16E-08 | 350471 | 0.010 | 33.383 |
| Platelet crit | rs8178824 | 17 | 64224775 | T | C | 0.03 | 0.108 | 0.007 | 9.76E-59 | 350471 | 0.068 | 238.039 |
| Platelet crit | rs12942540 | 17 | 4804073 | C | G | 0.135 | 0.031 | 0.003 | 4.32E-20 | 350471 | 0.030 | 106.777 |
| Platelet crit | rs8071462 | 17 | 739367 | A | G | 0.308 | -0.016 | 0.002 | 9.53E-11 | 350471 | 0.018 | 64.000 |
| Platelet crit | rs7215542 | 17 | 76368171 | A | G | 0.428 | -0.014 | 0.002 | 3.75E-09 | 350471 | 0.014 | 49.000 |
| Platelet crit | rs113232639 | 18 | 20715656 | A | G | 0.494 | -0.035 | 0.002 | 2.23E-50 | 350471 | 0.087 | 306.248 |
| Platelet crit | rs4121508 | 18 | 813554 | A | G | 0.306 | 0.016 | 0.002 | 2.49E-11 | 350471 | 0.018 | 64.000 |
| Platelet crit | rs11876150 | 18 | 67722760 | C | T | 0.948 | -0.029 | 0.005 | 9.30E-09 | 350471 | 0.010 | 33.640 |
| Platelet crit | rs7244202 | 18 | 67527701 | C | T | 0.475 | -0.027 | 0.002 | 2.93E-32 | 350471 | 0.052 | 182.249 |
| Platelet crit | rs8095635 | 18 | 22465767 | C | T | 0.087 | 0.026 | 0.004 | 2.17E-10 | 350471 | 0.012 | 42.250 |
| Platelet crit | rs2276392 | 18 | 13336841 | A | C | 0.289 | -0.014 | 0.003 | 9.08E-09 | 350471 | 0.006 | 21.778 |
| Platelet crit | rs632157 | 18 | 72911548 | A | G | 0.065 | -0.027 | 0.005 | 7.47E-09 | 350471 | 0.008 | 29.160 |
| Platelet crit | rs17758695 | 18 | 60920854 | T | C | 0.03 | -0.109 | 0.007 | 2.80E-59 | 350471 | 0.069 | 242.468 |
| Platelet crit | rs79010100 | 18 | 42005538 | C | T | 0.131 | 0.047 | 0.003 | 3.69E-43 | 350471 | 0.070 | 245.443 |
| Platelet crit | rs34654942 | 18 | 48970651 | A | T | 0.232 | -0.017 | 0.003 | 1.15E-10 | 350471 | 0.009 | 32.111 |
| Platelet crit | rs8093990 | 18 | 42122473 | C | A | 0.433 | 0.017 | 0.002 | 4.99E-14 | 350471 | 0.021 | 72.250 |
| Platelet crit | rs532252 | 18 | 60620764 | G | A | 0.404 | -0.014 | 0.002 | 5.47E-09 | 350471 | 0.014 | 49.000 |
| Platelet crit | rs117426264 | 18 | 56104751 | G | C | 0.021 | 0.047 | 0.008 | 7.70E-09 | 350471 | 0.010 | 34.515 |
| Platelet crit | rs61731111 | 19 | 3179517 | T | C | 0.011 | -0.087 | 0.011 | 2.12E-15 | 350471 | 0.018 | 62.553 |
| Platelet crit | rs3786877 | 19 | 38759180 | C | T | 0.447 | 0.015 | 0.002 | 1.83E-10 | 350471 | 0.016 | 56.250 |
| Platelet crit | rs12721051 | 19 | 45422160 | G | C | 0.19 | -0.034 | 0.003 | 6.17E-32 | 350471 | 0.037 | 128.444 |
| Platelet crit | rs58434384 | 19 | 19786099 | G | A | 0.086 | 0.06 | 0.004 | 7.50E-49 | 350471 | 0.064 | 224.999 |
| Platelet crit | rs34695688 | 19 | 39173645 | C | T | 0.071 | 0.031 | 0.004 | 3.03E-12 | 350471 | 0.017 | 60.062 |
| Platelet crit | rs8113810 | 19 | 58932106 | G | A | 0.485 | 0.015 | 0.002 | 3.19E-11 | 350471 | 0.016 | 56.250 |
| Platelet crit | rs12984096 | 19 | 17256798 | C | G | 0.391 | 0.026 | 0.002 | 2.34E-28 | 350471 | 0.048 | 168.999 |
| Platelet crit | rs2106446 | 19 | 36012084 | C | T | 0.667 | -0.018 | 0.002 | 2.00E-13 | 350471 | 0.023 | 81.000 |
| Platelet crit | rs36656 | 19 | 54664752 | C | A | 0.496 | 0.016 | 0.002 | 9.30E-12 | 350471 | 0.018 | 64.000 |
| Platelet crit | rs12985346 | 19 | 2164351 | A | T | 0.401 | -0.015 | 0.002 | 3.44E-10 | 350471 | 0.016 | 56.250 |
| Platelet crit | rs4807462 | 19 | 3408665 | T | A | 0.358 | -0.014 | 0.002 | 3.02E-09 | 350471 | 0.014 | 49.000 |
| Platelet crit | rs8108906 | 19 | 39365401 | A | G | 0.514 | 0.013 | 0.002 | 3.26E-08 | 350471 | 0.012 | 42.250 |
| Platelet crit | rs12459847 | 19 | 45751157 | C | G | 0.256 | -0.035 | 0.003 | 7.26E-41 | 350471 | 0.039 | 136.110 |
| Platelet crit | rs4807440 | 19 | 1026477 | T | G | 0.637 | 0.016 | 0.002 | 1.60E-11 | 350471 | 0.018 | 64.000 |
| Platelet crit | rs78744187 | 19 | 33754548 | T | C | 0.081 | -0.027 | 0.004 | 5.08E-11 | 350471 | 0.013 | 45.562 |
| Platelet crit | rs77757007 | 19 | 55685617 | T | A | 0.087 | 0.022 | 0.004 | 3.13E-08 | 350471 | 0.009 | 30.250 |
| Platelet crit | rs415064 | 20 | 57597971 | C | G | 0.047 | -0.152 | 0.005 | 6.25E-177 | 350471 | 0.263 | 924.155 |
| Platelet crit | rs156355 | 20 | 1818613 | C | T | 0.461 | 0.037 | 0.002 | 3.06E-56 | 350471 | 0.098 | 342.248 |
| Platelet crit | rs16979901 | 20 | 54988877 | G | A | 0.102 | 0.033 | 0.004 | 1.55E-18 | 350471 | 0.019 | 68.062 |
| Platelet crit | rs6103669 | 20 | 42816845 | A | G | 0.287 | -0.016 | 0.003 | 6.41E-11 | 350471 | 0.008 | 28.444 |
| Platelet crit | rs737092 | 20 | 55990405 | C | T | 0.487 | -0.013 | 0.002 | 4.10E-09 | 350471 | 0.012 | 42.250 |
| Platelet crit | rs67696533 | 20 | 31173362 | A | G | 0.311 | -0.014 | 0.002 | 6.95E-09 | 350471 | 0.014 | 49.000 |
| Platelet crit | rs11906768 | 20 | 1924066 | C | T | 0.266 | -0.075 | 0.003 | 8.09E-190 | 350471 | 0.178 | 624.996 |
| Platelet crit | rs80054178 | 20 | 30294682 | C | T | 0.022 | 0.131 | 0.008 | 5.78E-65 | 350471 | 0.076 | 268.139 |
| Platelet crit | rs62198914 | 20 | 8603581 | A | G | 0.173 | -0.029 | 0.003 | 1.94E-22 | 350471 | 0.027 | 93.444 |
| Platelet crit | rs112604138 | 20 | 62279344 | C | T | 0.57 | 0.021 | 0.002 | 1.41E-18 | 350471 | 0.031 | 110.249 |
| Platelet crit | rs6060978 | 20 | 30416893 | A | G | 0.216 | -0.032 | 0.003 | 5.43E-32 | 350471 | 0.032 | 113.777 |
| Platelet crit | rs118062269 | 20 | 1590770 | G | A | 0.777 | 0.021 | 0.003 | 2.72E-14 | 350471 | 0.014 | 49.000 |
| Platelet crit | rs73597777 | 20 | 16475474 | A | C | 0.292 | 0.017 | 0.003 | 2.04E-11 | 350471 | 0.009 | 32.111 |
| Platelet crit | rs1736144 | 21 | 16811482 | C | G | 0.429 | -0.022 | 0.002 | 2.41E-21 | 350471 | 0.035 | 120.999 |
| Platelet crit | rs1984021 | 21 | 39836204 | A | G | 0.136 | 0.042 | 0.003 | 2.76E-37 | 350471 | 0.056 | 195.999 |
| Platelet crit | rs11701383 | 21 | 36411387 | C | G | 0.074 | -0.046 | 0.004 | 5.11E-25 | 350471 | 0.038 | 132.249 |
| Platelet crit | rs2823025 | 21 | 16424421 | G | A | 0.094 | 0.027 | 0.004 | 1.96E-12 | 350471 | 0.013 | 45.562 |
| Platelet crit | rs73203043 | 21 | 36393747 | C | G | 0.072 | -0.046 | 0.004 | 2.48E-25 | 350471 | 0.038 | 132.249 |
| Platelet crit | rs2238784 | 22 | 19972559 | A | G | 0.286 | 0.024 | 0.003 | 5.20E-22 | 350471 | 0.018 | 64.000 |
| Platelet crit | rs12484795 | 22 | 44343626 | C | A | 0.173 | -0.024 | 0.003 | 6.91E-16 | 350471 | 0.018 | 64.000 |
| Platelet crit | rs5763079 | 22 | 29632258 | C | A | 0.523 | -0.015 | 0.002 | 2.02E-11 | 350471 | 0.016 | 56.250 |
| Platelet crit | rs56300819 | 22 | 30679303 | C | T | 0.242 | -0.024 | 0.003 | 9.15E-19 | 350471 | 0.018 | 64.000 |
| Platelet crit | rs75107793 | 22 | 50628937 | A | G | 0.073 | 0.106 | 0.004 | 8.67E-130 | 350471 | 0.200 | 702.246 |
| Platelet crit | rs855791 | 22 | 37462936 | G | A | 0.561 | -0.028 | 0.002 | 1.15E-34 | 350471 | 0.056 | 195.999 |
| Platelet crit | rs148762611 | 22 | 50769029 | G | A | 0.347 | -0.022 | 0.002 | 8.10E-20 | 350471 | 0.035 | 120.999 |
| Platelet distribution width | rs11204523 | 1 | 248020556 | C | G | 0.76 | -0.048 | 0.003 | 6.02E-67 | 350470 | 0.073 | 255.999 |
| Platelet distribution width | rs61851769 | 1 | 211803761 | T | C | 0.237 | 0.028 | 0.003 | 5.70E-23 | 350470 | 0.025 | 87.111 |
| Platelet distribution width | rs4845364 | 1 | 154141908 | G | A | 0.505 | -0.022 | 0.002 | 1.68E-21 | 350470 | 0.035 | 120.999 |
| Platelet distribution width | rs1275009 | 1 | 199070859 | T | C | 0.457 | -0.015 | 0.002 | 7.54E-10 | 350470 | 0.016 | 56.250 |
| Platelet distribution width | rs11497367 | 1 | 171996624 | A | G | 0.042 | 0.123 | 0.006 | 6.32E-97 | 350470 | 0.120 | 420.248 |
| Platelet distribution width | rs66530629 | 1 | 25032168 | A | G | 0.249 | 0.041 | 0.003 | 4.20E-50 | 350470 | 0.053 | 186.777 |
| Platelet distribution width | rs111631653 | 1 | 172417092 | A | T | 0.018 | 0.11 | 0.009 | 2.00E-33 | 350470 | 0.043 | 149.382 |
| Platelet distribution width | rs11120218 | 1 | 207278451 | A | G | 0.119 | 0.042 | 0.004 | 2.27E-30 | 350470 | 0.031 | 110.249 |
| Platelet distribution width | rs55710545 | 1 | 2231966 | C | G | 0.179 | -0.033 | 0.003 | 5.51E-26 | 350470 | 0.035 | 120.999 |
| Platelet distribution width | rs139435626 | 1 | 172561031 | C | A | 0.019 | -0.067 | 0.009 | 8.18E-14 | 350470 | 0.016 | 55.419 |
| Platelet distribution width | rs406428 | 1 | 112655624 | G | A | 0.754 | -0.019 | 0.003 | 8.13E-12 | 350470 | 0.011 | 40.111 |
| Platelet distribution width | rs141628682 | 1 | 171576210 | A | G | 0.025 | 0.044 | 0.008 | 1.85E-08 | 350470 | 0.009 | 30.250 |
| Platelet distribution width | rs6425522 | 1 | 171946082 | C | T | 0.802 | 0.165 | 0.003 | 1.00E-200 | 350470 | 0.856 | 3024.983 |
| Platelet distribution width | rs72660908 | 1 | 25583610 | G | C | 0.407 | 0.023 | 0.002 | 7.61E-22 | 350470 | 0.038 | 132.249 |
| Platelet distribution width | rs6671349 | 1 | 35256386 | C | T | 0.35 | 0.024 | 0.002 | 8.51E-22 | 350470 | 0.041 | 143.999 |
| Platelet distribution width | rs11121016 | 1 | 7804515 | G | A | 0.721 | -0.024 | 0.003 | 3.82E-19 | 350470 | 0.018 | 64.000 |
| Platelet distribution width | rs640527 | 1 | 95705615 | A | G | 0.92 | 0.034 | 0.004 | 6.88E-15 | 350470 | 0.021 | 72.250 |
| Platelet distribution width | rs145883541 | 1 | 115561248 | T | C | 0.077 | -0.029 | 0.005 | 8.52E-11 | 350470 | 0.010 | 33.640 |
| Platelet distribution width | rs7520479 | 1 | 92699339 | G | T | 0.441 | 0.013 | 0.002 | 3.70E-08 | 350470 | 0.012 | 42.250 |
| Platelet distribution width | rs2077725 | 1 | 52293570 | A | G | 0.609 | -0.013 | 0.002 | 4.00E-08 | 350470 | 0.012 | 42.250 |
| Platelet distribution width | rs56043070 | 1 | 247719769 | A | G | 0.072 | 0.137 | 0.005 | 3.61E-196 | 350470 | 0.214 | 750.756 |
| Platelet distribution width | rs12046329 | 1 | 113207531 | C | T | 0.526 | 0.046 | 0.002 | 6.51E-85 | 350470 | 0.151 | 528.997 |
| Platelet distribution width | rs631106 | 1 | 62901807 | A | C | 0.355 | -0.048 | 0.002 | 9.51E-85 | 350470 | 0.164 | 575.997 |
| Platelet distribution width | rs2789422 | 1 | 159892088 | A | G | 0.417 | 0.024 | 0.002 | 8.32E-23 | 350470 | 0.041 | 143.999 |
| Platelet distribution width | rs72688461 | 1 | 46111440 | T | G | 0.325 | 0.02 | 0.003 | 1.29E-14 | 350470 | 0.013 | 44.444 |
| Platelet distribution width | rs2564746 | 1 | 236728333 | T | G | 0.746 | 0.019 | 0.003 | 1.56E-12 | 350470 | 0.011 | 40.111 |
| Platelet distribution width | rs703156 | 1 | 156884103 | A | T | 0.159 | 0.022 | 0.003 | 7.71E-12 | 350470 | 0.015 | 53.777 |
| Platelet distribution width | rs3557 | 1 | 161188893 | G | T | 0.09 | -0.056 | 0.004 | 3.20E-41 | 350470 | 0.056 | 195.999 |
| Platelet distribution width | rs11204539 | 1 | 248046552 | C | G | 0.067 | 0.06 | 0.005 | 2.34E-36 | 350470 | 0.041 | 143.999 |
| Platelet distribution width | rs6681849 | 1 | 94874521 | T | G | 0.422 | -0.03 | 0.002 | 1.68E-35 | 350470 | 0.064 | 224.999 |
| Platelet distribution width | rs3767812 | 1 | 118155620 | A | G | 0.221 | -0.035 | 0.003 | 8.34E-35 | 350470 | 0.039 | 136.110 |
| Platelet distribution width | rs12564806 | 1 | 45924230 | T | C | 0.237 | 0.029 | 0.003 | 3.22E-25 | 350470 | 0.027 | 93.444 |
| Platelet distribution width | rs61822563 | 1 | 205076217 | T | C | 0.227 | 0.019 | 0.003 | 1.76E-11 | 350470 | 0.011 | 40.111 |
| Platelet distribution width | rs4926635 | 1 | 54887744 | T | C | 0.478 | 0.015 | 0.002 | 2.92E-10 | 350470 | 0.016 | 56.250 |
| Platelet distribution width | rs2295367 | 1 | 28224312 | T | C | 0.376 | -0.014 | 0.002 | 6.41E-09 | 350470 | 0.014 | 49.000 |
| Platelet distribution width | rs62191340 | 2 | 242645466 | A | G | 0.539 | 0.035 | 0.002 | 3.06E-50 | 350470 | 0.087 | 306.248 |
| Platelet distribution width | rs7608356 | 2 | 219201667 | T | C | 0.261 | -0.032 | 0.003 | 2.05E-31 | 350470 | 0.032 | 113.777 |
| Platelet distribution width | rs2723248 | 2 | 171873251 | G | A | 0.077 | -0.04 | 0.004 | 3.43E-19 | 350470 | 0.029 | 99.999 |
| Platelet distribution width | rs3204 | 2 | 24252436 | G | A | 0.46 | 0.015 | 0.002 | 8.65E-11 | 350470 | 0.016 | 56.250 |
| Platelet distribution width | rs4549095 | 2 | 121016474 | G | A | 0.698 | 0.016 | 0.003 | 1.07E-09 | 350470 | 0.008 | 28.444 |
| Platelet distribution width | rs2632372 | 2 | 169717541 | C | T | 0.503 | -0.013 | 0.002 | 2.08E-08 | 350470 | 0.012 | 42.250 |
| Platelet distribution width | rs655029 | 2 | 31477838 | A | G | 0.708 | -0.131 | 0.003 | 1.00E-200 | 350470 | 0.541 | 1906.767 |
| Platelet distribution width | rs60696641 | 2 | 225742080 | A | G | 0.079 | 0.126 | 0.004 | 5.62E-182 | 350470 | 0.282 | 992.244 |
| Platelet distribution width | rs78909033 | 2 | 241510903 | A | G | 0.135 | -0.047 | 0.003 | 8.73E-42 | 350470 | 0.070 | 245.443 |
| Platelet distribution width | rs79716587 | 2 | 143886819 | A | G | 0.127 | 0.045 | 0.004 | 1.44E-36 | 350470 | 0.036 | 126.562 |
| Platelet distribution width | rs1448319 | 2 | 223980125 | G | A | 0.945 | -0.063 | 0.005 | 4.89E-34 | 350470 | 0.045 | 158.759 |
| Platelet distribution width | rs7563866 | 2 | 65331078 | G | A | 0.698 | 0.016 | 0.003 | 3.22E-10 | 350470 | 0.008 | 28.444 |
| Platelet distribution width | rs1532675 | 2 | 106333764 | C | T | 0.1 | 0.024 | 0.004 | 8.19E-10 | 350470 | 0.010 | 36.000 |
| Platelet distribution width | rs11674612 | 2 | 85658232 | G | A | 0.364 | 0.014 | 0.002 | 5.81E-09 | 350470 | 0.014 | 49.000 |
| Platelet distribution width | rs13033725 | 2 | 68578058 | G | C | 0.331 | 0.024 | 0.003 | 3.79E-22 | 350470 | 0.018 | 64.000 |
| Platelet distribution width | rs12997003 | 2 | 106436413 | T | C | 0.384 | -0.022 | 0.002 | 8.39E-19 | 350470 | 0.035 | 120.999 |
| Platelet distribution width | rs715 | 2 | 211543055 | C | T | 0.312 | 0.022 | 0.003 | 1.60E-17 | 350470 | 0.015 | 53.777 |
| Platelet distribution width | rs7570428 | 2 | 11307634 | C | G | 0.566 | -0.017 | 0.002 | 4.78E-13 | 350470 | 0.021 | 72.250 |
| Platelet distribution width | rs1373218 | 2 | 31425783 | G | A | 0.547 | 0.016 | 0.002 | 5.56E-12 | 350470 | 0.018 | 64.000 |
| Platelet distribution width | rs73934253 | 2 | 62728740 | G | A | 0.109 | -0.025 | 0.004 | 3.34E-11 | 350470 | 0.011 | 39.062 |
| Platelet distribution width | rs950965 | 2 | 66668309 | G | A | 0.66 | -0.016 | 0.002 | 5.98E-11 | 350470 | 0.018 | 64.000 |
| Platelet distribution width | rs796063 | 2 | 101729443 | T | C | 0.468 | -0.015 | 0.002 | 4.99E-10 | 350470 | 0.016 | 56.250 |
| Platelet distribution width | rs1878202 | 2 | 189841916 | T | C | 0.268 | 0.017 | 0.003 | 6.27E-10 | 350470 | 0.009 | 32.111 |
| Platelet distribution width | rs7574937 | 2 | 220099331 | T | C | 0.097 | 0.069 | 0.004 | 1.89E-67 | 350470 | 0.085 | 297.561 |
| Platelet distribution width | rs2705730 | 2 | 183963690 | T | C | 0.833 | -0.029 | 0.003 | 3.63E-20 | 350470 | 0.027 | 93.444 |
| Platelet distribution width | rs11123325 | 2 | 111832215 | A | G | 0.859 | -0.021 | 0.003 | 7.19E-10 | 350470 | 0.014 | 49.000 |
| Platelet distribution width | rs1271272 | 2 | 207955477 | A | G | 0.312 | -0.015 | 0.003 | 3.85E-09 | 350470 | 0.007 | 25.000 |
| Platelet distribution width | rs606552 | 3 | 124441501 | G | A | 0.247 | 0.06 | 0.003 | 7.40E-107 | 350470 | 0.114 | 399.998 |
| Platelet distribution width | rs2175740 | 3 | 124377165 | A | G | 0.156 | 0.027 | 0.003 | 6.22E-17 | 350470 | 0.023 | 81.000 |
| Platelet distribution width | rs997680 | 3 | 27443638 | A | G | 0.267 | 0.022 | 0.003 | 4.37E-16 | 350470 | 0.015 | 53.777 |
| Platelet distribution width | rs4678938 | 3 | 37181015 | C | G | 0.468 | -0.019 | 0.002 | 5.23E-16 | 350470 | 0.026 | 90.249 |
| Platelet distribution width | rs7649045 | 3 | 196519878 | C | T | 0.594 | 0.013 | 0.002 | 3.51E-08 | 350470 | 0.012 | 42.250 |
| Platelet distribution width | rs7639048 | 3 | 183746044 | G | A | 0.489 | -0.021 | 0.002 | 1.08E-18 | 350470 | 0.031 | 110.249 |
| Platelet distribution width | rs7372674 | 3 | 196149165 | A | C | 0.358 | 0.015 | 0.002 | 7.26E-10 | 350470 | 0.016 | 56.250 |
| Platelet distribution width | rs9844271 | 3 | 185761846 | T | G | 0.858 | -0.02 | 0.003 | 2.00E-09 | 350470 | 0.013 | 44.444 |
| Platelet distribution width | rs13320485 | 3 | 136664737 | T | C | 0.016 | -0.053 | 0.009 | 9.25E-09 | 350470 | 0.010 | 34.679 |
| Platelet distribution width | rs6796837 | 3 | 39076188 | T | C | 0.304 | -0.02 | 0.003 | 2.32E-15 | 350470 | 0.013 | 44.444 |
| Platelet distribution width | rs6443617 | 3 | 178758534 | G | A | 0.75 | 0.019 | 0.003 | 1.54E-12 | 350470 | 0.011 | 40.111 |
| Platelet distribution width | rs55689339 | 3 | 50308040 | G | C | 0.063 | -0.032 | 0.005 | 3.01E-10 | 350470 | 0.012 | 40.960 |
| Platelet distribution width | rs111430408 | 3 | 100848597 | T | C | 0.071 | 0.026 | 0.005 | 3.05E-08 | 350470 | 0.008 | 27.040 |
| Platelet distribution width | rs6808932 | 3 | 113309149 | C | A | 0.386 | 0.013 | 0.002 | 4.12E-08 | 350470 | 0.012 | 42.250 |
| Platelet distribution width | rs3804749 | 3 | 122833003 | T | C | 0.593 | 0.04 | 0.002 | 1.35E-60 | 350470 | 0.114 | 399.998 |
| Platelet distribution width | rs7640885 | 3 | 15741439 | C | A | 0.668 | -0.023 | 0.003 | 5.96E-20 | 350470 | 0.017 | 58.777 |
| Platelet distribution width | rs1466733 | 3 | 194120998 | G | A | 0.236 | 0.024 | 0.003 | 6.82E-18 | 350470 | 0.018 | 64.000 |
| Platelet distribution width | rs73168819 | 3 | 149061733 | A | G | 0.407 | -0.017 | 0.002 | 5.28E-13 | 350470 | 0.021 | 72.250 |
| Platelet distribution width | rs10936568 | 3 | 168737002 | T | C | 0.97 | 0.044 | 0.007 | 9.22E-10 | 350470 | 0.011 | 39.510 |
| Platelet distribution width | rs62246446 | 3 | 47185650 | G | C | 0.176 | 0.049 | 0.003 | 2.18E-55 | 350470 | 0.076 | 266.776 |
| Platelet distribution width | rs56210800 | 3 | 124472592 | G | C | 0.132 | -0.03 | 0.003 | 4.90E-18 | 350470 | 0.029 | 99.999 |
| Platelet distribution width | rs1801282 | 3 | 12393125 | G | C | 0.12 | -0.026 | 0.004 | 3.42E-13 | 350470 | 0.012 | 42.250 |
| Platelet distribution width | rs1219275 | 4 | 143084950 | C | T | 0.797 | 0.018 | 0.003 | 6.57E-10 | 350470 | 0.010 | 36.000 |
| Platelet distribution width | rs11731274 | 4 | 6891455 | G | T | 0.176 | -0.064 | 0.003 | 1.01E-94 | 350470 | 0.130 | 455.109 |
| Platelet distribution width | rs12640722 | 4 | 48112291 | T | A | 0.7 | 0.023 | 0.003 | 2.28E-18 | 350470 | 0.017 | 58.777 |
| Platelet distribution width | rs17296412 | 4 | 120656753 | A | C | 0.246 | -0.016 | 0.003 | 8.14E-09 | 350470 | 0.008 | 28.444 |
| Platelet distribution width | rs7696969 | 4 | 143326714 | G | T | 0.619 | -0.037 | 0.002 | 2.28E-51 | 350470 | 0.098 | 342.248 |
| Platelet distribution width | rs7692976 | 4 | 110911567 | G | A | 0.404 | 0.021 | 0.002 | 8.39E-18 | 350470 | 0.031 | 110.249 |
| Platelet distribution width | rs2645686 | 4 | 77880423 | T | A | 0.607 | 0.016 | 0.002 | 8.94E-11 | 350470 | 0.018 | 64.000 |
| Platelet distribution width | rs13107325 | 4 | 103188709 | T | C | 0.075 | -0.047 | 0.004 | 2.37E-25 | 350470 | 0.039 | 138.062 |
| Platelet distribution width | rs62291089 | 4 | 6910335 | G | C | 0.195 | 0.022 | 0.003 | 7.69E-12 | 350470 | 0.015 | 53.777 |
| Platelet distribution width | rs62379580 | 5 | 159611348 | C | T | 0.666 | 0.034 | 0.003 | 5.88E-43 | 350470 | 0.037 | 128.444 |
| Platelet distribution width | rs16875506 | 5 | 77788791 | G | A | 0.296 | 0.035 | 0.003 | 2.45E-41 | 350470 | 0.039 | 136.110 |
| Platelet distribution width | rs464605 | 5 | 55807370 | T | C | 0.746 | 0.02 | 0.003 | 5.85E-12 | 350470 | 0.013 | 44.444 |
| Platelet distribution width | rs2057826 | 5 | 132142232 | C | T | 0.299 | -0.017 | 0.003 | 2.64E-10 | 350470 | 0.009 | 32.111 |
| Platelet distribution width | rs6556405 | 5 | 158635102 | C | T | 0.249 | 0.031 | 0.003 | 7.26E-30 | 350470 | 0.030 | 106.777 |
| Platelet distribution width | rs72814539 | 5 | 157279508 | T | C | 0.063 | 0.034 | 0.005 | 4.89E-12 | 350470 | 0.013 | 46.240 |
| Platelet distribution width | rs17463814 | 5 | 39122983 | A | G | 0.221 | -0.019 | 0.003 | 1.84E-11 | 350470 | 0.011 | 40.111 |
| Platelet distribution width | rs2303127 | 5 | 52355854 | C | T | 0.403 | -0.014 | 0.002 | 6.09E-09 | 350470 | 0.014 | 49.000 |
| Platelet distribution width | rs10036404 | 5 | 176775385 | G | C | 0.023 | -0.046 | 0.008 | 1.07E-08 | 350470 | 0.009 | 33.062 |
| Platelet distribution width | rs35188965 | 5 | 1104938 | T | C | 0.582 | -0.029 | 0.002 | 1.27E-33 | 350470 | 0.060 | 210.249 |
| Platelet distribution width | rs114694170 | 5 | 88180196 | C | T | 0.059 | -0.119 | 0.005 | 1.42E-122 | 350470 | 0.161 | 566.437 |
| Platelet distribution width | rs4099245 | 5 | 61540257 | A | G | 0.414 | -0.017 | 0.002 | 4.65E-13 | 350470 | 0.021 | 72.250 |
| Platelet distribution width | rs4958244 | 5 | 133845380 | G | A | 0.58 | -0.041 | 0.002 | 2.05E-65 | 350470 | 0.120 | 420.248 |
| Platelet distribution width | rs254415 | 5 | 77071703 | T | G | 0.304 | 0.032 | 0.003 | 1.22E-35 | 350470 | 0.032 | 113.777 |
| Platelet distribution width | rs6878741 | 5 | 75715186 | G | T | 0.727 | 0.023 | 0.003 | 9.98E-17 | 350470 | 0.017 | 58.777 |
| Platelet distribution width | rs1840806 | 5 | 78554137 | T | G | 0.387 | -0.018 | 0.002 | 1.03E-12 | 350470 | 0.023 | 81.000 |
| Platelet distribution width | rs62332588 | 5 | 1289880 | C | T | 0.602 | -0.014 | 0.003 | 1.77E-08 | 350470 | 0.006 | 21.778 |
| Platelet distribution width | rs12209838 | 6 | 122295244 | T | G | 0.161 | 0.022 | 0.003 | 1.45E-11 | 350470 | 0.015 | 53.777 |
| Platelet distribution width | rs1413700 | 6 | 7216484 | C | G | 0.236 | 0.015 | 0.003 | 3.44E-08 | 350470 | 0.007 | 25.000 |
| Platelet distribution width | rs6905288 | 6 | 43758873 | A | G | 0.567 | 0.018 | 0.002 | 2.01E-14 | 350470 | 0.023 | 81.000 |
| Platelet distribution width | rs9264277 | 6 | 31224667 | C | T | 0.632 | -0.016 | 0.002 | 1.81E-10 | 350470 | 0.018 | 64.000 |
| Platelet distribution width | rs7768878 | 6 | 152995543 | A | G | 0.028 | 0.046 | 0.007 | 2.21E-10 | 350470 | 0.012 | 43.183 |
| Platelet distribution width | rs76237797 | 6 | 47919053 | A | G | 0.014 | 0.063 | 0.01 | 7.19E-10 | 350470 | 0.011 | 39.690 |
| Platelet distribution width | rs688181 | 6 | 158092638 | C | T | 0.719 | 0.038 | 0.003 | 1.89E-46 | 350470 | 0.046 | 160.444 |
| Platelet distribution width | rs13340461 | 6 | 41924278 | T | C | 0.273 | 0.027 | 0.003 | 1.60E-23 | 350470 | 0.023 | 81.000 |
| Platelet distribution width | rs2326842 | 6 | 6926071 | T | C | 0.195 | -0.023 | 0.003 | 9.00E-15 | 350470 | 0.017 | 58.777 |
| Platelet distribution width | rs6912933 | 6 | 135627534 | A | G | 0.528 | -0.016 | 0.002 | 8.86E-12 | 350470 | 0.018 | 64.000 |
| Platelet distribution width | rs6927870 | 6 | 31410131 | G | A | 0.243 | -0.047 | 0.003 | 7.72E-65 | 350470 | 0.070 | 245.443 |
| Platelet distribution width | rs2462506 | 6 | 71347062 | A | G | 0.285 | 0.023 | 0.003 | 7.37E-19 | 350470 | 0.017 | 58.777 |
| Platelet distribution width | rs12202135 | 6 | 158422080 | T | C | 0.129 | -0.03 | 0.004 | 1.34E-16 | 350470 | 0.016 | 56.250 |
| Platelet distribution width | rs9461908 | 6 | 33736082 | A | G | 0.055 | 0.03 | 0.005 | 6.36E-09 | 350470 | 0.010 | 36.000 |
| Platelet distribution width | rs1980532 | 6 | 110720209 | G | A | 0.447 | -0.056 | 0.002 | 1.47E-121 | 350470 | 0.223 | 783.996 |
| Platelet distribution width | rs214065 | 6 | 25540927 | A | G | 0.428 | 0.042 | 0.002 | 2.44E-68 | 350470 | 0.126 | 440.997 |
| Platelet distribution width | rs4711890 | 6 | 47606712 | G | C | 0.267 | -0.041 | 0.003 | 3.23E-53 | 350470 | 0.053 | 186.777 |
| Platelet distribution width | rs2327531 | 6 | 135048564 | G | A | 0.209 | -0.022 | 0.003 | 5.93E-14 | 350470 | 0.015 | 53.777 |
| Platelet distribution width | rs7747978 | 6 | 36525321 | A | G | 0.014 | -0.073 | 0.01 | 7.48E-13 | 350470 | 0.015 | 53.290 |
| Platelet distribution width | rs2153960 | 6 | 108988184 | A | G | 0.712 | -0.018 | 0.003 | 3.22E-12 | 350470 | 0.010 | 36.000 |
| Platelet distribution width | rs114760566 | 6 | 34192036 | A | C | 0.046 | 0.037 | 0.006 | 8.95E-11 | 350470 | 0.011 | 38.028 |
| Platelet distribution width | rs4726889 | 7 | 140232243 | A | G | 0.462 | 0.017 | 0.002 | 4.26E-12 | 350470 | 0.021 | 72.250 |
| Platelet distribution width | rs17138723 | 7 | 18217017 | G | C | 0.217 | 0.02 | 0.003 | 4.98E-12 | 350470 | 0.013 | 44.444 |
| Platelet distribution width | rs7777102 | 7 | 73058017 | G | A | 0.134 | -0.024 | 0.003 | 8.49E-12 | 350470 | 0.018 | 64.000 |
| Platelet distribution width | rs57556994 | 7 | 135667470 | C | T | 0.075 | -0.048 | 0.004 | 2.63E-26 | 350470 | 0.041 | 143.999 |
| Platelet distribution width | rs6952260 | 7 | 37486477 | A | T | 0.249 | 0.025 | 0.003 | 1.21E-19 | 350470 | 0.020 | 69.444 |
| Platelet distribution width | rs1870028 | 7 | 50258313 | T | C | 0.075 | 0.033 | 0.005 | 1.71E-13 | 350470 | 0.012 | 43.560 |
| Platelet distribution width | rs78000359 | 7 | 115552090 | C | A | 0.024 | -0.042 | 0.008 | 4.69E-08 | 350470 | 0.008 | 27.562 |
| Platelet distribution width | rs342293 | 7 | 106372219 | G | C | 0.459 | 0.105 | 0.002 | 1.00E-200 | 350470 | 0.780 | 2756.234 |
| Platelet distribution width | rs4721764 | 7 | 2578455 | A | G | 0.214 | -0.023 | 0.003 | 4.44E-15 | 350470 | 0.017 | 58.777 |
| Platelet distribution width | rs314326 | 7 | 100437397 | G | C | 0.531 | -0.018 | 0.002 | 1.60E-14 | 350470 | 0.023 | 81.000 |
| Platelet distribution width | rs1182197 | 7 | 2863289 | C | A | 0.378 | -0.018 | 0.002 | 5.83E-14 | 350470 | 0.023 | 81.000 |
| Platelet distribution width | rs10275592 | 7 | 129248502 | C | T | 0.912 | 0.029 | 0.004 | 3.54E-12 | 350470 | 0.015 | 52.562 |
| Platelet distribution width | rs7456677 | 7 | 80100568 | T | C | 0.953 | -0.032 | 0.006 | 2.51E-08 | 350470 | 0.008 | 28.444 |
| Platelet distribution width | rs13224082 | 7 | 116515781 | T | A | 0.242 | 0.038 | 0.003 | 1.93E-42 | 350470 | 0.046 | 160.444 |
| Platelet distribution width | rs7808005 | 7 | 37457359 | T | C | 0.279 | 0.032 | 0.003 | 1.09E-33 | 350470 | 0.032 | 113.777 |
| Platelet distribution width | rs13233747 | 7 | 73149302 | A | G | 0.347 | -0.029 | 0.002 | 4.12E-32 | 350470 | 0.060 | 210.249 |
| Platelet distribution width | rs2278073 | 7 | 134030819 | G | A | 0.462 | 0.019 | 0.002 | 1.45E-15 | 350470 | 0.026 | 90.249 |
| Platelet distribution width | rs896852 | 8 | 95960886 | T | G | 0.511 | -0.019 | 0.002 | 1.12E-15 | 350470 | 0.026 | 90.249 |
| Platelet distribution width | rs4425772 | 8 | 19854500 | T | C | 0.264 | -0.037 | 0.003 | 1.66E-43 | 350470 | 0.043 | 152.110 |
| Platelet distribution width | rs7002892 | 8 | 131235135 | T | A | 0.498 | -0.018 | 0.002 | 4.07E-14 | 350470 | 0.023 | 81.000 |
| Platelet distribution width | rs3748136 | 8 | 9030160 | A | G | 0.211 | 0.087 | 0.003 | 3.73E-198 | 350470 | 0.239 | 840.995 |
| Platelet distribution width | rs6993770 | 8 | 106581528 | T | A | 0.286 | 0.059 | 0.003 | 1.45E-112 | 350470 | 0.110 | 386.776 |
| Platelet distribution width | rs73197706 | 8 | 8881024 | G | C | 0.048 | 0.051 | 0.006 | 8.08E-20 | 350470 | 0.021 | 72.250 |
| Platelet distribution width | rs6558407 | 8 | 144995494 | T | C | 0.424 | -0.048 | 0.002 | 1.95E-91 | 350470 | 0.164 | 575.997 |
| Platelet distribution width | rs2321235 | 8 | 25215103 | G | A | 0.414 | 0.025 | 0.002 | 7.11E-24 | 350470 | 0.045 | 156.249 |
| Platelet distribution width | rs10808546 | 8 | 126495818 | T | C | 0.449 | -0.018 | 0.002 | 1.11E-14 | 350470 | 0.023 | 81.000 |
| Platelet distribution width | rs6994859 | 8 | 25249772 | A | G | 0.363 | -0.018 | 0.002 | 8.39E-13 | 350470 | 0.023 | 81.000 |
| Platelet distribution width | rs6999008 | 8 | 56768236 | A | G | 0.444 | 0.015 | 0.002 | 8.07E-11 | 350470 | 0.016 | 56.250 |
| Platelet distribution width | rs73205782 | 8 | 13261462 | G | T | 0.204 | -0.019 | 0.003 | 2.04E-10 | 350470 | 0.011 | 40.111 |
| Platelet distribution width | rs62527185 | 8 | 106497511 | T | C | 0.023 | -0.044 | 0.008 | 2.88E-08 | 350470 | 0.009 | 30.250 |
| Platelet distribution width | rs10820606 | 9 | 99192919 | C | A | 0.228 | -0.06 | 0.003 | 1.73E-96 | 350470 | 0.114 | 399.998 |
| Platelet distribution width | rs507666 | 9 | 136149399 | A | G | 0.184 | -0.028 | 0.003 | 1.33E-20 | 350470 | 0.025 | 87.111 |
| Platelet distribution width | rs10993693 | 9 | 93557698 | G | A | 0.435 | 0.017 | 0.002 | 4.92E-13 | 350470 | 0.021 | 72.250 |
| Platelet distribution width | rs62543170 | 9 | 73070001 | G | A | 0.436 | 0.024 | 0.002 | 1.58E-24 | 350470 | 0.041 | 143.999 |
| Platelet distribution width | rs72766630 | 9 | 136926791 | T | G | 0.164 | -0.023 | 0.003 | 1.17E-12 | 350470 | 0.017 | 58.777 |
| Platelet distribution width | rs4744227 | 9 | 96126075 | T | C | 0.461 | 0.016 | 0.002 | 4.21E-11 | 350470 | 0.018 | 64.000 |
| Platelet distribution width | rs61751937 | 9 | 113312231 | C | G | 0.029 | 0.082 | 0.007 | 1.71E-31 | 350470 | 0.039 | 137.224 |
| Platelet distribution width | rs62550976 | 9 | 91417873 | T | C | 0.067 | 0.041 | 0.005 | 3.13E-18 | 350470 | 0.019 | 67.240 |
| Platelet distribution width | rs7024673 | 9 | 112790036 | T | C | 0.315 | -0.017 | 0.003 | 2.86E-11 | 350470 | 0.009 | 32.111 |
| Platelet distribution width | rs619874 | 9 | 79301693 | C | T | 0.615 | 0.016 | 0.002 | 1.93E-10 | 350470 | 0.018 | 64.000 |
| Platelet distribution width | rs10984241 | 9 | 100661712 | A | G | 0.218 | -0.031 | 0.003 | 5.43E-26 | 350470 | 0.030 | 106.777 |
| Platelet distribution width | rs142916631 | 9 | 135589430 | A | C | 0.023 | 0.068 | 0.009 | 1.53E-15 | 350470 | 0.016 | 57.086 |
| Platelet distribution width | rs10811664 | 9 | 22142907 | A | G | 0.155 | 0.019 | 0.003 | 3.37E-09 | 350470 | 0.011 | 40.111 |
| Platelet distribution width | rs636918 | 9 | 274641 | C | T | 0.623 | 0.051 | 0.002 | 4.47E-94 | 350470 | 0.185 | 650.246 |
| Platelet distribution width | rs760402 | 9 | 35686376 | A | G | 0.688 | 0.023 | 0.003 | 1.11E-19 | 350470 | 0.017 | 58.777 |
| Platelet distribution width | rs111425801 | 9 | 135970191 | A | G | 0.036 | 0.053 | 0.006 | 4.82E-17 | 350470 | 0.022 | 78.027 |
| Platelet distribution width | rs77632732 | 9 | 434519 | G | A | 0.009 | 0.094 | 0.012 | 4.19E-14 | 350470 | 0.018 | 61.361 |
| Platelet distribution width | rs10904896 | 10 | 17225826 | A | G | 0.532 | -0.02 | 0.002 | 2.16E-17 | 350470 | 0.029 | 99.999 |
| Platelet distribution width | rs2429511 | 10 | 115801253 | C | T | 0.479 | -0.018 | 0.002 | 8.64E-14 | 350470 | 0.023 | 81.000 |
| Platelet distribution width | rs10736152 | 10 | 102880414 | C | G | 0.512 | -0.016 | 0.002 | 2.13E-11 | 350470 | 0.018 | 64.000 |
| Platelet distribution width | rs835259 | 10 | 94782735 | G | T | 0.475 | -0.014 | 0.002 | 4.05E-09 | 350470 | 0.014 | 49.000 |
| Platelet distribution width | rs7073010 | 10 | 97440812 | T | C | 0.35 | -0.014 | 0.002 | 7.94E-09 | 350470 | 0.014 | 49.000 |
| Platelet distribution width | rs11013176 | 10 | 23177471 | G | A | 0.281 | -0.023 | 0.003 | 1.62E-18 | 350470 | 0.017 | 58.777 |
| Platelet distribution width | rs2767627 | 10 | 30251648 | A | C | 0.453 | -0.016 | 0.002 | 6.68E-11 | 350470 | 0.018 | 64.000 |
| Platelet distribution width | rs10886430 | 10 | 121010256 | G | A | 0.127 | 0.038 | 0.004 | 1.01E-25 | 350470 | 0.026 | 90.249 |
| Platelet distribution width | rs10906697 | 10 | 14599229 | G | T | 0.539 | 0.024 | 0.002 | 1.29E-23 | 350470 | 0.041 | 143.999 |
| Platelet distribution width | rs11598602 | 10 | 32667121 | A | G | 0.184 | -0.026 | 0.003 | 1.16E-17 | 350470 | 0.021 | 75.111 |
| Platelet distribution width | rs1797077 | 10 | 16853252 | T | C | 0.48 | 0.034 | 0.002 | 8.02E-47 | 350470 | 0.082 | 288.998 |
| Platelet distribution width | rs7896518 | 10 | 65104500 | G | A | 0.427 | -0.03 | 0.002 | 1.29E-35 | 350470 | 0.064 | 224.999 |
| Platelet distribution width | rs7083045 | 10 | 64539964 | G | A | 0.515 | -0.014 | 0.002 | 1.23E-09 | 350470 | 0.014 | 49.000 |
| Platelet distribution width | rs17776046 | 10 | 61412201 | A | G | 0.371 | 0.014 | 0.002 | 7.60E-09 | 350470 | 0.014 | 49.000 |
| Platelet distribution width | rs45563932 | 10 | 50374930 | T | C | 0.222 | 0.016 | 0.003 | 8.60E-09 | 350470 | 0.008 | 28.444 |
| Platelet distribution width | rs16937003 | 10 | 80938499 | A | G | 0.02 | -0.074 | 0.009 | 4.78E-18 | 350470 | 0.019 | 67.605 |
| Platelet distribution width | rs2072114 | 11 | 61605215 | G | A | 0.123 | 0.022 | 0.004 | 8.72E-10 | 350470 | 0.009 | 30.250 |
| Platelet distribution width | rs964184 | 11 | 116648917 | C | G | 0.868 | -0.081 | 0.003 | 3.16E-120 | 350470 | 0.208 | 728.996 |
| Platelet distribution width | rs7949566 | 11 | 126285301 | A | G | 0.422 | 0.023 | 0.002 | 3.32E-21 | 350470 | 0.038 | 132.249 |
| Platelet distribution width | rs72847156 | 11 | 3881053 | T | G | 0.315 | 0.015 | 0.003 | 1.06E-08 | 350470 | 0.007 | 25.000 |
| Platelet distribution width | rs1467509 | 11 | 33057317 | C | A | 0.381 | 0.02 | 0.002 | 8.63E-16 | 350470 | 0.029 | 99.999 |
| Platelet distribution width | rs2291813 | 11 | 85956478 | T | G | 0.036 | -0.046 | 0.006 | 4.85E-13 | 350470 | 0.017 | 58.777 |
| Platelet distribution width | rs415895 | 11 | 9769562 | G | C | 0.645 | 0.031 | 0.002 | 3.21E-36 | 350470 | 0.069 | 240.249 |
| Platelet distribution width | rs4529894 | 11 | 57199645 | G | C | 0.217 | -0.029 | 0.003 | 7.55E-24 | 350470 | 0.027 | 93.444 |
| Platelet distribution width | rs10466710 | 11 | 77350337 | G | A | 0.374 | -0.02 | 0.002 | 2.66E-16 | 350470 | 0.029 | 99.999 |
| Platelet distribution width | rs201048317 | 11 | 223854 | T | C | 0.407 | 0.016 | 0.003 | 2.99E-10 | 350470 | 0.008 | 28.444 |
| Platelet distribution width | rs1961319 | 11 | 108293879 | T | G | 0.153 | -0.018 | 0.003 | 3.04E-08 | 350470 | 0.010 | 36.000 |
| Platelet distribution width | rs11235689 | 11 | 72949747 | T | C | 0.414 | 0.032 | 0.002 | 8.85E-40 | 350470 | 0.073 | 255.999 |
| Platelet distribution width | rs11224303 | 11 | 100456729 | A | G | 0.097 | 0.032 | 0.004 | 1.29E-15 | 350470 | 0.018 | 64.000 |
| Platelet distribution width | rs55970551 | 11 | 72150611 | T | C | 0.021 | -0.063 | 0.008 | 1.62E-14 | 350470 | 0.018 | 62.015 |
| Platelet distribution width | rs11604281 | 11 | 86082321 | C | A | 0.06 | -0.035 | 0.005 | 6.75E-12 | 350470 | 0.014 | 49.000 |
| Platelet distribution width | rs11553699 | 12 | 122216910 | G | A | 0.135 | 0.136 | 0.004 | 1.00E-200 | 350470 | 0.329 | 1155.993 |
| Platelet distribution width | rs10774122 | 12 | 3239298 | C | T | 0.604 | -0.041 | 0.002 | 2.44E-63 | 350470 | 0.120 | 420.248 |
| Platelet distribution width | rs11168246 | 12 | 48204627 | A | C | 0.21 | 0.017 | 0.003 | 3.20E-08 | 350470 | 0.009 | 32.111 |
| Platelet distribution width | rs10849411 | 12 | 6285818 | C | T | 0.43 | 0.021 | 0.002 | 4.78E-18 | 350470 | 0.031 | 110.249 |
| Platelet distribution width | rs61953500 | 12 | 121929055 | A | G | 0.073 | 0.029 | 0.005 | 5.38E-10 | 350470 | 0.010 | 33.640 |
| Platelet distribution width | rs1020848 | 12 | 57063459 | C | G | 0.66 | 0.066 | 0.002 | 2.22E-152 | 350470 | 0.310 | 1088.994 |
| Platelet distribution width | rs3742003 | 12 | 111844218 | A | G | 0.21 | -0.031 | 0.003 | 1.47E-26 | 350470 | 0.030 | 106.777 |
| Platelet distribution width | rs4760341 | 12 | 58281495 | A | T | 0.288 | -0.021 | 0.003 | 3.79E-15 | 350470 | 0.014 | 49.000 |
| Platelet distribution width | rs34038797 | 12 | 740009 | G | C | 0.48 | 0.031 | 0.002 | 5.15E-38 | 350470 | 0.069 | 240.249 |
| Platelet distribution width | rs10505718 | 12 | 791210 | G | C | 0.122 | -0.026 | 0.004 | 2.00E-12 | 350470 | 0.012 | 42.250 |
| Platelet distribution width | rs7958679 | 12 | 49662391 | T | C | 0.074 | 0.157 | 0.005 | 1.00E-200 | 350470 | 0.281 | 985.954 |
| Platelet distribution width | rs4759076 | 12 | 54729872 | C | T | 0.458 | -0.034 | 0.002 | 1.45E-45 | 350470 | 0.082 | 288.998 |
| Platelet distribution width | rs1006409 | 12 | 29435675 | G | A | 0.46 | 0.033 | 0.002 | 1.63E-44 | 350470 | 0.078 | 272.248 |
| Platelet distribution width | rs114282886 | 12 | 101883238 | T | C | 0.036 | 0.057 | 0.006 | 2.63E-19 | 350470 | 0.026 | 90.249 |
| Platelet distribution width | rs7952928 | 12 | 32828959 | A | G | 0.15 | -0.027 | 0.003 | 4.43E-16 | 350470 | 0.023 | 81.000 |
| Platelet distribution width | rs2288367 | 12 | 51690751 | T | G | 0.106 | -0.029 | 0.004 | 8.56E-14 | 350470 | 0.015 | 52.562 |
| Platelet distribution width | rs76582570 | 12 | 70517549 | G | C | 0.139 | 0.022 | 0.003 | 1.45E-10 | 350470 | 0.015 | 53.777 |
| Platelet distribution width | rs7978070 | 12 | 14511806 | C | T | 0.485 | -0.013 | 0.002 | 3.24E-08 | 350470 | 0.012 | 42.250 |
| Platelet distribution width | rs3861723 | 13 | 113910926 | G | A | 0.241 | -0.019 | 0.003 | 1.10E-11 | 350470 | 0.011 | 40.111 |
| Platelet distribution width | rs9534458 | 13 | 47237952 | A | G | 0.652 | 0.061 | 0.002 | 3.37E-132 | 350470 | 0.265 | 930.245 |
| Platelet distribution width | rs9549753 | 13 | 114018038 | A | C | 0.532 | 0.045 | 0.002 | 1.53E-79 | 350470 | 0.144 | 506.247 |
| Platelet distribution width | rs718444 | 13 | 33147052 | T | C | 0.371 | -0.019 | 0.002 | 5.41E-15 | 350470 | 0.026 | 90.249 |
| Platelet distribution width | rs1199955 | 13 | 37648464 | G | A | 0.895 | -0.034 | 0.004 | 2.11E-18 | 350470 | 0.021 | 72.250 |
| Platelet distribution width | rs7140110 | 13 | 114544024 | C | T | 0.297 | 0.017 | 0.003 | 1.50E-10 | 350470 | 0.009 | 32.111 |
| Platelet distribution width | rs58455864 | 13 | 110929605 | C | T | 0.197 | 0.022 | 0.003 | 8.35E-14 | 350470 | 0.015 | 53.777 |
| Platelet distribution width | rs2761843 | 13 | 51293037 | C | T | 0.157 | -0.019 | 0.003 | 2.33E-09 | 350470 | 0.011 | 40.111 |
| Platelet distribution width | rs754388 | 14 | 93115410 | C | G | 0.811 | -0.026 | 0.003 | 1.44E-17 | 350470 | 0.021 | 75.111 |
| Platelet distribution width | rs10142364 | 14 | 69489515 | G | A | 0.109 | -0.026 | 0.004 | 1.84E-11 | 350470 | 0.012 | 42.250 |
| Platelet distribution width | rs10142309 | 14 | 53507634 | G | C | 0.831 | 0.018 | 0.003 | 1.30E-08 | 350470 | 0.010 | 36.000 |
| Platelet distribution width | rs61977314 | 14 | 92934379 | G | A | 0.1 | 0.031 | 0.004 | 1.52E-15 | 350470 | 0.017 | 60.062 |
| Platelet distribution width | rs17694832 | 14 | 39860026 | A | G | 0.404 | 0.015 | 0.002 | 2.16E-10 | 350470 | 0.016 | 56.250 |
| Platelet distribution width | rs4906322 | 14 | 103879032 | G | A | 0.285 | -0.021 | 0.003 | 4.79E-16 | 350470 | 0.014 | 49.000 |
| Platelet distribution width | rs61269268 | 14 | 92241352 | C | A | 0.174 | 0.03 | 0.003 | 5.36E-22 | 350470 | 0.029 | 99.999 |
| Platelet distribution width | rs229576 | 14 | 65222190 | T | A | 0.176 | -0.022 | 0.003 | 2.07E-12 | 350470 | 0.015 | 53.777 |
| Platelet distribution width | rs2934691 | 14 | 51121333 | A | C | 0.563 | -0.014 | 0.002 | 2.16E-09 | 350470 | 0.014 | 49.000 |
| Platelet distribution width | rs7170637 | 15 | 22969232 | A | G | 0.16 | 0.028 | 0.003 | 9.35E-19 | 350470 | 0.025 | 87.111 |
| Platelet distribution width | rs4966015 | 15 | 99248132 | T | G | 0.144 | 0.03 | 0.003 | 1.48E-18 | 350470 | 0.029 | 99.999 |
| Platelet distribution width | rs12324667 | 15 | 64161785 | T | A | 0.19 | 0.02 | 0.003 | 3.06E-11 | 350470 | 0.013 | 44.444 |
| Platelet distribution width | rs1719285 | 15 | 65182223 | A | T | 0.157 | -0.085 | 0.003 | 1.07E-152 | 350470 | 0.229 | 802.773 |
| Platelet distribution width | rs66793829 | 15 | 63341238 | C | G | 0.168 | 0.053 | 0.003 | 1.51E-61 | 350470 | 0.089 | 312.109 |
| Platelet distribution width | rs12594925 | 15 | 91520287 | A | G | 0.128 | 0.022 | 0.004 | 3.02E-10 | 350470 | 0.009 | 30.250 |
| Platelet distribution width | rs12909047 | 15 | 74782356 | A | G | 0.505 | -0.023 | 0.002 | 4.01E-22 | 350470 | 0.038 | 132.249 |
| Platelet distribution width | rs4924406 | 15 | 40309704 | T | A | 0.56 | -0.019 | 0.002 | 7.08E-15 | 350470 | 0.026 | 90.249 |
| Platelet distribution width | rs117159862 | 15 | 40883919 | A | C | 0.033 | 0.042 | 0.007 | 4.06E-10 | 350470 | 0.010 | 36.000 |
| Platelet distribution width | rs62022924 | 15 | 86235507 | G | A | 0.237 | 0.016 | 0.003 | 1.54E-08 | 350470 | 0.008 | 28.444 |
| Platelet distribution width | rs4843185 | 16 | 85690338 | A | G | 0.271 | -0.04 | 0.003 | 8.82E-50 | 350470 | 0.051 | 177.777 |
| Platelet distribution width | rs9926137 | 16 | 9045462 | C | G | 0.189 | 0.038 | 0.003 | 5.92E-36 | 350470 | 0.046 | 160.444 |
| Platelet distribution width | rs1552272 | 16 | 85452695 | T | C | 0.648 | 0.028 | 0.002 | 1.51E-29 | 350470 | 0.056 | 195.999 |
| Platelet distribution width | rs10852622 | 16 | 88556880 | G | A | 0.284 | -0.02 | 0.003 | 4.24E-14 | 350470 | 0.013 | 44.444 |
| Platelet distribution width | rs12924211 | 16 | 79354047 | C | T | 0.667 | -0.017 | 0.003 | 7.79E-12 | 350470 | 0.009 | 32.111 |
| Platelet distribution width | rs72772025 | 16 | 15834729 | T | C | 0.258 | -0.015 | 0.003 | 3.54E-08 | 350470 | 0.007 | 25.000 |
| Platelet distribution width | rs9937661 | 16 | 5020514 | C | T | 0.465 | 0.029 | 0.002 | 8.92E-34 | 350470 | 0.060 | 210.249 |
| Platelet distribution width | rs12931931 | 16 | 531892 | A | C | 0.48 | 0.016 | 0.002 | 5.79E-11 | 350470 | 0.018 | 64.000 |
| Platelet distribution width | rs34480360 | 16 | 30798689 | A | G | 0.262 | 0.017 | 0.003 | 8.50E-10 | 350470 | 0.009 | 32.111 |
| Platelet distribution width | rs4783186 | 16 | 85415734 | C | T | 0.876 | 0.057 | 0.004 | 3.44E-57 | 350470 | 0.058 | 203.061 |
| Platelet distribution width | rs2377058 | 16 | 89734831 | G | A | 0.364 | 0.014 | 0.002 | 4.38E-09 | 350470 | 0.014 | 49.000 |
| Platelet distribution width | rs12938273 | 17 | 57901652 | A | G | 0.556 | -0.048 | 0.002 | 9.62E-90 | 350470 | 0.164 | 575.997 |
| Platelet distribution width | rs850735 | 17 | 42448180 | G | A | 0.489 | 0.027 | 0.002 | 5.26E-29 | 350470 | 0.052 | 182.249 |
| Platelet distribution width | rs666404 | 17 | 80772257 | G | A | 0.53 | 0.021 | 0.002 | 1.79E-19 | 350470 | 0.031 | 110.249 |
| Platelet distribution width | rs72829457 | 17 | 7568327 | T | C | 0.118 | -0.028 | 0.004 | 2.31E-14 | 350470 | 0.014 | 49.000 |
| Platelet distribution width | rs7216823 | 17 | 8210590 | G | A | 0.157 | -0.024 | 0.003 | 1.02E-13 | 350470 | 0.018 | 64.000 |
| Platelet distribution width | rs1436138 | 17 | 75316880 | G | A | 0.359 | 0.016 | 0.002 | 2.80E-11 | 350470 | 0.018 | 64.000 |
| Platelet distribution width | rs9900637 | 17 | 3951975 | A | C | 0.498 | 0.015 | 0.002 | 1.17E-10 | 350470 | 0.016 | 56.250 |
| Platelet distribution width | rs12936897 | 17 | 57424645 | A | G | 0.391 | -0.014 | 0.002 | 4.73E-09 | 350470 | 0.014 | 49.000 |
| Platelet distribution width | rs312824 | 17 | 75371267 | T | C | 0.702 | 0.014 | 0.003 | 2.93E-08 | 350470 | 0.006 | 21.778 |
| Platelet distribution width | rs183583130 | 17 | 4942516 | T | G | 0.024 | 0.074 | 0.008 | 1.91E-20 | 350470 | 0.024 | 85.562 |
| Platelet distribution width | rs17550268 | 17 | 33874678 | C | T | 0.222 | -0.025 | 0.003 | 1.12E-18 | 350470 | 0.020 | 69.444 |
| Platelet distribution width | rs12952555 | 17 | 78030604 | T | C | 0.437 | 0.015 | 0.002 | 1.01E-09 | 350470 | 0.016 | 56.250 |
| Platelet distribution width | rs1060431 | 17 | 4840868 | A | G | 0.073 | -0.088 | 0.005 | 1.02E-82 | 350470 | 0.088 | 309.758 |
| Platelet distribution width | rs847680 | 17 | 48224075 | C | T | 0.172 | 0.025 | 0.003 | 6.51E-16 | 350470 | 0.020 | 69.444 |
| Platelet distribution width | rs9914927 | 17 | 74409123 | T | C | 0.203 | 0.021 | 0.003 | 4.62E-13 | 350470 | 0.014 | 49.000 |
| Platelet distribution width | rs141336258 | 17 | 4824846 | C | T | 0.012 | 0.078 | 0.011 | 3.66E-12 | 350470 | 0.014 | 50.281 |
| Platelet distribution width | rs7503168 | 17 | 33885904 | G | A | 0.172 | -0.104 | 0.003 | 1.00E-200 | 350470 | 0.342 | 1201.771 |
| Platelet distribution width | rs28883140 | 17 | 10183555 | A | G | 0.223 | -0.028 | 0.003 | 1.24E-22 | 350470 | 0.025 | 87.111 |
| Platelet distribution width | rs671339 | 18 | 9618005 | G | A | 0.737 | -0.039 | 0.003 | 8.23E-48 | 350470 | 0.048 | 168.999 |
| Platelet distribution width | rs8098454 | 18 | 22463497 | T | C | 0.087 | -0.027 | 0.004 | 5.69E-11 | 350470 | 0.013 | 45.562 |
| Platelet distribution width | rs11082304 | 18 | 20720973 | T | G | 0.514 | 0.043 | 0.002 | 3.06E-74 | 350470 | 0.132 | 462.247 |
| Platelet distribution width | rs718515 | 18 | 43856297 | A | G | 0.552 | 0.017 | 0.002 | 3.14E-12 | 350470 | 0.021 | 72.250 |
| Platelet distribution width | rs892090 | 19 | 55539072 | G | T | 0.835 | 0.081 | 0.003 | 1.66E-142 | 350470 | 0.208 | 728.996 |
| Platelet distribution width | rs57843631 | 19 | 16206012 | T | C | 0.019 | 0.226 | 0.009 | 2.91E-141 | 350470 | 0.180 | 630.564 |
| Platelet distribution width | rs3934667 | 19 | 19431423 | G | T | 0.681 | 0.02 | 0.003 | 2.89E-14 | 350470 | 0.013 | 44.444 |
| Platelet distribution width | rs77808283 | 19 | 16189882 | A | G | 0.039 | -0.034 | 0.006 | 2.51E-08 | 350470 | 0.009 | 32.111 |
| Platelet distribution width | rs61166126 | 19 | 17252974 | C | T | 0.311 | -0.021 | 0.003 | 9.65E-16 | 350470 | 0.014 | 49.000 |
| Platelet distribution width | rs2014850 | 19 | 35662505 | A | G | 0.716 | 0.033 | 0.003 | 1.47E-34 | 350470 | 0.035 | 120.999 |
| Platelet distribution width | rs883394 | 19 | 39205613 | T | C | 0.485 | 0.018 | 0.002 | 5.24E-14 | 350470 | 0.023 | 81.000 |
| Platelet distribution width | rs56681946 | 19 | 44283031 | C | T | 0.377 | 0.015 | 0.002 | 2.35E-09 | 350470 | 0.016 | 56.250 |
| Platelet distribution width | rs8106212 | 19 | 6802571 | T | C | 0.012 | 0.148 | 0.011 | 3.48E-41 | 350470 | 0.052 | 181.024 |
| Platelet distribution width | rs10404811 | 19 | 58154807 | C | T | 0.146 | 0.03 | 0.003 | 8.82E-19 | 350470 | 0.029 | 99.999 |
| Platelet distribution width | rs35564315 | 19 | 2007315 | A | C | 0.166 | -0.026 | 0.003 | 8.98E-16 | 350470 | 0.021 | 75.111 |
| Platelet distribution width | rs181167720 | 19 | 58916761 | G | A | 0.23 | -0.016 | 0.003 | 2.34E-08 | 350470 | 0.008 | 28.444 |
| Platelet distribution width | rs11083767 | 19 | 45715680 | C | T | 0.339 | 0.05 | 0.002 | 7.87E-90 | 350470 | 0.178 | 624.996 |
| Platelet distribution width | rs11671059 | 19 | 55698183 | C | T | 0.091 | 0.035 | 0.004 | 4.60E-17 | 350470 | 0.022 | 76.562 |
| Platelet distribution width | rs8113274 | 19 | 10666380 | A | T | 0.233 | -0.021 | 0.003 | 1.14E-13 | 350470 | 0.014 | 49.000 |
| Platelet distribution width | rs463312 | 20 | 57597970 | C | A | 0.047 | 0.521 | 0.005 | 1.00E-200 | 350470 | 3.005 | 10857.578 |
| Platelet distribution width | rs187115451 | 20 | 37406819 | A | T | 0.049 | 0.048 | 0.006 | 3.53E-15 | 350470 | 0.018 | 64.000 |
| Platelet distribution width | rs13039242 | 20 | 19905434 | G | A | 0.129 | -0.032 | 0.004 | 8.10E-19 | 350470 | 0.018 | 64.000 |
| Platelet distribution width | rs8117825 | 20 | 57832175 | C | A | 0.068 | 0.095 | 0.005 | 7.22E-92 | 350470 | 0.103 | 360.998 |
| Platelet distribution width | rs6086540 | 20 | 8603950 | C | G | 0.507 | 0.036 | 0.002 | 1.37E-52 | 350470 | 0.092 | 323.998 |
| Platelet distribution width | rs11696739 | 20 | 1600925 | A | G | 0.378 | -0.026 | 0.002 | 3.85E-26 | 350470 | 0.048 | 168.999 |
| Platelet distribution width | rs200891 | 20 | 1791920 | G | T | 0.607 | -0.019 | 0.002 | 3.11E-14 | 350470 | 0.026 | 90.249 |
| Platelet distribution width | rs139619205 | 20 | 19982174 | G | A | 0.005 | 0.12 | 0.017 | 3.36E-12 | 350470 | 0.014 | 49.827 |
| Platelet distribution width | rs151332 | 20 | 57552121 | G | A | 0.93 | 0.089 | 0.005 | 2.16E-82 | 350470 | 0.090 | 316.838 |
| Platelet distribution width | rs3761133 | 20 | 57210480 | C | G | 0.68 | -0.032 | 0.003 | 6.44E-35 | 350470 | 0.032 | 113.777 |
| Platelet distribution width | rs6124259 | 20 | 39270795 | A | G | 0.619 | 0.017 | 0.002 | 2.75E-12 | 350470 | 0.021 | 72.250 |
| Platelet distribution width | rs6012519 | 20 | 47326000 | A | G | 0.2 | -0.017 | 0.003 | 3.57E-09 | 350470 | 0.009 | 32.111 |
| Platelet distribution width | rs116274237 | 21 | 46910352 | G | C | 0.256 | -0.019 | 0.003 | 1.09E-11 | 350470 | 0.011 | 40.111 |
| Platelet distribution width | rs9636612 | 21 | 16396991 | G | A | 0.373 | 0.026 | 0.002 | 2.31E-26 | 350470 | 0.048 | 168.999 |
| Platelet distribution width | rs1137600 | 21 | 38823260 | G | A | 0.289 | -0.014 | 0.003 | 2.96E-08 | 350470 | 0.006 | 21.778 |
| Platelet distribution width | rs5758910 | 22 | 43145853 | C | G | 0.676 | -0.027 | 0.003 | 3.70E-27 | 350470 | 0.023 | 81.000 |
| Platelet distribution width | rs134887 | 22 | 42674239 | G | A | 0.661 | 0.03 | 0.002 | 8.54E-33 | 350470 | 0.064 | 224.999 |
| Platelet distribution width | rs2097598 | 22 | 19689697 | G | C | 0.377 | 0.026 | 0.003 | 1.09E-25 | 350470 | 0.021 | 75.111 |
| Platelet distribution width | rs34834842 | 22 | 38328597 | G | A | 0.038 | 0.103 | 0.006 | 6.42E-62 | 350470 | 0.084 | 294.693 |
| Platelet distribution width | rs192565874 | 22 | 21042969 | A | G | 0.082 | -0.025 | 0.004 | 1.22E-08 | 350470 | 0.011 | 39.062 |
| Platelet distribution width | rs459310 | 22 | 29819081 | A | G | 0.45 | 0.013 | 0.002 | 3.24E-08 | 350470 | 0.012 | 42.250 |
| Platelet distribution width | rs4822250 | 22 | 43409058 | A | C | 0.551 | 0.096 | 0.002 | 1.00E-200 | 350470 | 0.653 | 2303.987 |
| Platelet distribution width | rs1034564 | 22 | 19984013 | T | C | 0.286 | -0.021 | 0.003 | 2.51E-16 | 350470 | 0.014 | 49.000 |
| Platelet distribution width | rs6000553 | 22 | 37469192 | G | A | 0.533 | 0.019 | 0.002 | 1.54E-15 | 350470 | 0.026 | 90.249 |
| RA | rs1071649 | 6 | 31239114 | T | G | 0.017 | 0.315 | 0.020 | 1.90E-57 | 58284 | 0.436 | 255.202 |
| RA | rs10790268 | 11 | 118729391 | G | A | 0.189 | 0.163 | 0.021 | 3.30E-15 | 58284 | 0.106 | 62.077 |
| RA | rs10796038 | 10 | 6397964 | G | A | 0.176 | 0.117 | 0.019 | 2.00E-09 | 58284 | 0.062 | 35.973 |
| RA | rs10858006 | 1 | 114114965 | G | A | 0.201 | 0.105 | 0.018 | 7.10E-09 | 58284 | 0.057 | 33.506 |
| RA | rs10985070 | 9 | 123636121 | A | C | 0.434 | -0.083 | 0.015 | 1.70E-08 | 58284 | 0.055 | 31.809 |
| RA | rs11203203 | 21 | 43836186 | A | G | 0.352 | 0.086 | 0.016 | 4.70E-08 | 58284 | 0.051 | 29.836 |
| RA | rs11574914 | 9 | 34710338 | A | G | 0.315 | 0.122 | 0.017 | 1.50E-13 | 58284 | 0.094 | 54.569 |
| RA | rs11968393 | 6 | 32779073 | G | T | 0.011 | 0.342 | 0.048 | 6.40E-13 | 58284 | 0.089 | 51.718 |
| RA | rs12232497 | 17 | 38040119 | C | T | 0.472 | 0.094 | 0.016 | 3.60E-09 | 58284 | 0.060 | 34.828 |
| RA | rs12539741 | 7 | 128596805 | T | C | 0.100 | 0.157 | 0.023 | 1.20E-11 | 58284 | 0.079 | 45.970 |
| RA | rs12764378 | 10 | 63800004 | A | G | 0.245 | 0.131 | 0.018 | 1.90E-13 | 58284 | 0.093 | 54.103 |
| RA | rs13330176 | 16 | 86019087 | A | T | 0.240 | 0.113 | 0.020 | 9.00E-09 | 58284 | 0.057 | 33.045 |
| RA | rs13426947 | 2 | 191933254 | A | G | 0.191 | 0.131 | 0.019 | 2.40E-12 | 58284 | 0.084 | 49.125 |
| RA | rs1571878 | 6 | 167540842 | T | C | 0.422 | -0.117 | 0.015 | 4.90E-15 | 58284 | 0.105 | 61.299 |
| RA | rs17208363 | 6 | 32297209 | A | G | 0.070 | -0.580 | 0.038 | 2.50E-51 | 58284 | 0.388 | 227.137 |
| RA | rs17264332 | 6 | 138005515 | G | A | 0.169 | 0.163 | 0.018 | 7.10E-19 | 58284 | 0.135 | 78.733 |
| RA | rs1830873 | 6 | 33620397 | G | C | 0.454 | 0.199 | 0.016 | 4.60E-37 | 58284 | 0.277 | 161.783 |
| RA | rs1858037 | 2 | 65598300 | A | T | 0.339 | -0.105 | 0.017 | 5.90E-10 | 58284 | 0.066 | 38.353 |
| RA | rs2069235 | 22 | 39747780 | A | G | 0.299 | 0.104 | 0.017 | 3.00E-10 | 58284 | 0.068 | 39.673 |
| RA | rs2229527 | 6 | 32797309 | G | T | 0.065 | 0.580 | 0.029 | 2.30E-90 | 58284 | 0.692 | 406.322 |
| RA | rs2233434 | 6 | 44232920 | G | A | 0.050 | 0.288 | 0.052 | 3.30E-08 | 58284 | 0.052 | 30.522 |
| RA | rs2240336 | 1 | 17674402 | T | C | 0.415 | -0.105 | 0.017 | 1.40E-09 | 58284 | 0.063 | 36.668 |
| RA | rs225433 | 21 | 43809418 | G | C | 0.189 | -0.128 | 0.023 | 1.80E-08 | 58284 | 0.054 | 31.698 |
| RA | rs2451258 | 6 | 159506600 | T | C | 0.348 | 0.104 | 0.017 | 6.60E-10 | 58284 | 0.065 | 38.134 |
| RA | rs2476601 | 1 | 114377568 | G | A | 0.094 | -0.593 | 0.023 | 1.60E-149 | 58284 | 1.150 | 678.232 |
| RA | rs2561477 | 5 | 102608924 | A | G | 0.302 | -0.105 | 0.017 | 5.20E-10 | 58284 | 0.066 | 38.600 |
| RA | rs28411352 | 1 | 38278579 | T | C | 0.251 | 0.104 | 0.018 | 5.20E-09 | 58284 | 0.058 | 34.112 |
| RA | rs2844456 | 6 | 31864674 | C | T | 0.040 | 0.892 | 0.029 | 1.00E-200 | 58284 | 1.575 | 932.873 |
| RA | rs3087243 | 2 | 204738919 | A | G | 0.470 | -0.139 | 0.015 | 9.20E-20 | 58284 | 0.142 | 82.771 |
| RA | rs3094633 | 6 | 30215120 | T | C | 0.463 | 0.191 | 0.016 | 2.60E-34 | 58284 | 0.255 | 149.190 |
| RA | rs3129967 | 6 | 32381461 | T | C | 0.296 | -0.635 | 0.019 | 1.00E-200 | 58284 | 1.925 | 1143.761 |
| RA | rs3134976 | 6 | 32652305 | C | A | 0.222 | 0.799 | 0.024 | 1.00E-200 | 58284 | 1.925 | 1143.756 |
| RA | rs34046593 | 4 | 26111593 | A | G | 0.321 | 0.140 | 0.017 | 9.20E-17 | 58284 | 0.118 | 69.131 |
| RA | rs34536443 | 19 | 10463118 | C | G | 0.029 | -0.378 | 0.047 | 4.60E-16 | 58284 | 0.113 | 65.958 |
| RA | rs34673422 | 6 | 32332661 | C | A | 0.018 | 0.777 | 0.056 | 2.70E-44 | 58284 | 0.333 | 194.900 |
| RA | rs34695944 | 2 | 61124850 | C | T | 0.361 | 0.117 | 0.015 | 4.40E-14 | 58284 | 0.098 | 56.980 |
| RA | rs34767257 | 6 | 30932606 | A | G | 0.075 | 0.329 | 0.027 | 2.00E-33 | 58284 | 0.248 | 145.136 |
| RA | rs3749946 | 6 | 31448862 | A | C | 0.073 | -0.329 | 0.033 | 4.00E-23 | 58284 | 0.168 | 98.085 |
| RA | rs3873445 | 6 | 32683010 | T | C | 0.116 | 0.765 | 0.023 | 1.00E-200 | 58284 | 1.815 | 1077.293 |
| RA | rs4239702 | 20 | 44749251 | C | T | 0.287 | 0.139 | 0.018 | 4.20E-14 | 58284 | 0.098 | 57.070 |
| RA | rs4452313 | 3 | 17047032 | T | A | 0.308 | 0.105 | 0.017 | 2.70E-10 | 58284 | 0.068 | 39.880 |
| RA | rs4711325 | 6 | 33432709 | G | A | 0.324 | -0.131 | 0.017 | 1.10E-14 | 58284 | 0.102 | 59.706 |
| RA | rs4713424 | 6 | 31002742 | A | G | 0.089 | -0.288 | 0.034 | 4.70E-17 | 58284 | 0.121 | 70.456 |
| RA | rs4936059 | 11 | 128502496 | G | A | 0.340 | 0.094 | 0.017 | 4.60E-08 | 58284 | 0.051 | 29.877 |
| RA | rs511027 | 6 | 32206687 | T | A | 0.114 | -0.673 | 0.030 | 1.80E-109 | 58284 | 0.841 | 494.113 |
| RA | rs537544 | 10 | 8108382 | T | C | 0.380 | -0.117 | 0.018 | 8.00E-11 | 58284 | 0.072 | 42.257 |
| RA | rs592390 | 18 | 12822314 | C | T | 0.477 | -0.095 | 0.016 | 3.80E-09 | 58284 | 0.060 | 34.722 |
| RA | rs60733400 | 1 | 2516781 | A | G | 0.314 | -0.105 | 0.017 | 1.30E-09 | 58284 | 0.063 | 36.812 |
| RA | rs61828284 | 1 | 173299743 | T | C | 0.080 | -0.198 | 0.034 | 8.70E-09 | 58284 | 0.057 | 33.111 |
| RA | rs624988 | 1 | 117263790 | C | T | 0.412 | -0.086 | 0.016 | 4.60E-08 | 58284 | 0.051 | 29.877 |
| RA | rs628357 | 6 | 31806598 | A | G | 0.033 | -0.598 | 0.070 | 1.20E-17 | 58284 | 0.125 | 73.150 |
| RA | rs6450 | 6 | 32006797 | A | G | 0.025 | 0.270 | 0.049 | 3.10E-08 | 58284 | 0.053 | 30.643 |
| RA | rs6913550 | 6 | 26540683 | C | T | 0.240 | -0.104 | 0.019 | 2.50E-08 | 58284 | 0.053 | 31.060 |
| RA | rs706778 | 10 | 6098949 | T | C | 0.414 | 0.104 | 0.015 | 7.10E-12 | 58284 | 0.081 | 46.998 |
| RA | rs72495971 | 6 | 29861111 | G | A | 0.029 | 0.301 | 0.043 | 2.10E-12 | 58284 | 0.085 | 49.387 |
| RA | rs73081554 | 3 | 58302935 | T | C | 0.052 | 0.166 | 0.030 | 4.70E-08 | 58284 | 0.051 | 29.836 |
| RA | rs73409695 | 6 | 32964256 | C | T | 0.050 | -0.329 | 0.045 | 3.50E-13 | 58284 | 0.091 | 52.904 |
| RA | rs7731626 | 5 | 55444683 | A | G | 0.378 | -0.198 | 0.020 | 7.90E-23 | 58284 | 0.166 | 96.738 |
| RA | rs8026898 | 15 | 69991417 | A | G | 0.285 | 0.148 | 0.018 | 2.40E-17 | 58284 | 0.123 | 71.782 |
| RA | rs8032939 | 15 | 38834033 | C | T | 0.242 | 0.117 | 0.017 | 2.40E-12 | 58284 | 0.084 | 49.125 |
| RA | rs8133843 | 21 | 36738242 | A | G | 0.375 | 0.095 | 0.016 | 6.00E-09 | 58284 | 0.058 | 33.834 |
| RA | rs8192585 | 6 | 32188823 | A | G | 0.038 | 0.507 | 0.033 | 3.50E-52 | 58284 | 0.395 | 231.053 |
| RA | rs9258658 | 6 | 29822647 | T | C | 0.207 | -0.174 | 0.022 | 7.50E-16 | 58284 | 0.111 | 64.995 |
| RA | rs9261602 | 2 | 76934183 | G | A | 0.452 | 0.186 | 0.022 | 4.50E-18 | 58284 | 0.129 | 75.086 |
| RA | rs9267059 | 6 | 31397416 | T | C | 0.048 | 0.329 | 0.047 | 2.50E-12 | 58284 | 0.084 | 49.045 |
| RA | rs9277550 | 6 | 33055487 | C | T | 0.316 | -0.365 | 0.018 | 4.60E-90 | 58284 | 0.690 | 404.939 |
| RA | rs9310852 | 3 | 27784997 | G | A | 0.462 | 0.083 | 0.015 | 3.20E-08 | 58284 | 0.052 | 30.581 |
| RA | rs9468500 | 6 | 29210998 | T | A | 0.049 | -0.191 | 0.035 | 3.40E-08 | 58284 | 0.052 | 30.464 |
| RA | rs9603608 | 13 | 40318819 | C | A | 0.344 | -0.104 | 0.016 | 7.60E-11 | 58284 | 0.073 | 42.357 |
| RA | rs9653442 | 2 | 100825367 | T | C | 0.453 | -0.105 | 0.015 | 3.60E-12 | 58284 | 0.083 | 48.330 |
| Other RA (FinnGen) | rs2476601 | 1 | 114377568 | G | A | 0.852 | -0.494 | 0.049 | 2.21E-24 | 217314 | 0.048 | 103.745 |
| Other RA (FinnGen) | rs9368744 | 6 | 32798299 | T | C | 0.028 | 0.585 | 0.105 | 2.52E-08 | 217314 | 0.014 | 31.041 |
| Other RA (FinnGen) | rs9277419 | 6 | 33051777 | G | A | 0.250 | -0.278 | 0.041 | 8.37E-12 | 217314 | 0.022 | 46.750 |
| Other RA (FinnGen) | rs114799031 | 6 | 28153282 | T | A | 0.063 | 0.447 | 0.070 | 1.77E-10 | 217314 | 0.019 | 40.734 |
| Other RA (FinnGen) | rs114291795 | 6 | 31377640 | G | C | 0.077 | 0.918 | 0.068 | 8.21E-42 | 217314 | 0.084 | 183.405 |
| Other RA (FinnGen) | rs144112342 | 6 | 32858077 | C | T | 0.018 | 1.231 | 0.139 | 6.32E-19 | 217314 | 0.036 | 78.909 |
| Other RA (FinnGen) | rs9391630 | 6 | 29722515 | G | A | 0.288 | 0.333 | 0.038 | 1.60E-18 | 217314 | 0.035 | 76.966 |
| Other RA (FinnGen) | rs62402701 | 6 | 32351217 | T | A | 0.062 | -0.421 | 0.073 | 7.57E-09 | 217314 | 0.015 | 33.398 |
| Other RA (FinnGen) | rs115469976 | 6 | 32986508 | C | T | 0.046 | 0.666 | 0.087 | 2.26E-14 | 217314 | 0.027 | 58.252 |
| Other RA (FinnGen) | rs74376220 | 6 | 134942328 | A | G | 0.011 | 0.991 | 0.175 | 1.49E-08 | 217314 | 0.015 | 32.057 |
| Other RA (FinnGen) | rs34434863 | 6 | 32559673 | G | T | 0.338 | 0.809 | 0.047 | 2.05E-66 | 217314 | 0.136 | 295.910 |
| Other RA (FinnGen) | rs3131640 | 6 | 31472862 | T | A | 0.572 | 0.305 | 0.037 | 9.83E-17 | 217314 | 0.032 | 68.885 |
| Other RA (FinnGen) | rs9265581 | 6 | 31298901 | A | G | 0.746 | 0.284 | 0.040 | 8.11E-13 | 217314 | 0.024 | 51.324 |
| Other RA (FinnGen) | rs206763 | 6 | 32970197 | A | G | 0.016 | 1.165 | 0.147 | 2.25E-15 | 217314 | 0.029 | 62.840 |
| Other RA (FinnGen) | rs9274719 | 6 | 32637470 | C | T | 0.193 | 0.676 | 0.048 | 1.44E-44 | 217314 | 0.090 | 196.114 |
| Seronegetive RA (FinnGen) | rs2476601 | 1 | 114377568 | G | A | 0.853 | -0.321 | 0.047 | 9.96E-12 | 174841 | 0.026 | 46.309 |
| Seronegetive RA (FinnGen) | rs147392974 | 6 | 27299591 | A | G | 0.029 | 0.617 | 0.104 | 2.49E-09 | 174841 | 0.020 | 35.537 |
| Seronegetive RA (FinnGen) | rs2394437 | 6 | 30936534 | A | G | 0.236 | 0.229 | 0.040 | 1.09E-08 | 174841 | 0.019 | 32.697 |
| Seronegetive RA (FinnGen) | rs3104412 | 6 | 32585967 | G | A | 0.463 | 0.291 | 0.036 | 6.09E-16 | 174841 | 0.037 | 65.569 |
| Seronegetive RA (FinnGen) | rs34434863 | 6 | 32559673 | G | T | 0.336 | 0.513 | 0.046 | 9.73E-29 | 174841 | 0.071 | 123.686 |
| Seronegetive RA (FinnGen) | rs114799031 | 6 | 28153282 | T | A | 0.061 | 0.519 | 0.073 | 9.27E-13 | 174841 | 0.029 | 51.023 |
| Seronegetive RA (FinnGen) | rs144112342 | 6 | 32858077 | C | T | 0.018 | 0.971 | 0.136 | 1.11E-12 | 174841 | 0.029 | 50.624 |
| Seronegetive RA (FinnGen) | rs145575084 | 6 | 31365089 | C | G | 0.075 | 0.873 | 0.068 | 1.49E-37 | 174841 | 0.094 | 163.853 |
| Seronegetive RA (FinnGen) | rs1476573 | 6 | 29674648 | G | A | 0.610 | 0.278 | 0.037 | 3.58E-14 | 174841 | 0.033 | 57.379 |
| Seropositive RA (FinnGen) | rs56175143 | 1 | 114599755 | A | G | 0.049 | 0.339 | 0.052 | 6.51E-11 | 177430 | 0.024 | 42.613 |
| Seropositive RA (FinnGen) | rs2476601 | 1 | 114377568 | G | A | 0.852 | -0.465 | 0.032 | 5.48E-47 | 177430 | 0.117 | 207.429 |
| Seropositive RA (FinnGen) | rs11571293 | 2 | 204717713 | T | G | 0.316 | -0.155 | 0.024 | 1.61E-10 | 177430 | 0.023 | 41.023 |
| Seropositive RA (FinnGen) | rs7731626 | 5 | 55444683 | A | G | 0.280 | -0.140 | 0.025 | 2.46E-08 | 177430 | 0.017 | 30.977 |
| Seropositive RA (FinnGen) | rs144952788 | 6 | 26494079 | G | A | 0.038 | 0.495 | 0.061 | 3.26E-16 | 177430 | 0.038 | 66.586 |
| Seropositive RA (FinnGen) | rs2523572 | 6 | 31329494 | T | C | 0.601 | 0.356 | 0.026 | 6.25E-44 | 177430 | 0.109 | 193.273 |
| Seropositive RA (FinnGen) | rs34434863 | 6 | 32559673 | G | T | 0.340 | 0.968 | 0.032 | 1.00E-200 | 177430 | 0.530 | 945.113 |
| Seropositive RA (FinnGen) | rs10947417 | 6 | 33599759 | C | T | 0.284 | -0.171 | 0.025 | 1.09E-11 | 177430 | 0.026 | 46.196 |
| Seropositive RA (FinnGen) | rs9391682 | 6 | 30357291 | T | G | 0.365 | -0.154 | 0.025 | 1.08E-09 | 177430 | 0.021 | 37.248 |
| Seropositive RA (FinnGen) | rs3993757 | 6 | 31590746 | T | C | 0.024 | 0.971 | 0.078 | 7.82E-36 | 177430 | 0.088 | 156.168 |
| Seropositive RA (FinnGen) | rs188302747 | 6 | 32232976 | G | C | 0.015 | -0.664 | 0.103 | 1.23E-10 | 177430 | 0.023 | 41.453 |
| Seropositive RA (FinnGen) | rs1571878 | 6 | 167540842 | T | C | 0.553 | -0.136 | 0.023 | 2.24E-09 | 177430 | 0.020 | 35.631 |
| Seropositive RA (FinnGen) | rs2856822 | 6 | 33047432 | C | A | 0.255 | -0.289 | 0.027 | 5.84E-26 | 177430 | 0.063 | 111.324 |
| Seropositive RA (FinnGen) | rs17190120 | 6 | 31013487 | T | G | 0.033 | 0.717 | 0.067 | 1.18E-26 | 177430 | 0.064 | 114.243 |
| Seropositive RA (FinnGen) | rs9380146 | 6 | 29817244 | A | G | 0.269 | 0.222 | 0.026 | 4.56E-18 | 177430 | 0.042 | 74.930 |
| Seropositive RA (FinnGen) | rs449635 | 6 | 33024166 | G | A | 0.072 | 0.300 | 0.045 | 1.72E-11 | 177430 | 0.025 | 45.245 |
| Seropositive RA (FinnGen) | rs142113043 | 6 | 32546300 | T | C | 0.009 | 0.852 | 0.131 | 7.72E-11 | 177430 | 0.024 | 42.344 |
| Seropositive RA (FinnGen) | rs78749293 | 6 | 28371184 | C | T | 0.072 | 0.240 | 0.044 | 3.45E-08 | 177430 | 0.017 | 30.364 |
| Seropositive RA (FinnGen) | rs2857599 | 6 | 31562150 | A | G | 0.168 | 0.196 | 0.030 | 1.11E-10 | 177430 | 0.023 | 41.672 |
| Seropositive RA (FinnGen) | rs9276710 | 6 | 32757102 | C | T | 0.209 | 0.431 | 0.030 | 9.55E-46 | 177430 | 0.114 | 202.050 |
| Seropositive RA (FinnGen) | rs7757831 | 6 | 32345183 | G | A | 0.062 | -0.468 | 0.051 | 3.61E-20 | 177430 | 0.048 | 84.610 |
| Seropositive RA (FinnGen) | rs9368744 | 6 | 32798299 | T | C | 0.028 | 0.492 | 0.070 | 1.48E-12 | 177430 | 0.028 | 50.010 |
| Seropositive RA (FinnGen) | rs150420498 | 6 | 33457505 | A | G | 0.039 | 0.649 | 0.062 | 5.44E-26 | 177430 | 0.063 | 111.103 |
| Seropositive RA (FinnGen) | rs9264277 | 6 | 31224667 | C | T | 0.729 | 0.196 | 0.026 | 6.61E-14 | 177430 | 0.032 | 56.278 |
| Seropositive RA (FinnGen) | rs206763 | 6 | 32970197 | A | G | 0.015 | 1.163 | 0.097 | 7.29E-33 | 177430 | 0.080 | 142.573 |
| Seropositive RA (FinnGen) | rs113119046 | 6 | 33021130 | A | C | 0.062 | -0.354 | 0.050 | 1.86E-12 | 177430 | 0.028 | 49.642 |
| Seropositive RA (FinnGen) | rs539284657 | 6 | 32542167 | T | C | 0.094 | -0.283 | 0.040 | 1.05E-12 | 177430 | 0.029 | 50.671 |
| Seropositive RA (FinnGen) | rs9274719 | 6 | 32637470 | C | T | 0.194 | 0.757 | 0.033 | 1.69E-119 | 177430 | 0.303 | 538.633 |
| Seropositive RA (FinnGen) | rs7741521 | 6 | 31442748 | A | G | 0.049 | -0.302 | 0.054 | 1.78E-08 | 177430 | 0.018 | 31.711 |
| Seropositive RA (FinnGen) | rs3778754 | 7 | 128575552 | G | C | 0.430 | 0.126 | 0.023 | 2.47E-08 | 177430 | 0.018 | 31.181 |

RA: rheumatoid arthritis;

**Supplementary Table S2 Causal association between PCT and RA**

| Exposure | Outcome | SNP  (n) | IVW | | | Weighted Median | | Weighted Mode | | MR egger | | | | |
| --- | --- | --- | --- | --- | --- | --- | --- | --- | --- | --- | --- | --- | --- | --- |
|  |  |  | OR (95%CI) | *P* value | *P* heterogeneity | OR (95%CI) | *P* value | OR (95%CI) | *P* value | OR (95%CI) | *P* value | Intercept | se | *P* value |
| PCT | RA  (ukb) | 304 | 1.13 (1.01, 1.29) | **0.012** | 1.67E-71 | 1.21 (1.02, 1.43) | **0.008** | 1.60 (1.18, 2.17) | 0.001 | 0.97 (0.82, 1.14) | 0.361 | 0.001 | 0.003 | 0.641 |
| PCT | Other RA (FinnGen) | 387 | 1.07 (1.01, 1.22) | 0.025 | 1.90E-10 | 1.01 (0.83, 1.24) | 0.956 | 0.93 (0.68, 1.28) | 0.332 | 1.09 (0.86, 1.38) | 0.068 | -4.74E-03 | 4.54E-03 | 0.297 |
| PCT | Seropositive RA  (FinnGen) | 387 | 1.03 (1.01, 1.21) | **0.003** | 3.98E-07 | 1.06 (1.01, 1.21) | **0.002** | 0.87 (0.55, 1.37) | 0.061 | 1.12 (1.05, 1.49) | 0.009 | -2.75E-03 | 4.35E-03 | 0.528 |
| PCT | Seronegative RA  (FinnGen) | 387 | 0.91 (0.83, 1.00) | 0.671 | 1.31E-16 | 0.89 (0.76, 1.04) | 0.649 | 0.91 (0.79, 1.04) | 0.837 | 0.91 (0.79, 1.05) | 0.283 | -3.81E-03 | 3.26E-03 | 0.243 |

**Supplementary Table S3 Causal association between PLT and RA**

| Exposure | Outcome | SNP  (n) | IVW | | | Weighted Median | | Weighted Mode | | MR egger | | | | |
| --- | --- | --- | --- | --- | --- | --- | --- | --- | --- | --- | --- | --- | --- | --- |
|  |  |  | OR (95%CI) | *P* value | *P* heterogeneity | OR (95%CI) | *P* value | OR (95%CI) | *P* value | OR (95%CI) | *P* value | Intercept | se | *P* value |
| PLT | RA  (ukb) | 356 | 0.90 (0.80, 1.02) | 0.320 | 1.81E-256 | 0.92 (0.75, 1.14) | 0.064 | 0.93 (0.78, 1.10) | 0.153 | 0.98 (0.82, 1.18) | 0.054 | -0.008 | 0.004 | 0.022 |
| PLT | Other RA  (FinnGen) | 416 | 1.14 (0.98, 1.32) | 0.084 | 4.38E-12 | 0.96 (0.77, 1.20) | 0.732 | 0.94 (0.71, 1.24) | 0.637 | 1.15 (0.88, 1.50) | 0.296 | -4.58E-04 | 4.35E-03 | 0.916 |
| PLT | Seropositive RA  (FinnGen) | 416 | 1.21 (1.02, 1.42) | **0.028** | 3.81E-04 | 1.01 (1.01, 1.28) | 0.032 | 1.01 (0.69, 1.47) | 0.962 | 1.38 (1.02, 1.87) | 0.037 | -6.02E-04 | 3.90E-03 | 0.878 |
| PLT | SeronegativeRA  (FinnGen) | 416 | 0.92 (0.84, 1.02) | 0.298 | 1.76E-25 | 0.95 (0.82, 1.09) | 0.884 | 0.99 (0.86, 1.14) | 0.866 | 0.95 (0.82, 1.11) | 0.531 | -2.38E-03 | 3.26E-03 | 0.467 |

**Supplementary Table S4 Causal association between PDW and RA**

| Exposure | Outcome | SNP  (n) | IVW | | | Weighted Median | | Weighted Mode | | MR egger | | | | |
| --- | --- | --- | --- | --- | --- | --- | --- | --- | --- | --- | --- | --- | --- | --- |
|  |  |  | OR (95%CI) | *P* value | *P* heterogeneity | OR (95%CI) | *P* value | OR (95%CI) | *P* value | OR (95%CI) | *P* value | Intercept | se | *P* value |
| PDW | RA  (ukb) | 270 | 1.01 (0.87, 1.17) | 0.909 | 1.69E-249 | 0.89 (0.79, 1.01) | 0.069 | 0.87 (0.76, 1.00) | 0.057 | 0.97 (0.73, 1.27) | 0.806 | 0.002 | 0.004 | 0.712 |
| PDW | Other RA (FinnGen) | 322 | 1.07 (0.94, 1.21) | 0.108 | 1.30E-03 | 1.14 (0.99, 1.30) | 0.468 | 1.13 (0.95, 1.35) | 0.214 | 1.35 (1.06, 1.71) | 0.121 | -4.52E-03 | 3.75E-03 | 0.230 |
| PDW | Seropositive RA  (FinnGen) | 322 | 1.15 (1.03, 1.28) | 0.082 | 0.288 | 1.21 (1.05, 1.39) | 0.211 | 1.82 (1.29, 2.56) | 0.018 | 1.10 (0.89, 1.36) | 0.003 | -7.72E-04 | 3.42E-03 | 0.822 |
| PDW | Seronegative RA  (FinnGen) | 322 | 0.92 (0.82, 1.02) | 0.400 | 4.43E-07 | 0.90 (0.82, 0.99) | 0.483 | 0.87 (0.78, 0.98) | 0.421 | 0.71 (0.58, 0.86) | 0.473 | -1.08E-03 | 2.72E-03 | 0.692 |

**Supplementary Table S5 Causal association between MPV and RA**

| Exposure | Outcome | SNP  (n) | IVW | | | Weighted Median | | Weighted Mode | | MR egger | | | | |
| --- | --- | --- | --- | --- | --- | --- | --- | --- | --- | --- | --- | --- | --- | --- |
|  |  |  | OR (95%CI) | *P* value | *P* heterogeneity | OR (95%CI) | *P* value | OR (95%CI) | *P* value | OR (95%CI) | *P* value | Intercept | se | *P* value |
| MPV | RA  (ukb) | 333 | 0.92 (0.84, 1.01) | 0.102 | 3.85E-205 | 0.91 (0.79, 1.05) | 0.039 | 0.92 (0.82, 1.03) | 0.027 | 0.94 (0.82, 0.98) | 3.86E-04 | 0.011 | 0.003 | 0.001 |
| MPV | Other RA (FinnGen) | 409 | 1.13 (1.01, 1.26) | 0.122 | 5.86E-05 | 1.17 (1.01, 1.36) | 0.458 | 1.09 (0.91, 1.32) | 0.653 | 1.20 (0.99, 1.47) | 0.481 | -1.98E-03 | 3.57E-03 | 0.581 |
| MPV | Seropositive RA  (FinnGen) | 409 | 1.20 (1.06, 1.35) | 0.213 | 9.67E-04 | 1.32 (1.10, 1.57) | 0.336 | 1.30 (0.99, 1.71) | 0.539 | 1.34 (1.08, 1.66) | 0.447 | 1.01E-04 | 3.48E-03 | 0.977 |
| MPV | Seronegative RA  (FinnGen) | 409 | 0.95 (0.88, 1.03) | 0.058 | 1.87E-13 | 0.94 (0.84, 1.06) | 0.145 | 0.99 (0.90, 1.08) | 0.157 | 0.94 (0.84, 1.05) | 0.198 | 8.75E-04 | 2.69E-03 | 0.745 |

**Supplementary Table S6 Causal association between RA and PCT**

| Exposure | Outcome | SNP  (n) | IVW | | | Weighted Median | | Weighted Mode | | MR egger | | | | |
| --- | --- | --- | --- | --- | --- | --- | --- | --- | --- | --- | --- | --- | --- | --- |
|  |  |  | Beta (se) | *P* value | *P* heterogeneity | Beta (se) | *P*  value | Beta (se) | *P*  value | Beta (se) | *P* value | Intercept | se | *P* value |
| RA  (ukb) | PCT | 69 | 0.011 (0.006) | 0.079 | 1.01E-238 | 0.003 (0.009) | 0.500 | 0.002 (0.003) | 0.290 | 0.002 (0.002) | 0.724 | 0.003 | 0.002 | 0.269 |
| Other RA (FinnGen) | PCT | 15 | 0.011 (0.007) | 0.149 | 2.65E-51 | 0.021 (0.017) | 4.04E-05 | 0.010 (0.002) | 0.005 | 0.008 (0.002) | 0.243 | -0.006 | 0.009 | 0.516 |
| Seropositive RA  (FinnGen) | PCT | 9 | 0.023 (0.024) | 0.107 | 3.39E-42 | 0.081 (0.026) | 4.29E-05 | 0.017 (0.004) | 0.007 | 0.014 (0.004) | 0.017 | -0.026 | 0.011 | 0.043 |
| Seronegative RA  (FinnGen) | PCT | 28 | 0.007 (0.007) | 0.338 | 4.91E-106 | 0.005 (0.012) | 2.82E-04 | 0.008 (0.002) | 0.017 | 0.005 (0.002) | 0.673 | 0.001 | 0.005 | 0.853 |

**Supplementary Table S7 Causal association between RA and PLT**

| Exposure | Outcome | SNP  (n) | IVW | | | Weighted Median | | Weighted Mode | | MR egger | | | | |
| --- | --- | --- | --- | --- | --- | --- | --- | --- | --- | --- | --- | --- | --- | --- |
|  |  |  | Beta (se) | *P* value | *P* heterogeneity | Beta (se) | *P*  value | Beta (se) | *P*  value | Beta (se) | *P* value | Intercept | se | *P* value |
| RA  (ukb) | PLT | 69 | 0.015 (0.007) | 0.020 | 8.92E-259 | 0.008 (0.010) | 0.427 | -0.003 (0.004) | 0.227 | -0.004 (0.003) | 0.419 | 0.002 | 0.002 | 0.315 |
| Other RA (FinnGen) | PLT | 15 | 0.016 (0.019) | 0.075 | 3.65E-80 | 0.026 (0.021) | 2.29E-09 | 0.017 (0.003) | 5.73E-05 | 0.014 (0.002) | 0.243 | -0.006 | 0.012 | 0.618 |
| Seropositive RA  (FinnGen) | PLT | 9 | 0.030 (0.028) | 0.093 | 4.68E-66 | 0.103 (0.032) | 8.38E-08 | 0.025 (0.005) | 6.80E-04 | 0.022 (0.004) | 0.015 | -0.033 | 0.013 | 0.039 |
| Seronegative RA  (FinnGen) | PLT | 28 | 0.010 (0.007) | 0.175 | 2.56E-108 | 0.007 (0.012) | 4.29E-04 | 0.010 (0.003) | 5.65E-04 | 0.008 (0.002) | 0.540 | 0.001 | 0.005 | 0.810 |

**Supplementary Table S8 Causal association between RA and PDW**

| Exposure | Outcome | SNP  (n) | IVW | | | Weighted Median | | Weighted Mode | | MR egger | | | | |
| --- | --- | --- | --- | --- | --- | --- | --- | --- | --- | --- | --- | --- | --- | --- |
|  |  |  | Beta (se) | *P* value | *P* heterogeneity | Beta (se) | *P*  value | Beta (se) | *P*  value | Beta (se) | *P* value | Intercept | se | *P* value |
| RA  (ukb) | PDW | 69 | -0.005 (0.004) | 0.244 | 3.36E-85 | -0.006 (0.006) | 0.281 | -0.004 (0.004) | 0.001 | -0.016 (0.005) | 0.368 | 0.001 | 0.002 | 0.862 |
| Other RA (FinnGen) | PDW | 15 | -0.011 (0.007) | 0.087 | 5.89E-37 | -0.005 (0.015) | 0.095 | -0.004 (0.002) | 0.133 | -0.004 (0.002) | 0.762 | -0.004 | 0.009 | 0.643 |
| Seropositive RA  (FinnGen) | PDW | 9 | -0.014 (0.012) | 0.240 | 1.56E-26 | -0.069 (0.019) | 0.223 | -0.006 (0.005) | 0.536 | -0.003 (0.005) | 0.008 | 0.025 | 0.008 | 0.014 |
| Seronegative RA  (FinnGen) | PDW | 28 | -0.005 (0.005) | 0.322 | 1.34E-37 | -0.002 (0.008) | 0.112 | -0.003 (0.002) | 0.295 | -0.002 (0.002) | 0.773 | -0.001 | 0.003 | 0.703 |

**Supplementary Table S9 Causal association between RA and MPV**

| Exposure | Outcome | SNP  (n) | IVW | | | Weighted Median | | Weighted Mode | | MR egger | | | | |
| --- | --- | --- | --- | --- | --- | --- | --- | --- | --- | --- | --- | --- | --- | --- |
|  |  |  | Beta (se) | *P* value | *P* heterogeneity | Beta (se) | *P*  value | Beta (se) | *P*  value | Beta (se) | *P* value | Intercept | se | *P* value |
| RA  (ukb) | MPV | 69 | -0.012 (0.006) | **0.037** | 2.43E-162 | -0.011 (0.008) | **3.17E-04** | -0.012 (0.003) | 0.290 | -0.005 (0.004) | 0.210 | 0.001 | 0.002 | 0.868 |
| Other RA  (FinnGen) | MPV | 15 | -0.015 (0.007) | **0.039** | 8.67E-42 | -0.015 (0.017) | **9.39E-07** | -0.016 (0.003) | 5.25E-04 | -0.010 (0.002) | 0.366 | 0.001 | 0.009 | 0.951 |
| Seropositive RA  (FinnGen) | MPV | 9 | -0.020 (0.022) | 0.102 | 3.57E-28 | -0.066 (0.024) | 1.25E-08 | -0.026 (0.005) | 0.001 | -0.025 (0.005) | 0.029 | 0.021 | 0.010 | 0.073 |
| Seronegative RA  (FinnGen) | MPV | 28 | -0.008 (0.005) | 0.101 | 3.73E-43 | -0.007 (0.008) | 0.227 | -0.004 (0.003) | 7.25E-04 | -0.008 (0.002) | 0.446 | -0.001 | 0.003 | 0.792 |
